# Antibacterial and anticancer activities of orphan biosynthetic gene clusters from Atlantis II Red Sea brine pool

**DOI:** 10.1186/s12934-019-1103-3

**Published:** 2019-03-18

**Authors:** Laila Ziko, Al-Hussein A. Saqr, Amged Ouf, Matthias Gimpel, Ramy K. Aziz, Peter Neubauer, Rania Siam

**Affiliations:** 1 Graduate Program of Biotechnology, School of Sciences and Engineering, The American University in Cairo, New Cairo, Cairo, 11835 Egypt; 2 Department of Biology, School of Sciences and Engineering, The American University in Cairo, SSE (Parcel 7), Second Floor, Office: Room 2194, AUC Avenue, New Cairo, Cairo, 11835 Egypt; 3 Chair of Bioprocess Engineering, Department of Biotechnology, Technische Universität Berlin, Ackerstrasse 76, ACK24, 13355 Berlin, Germany; 4 Department of Microbiology and Immunology, Faculty of Pharmacy, Cairo University, Cairo, 11562 Egypt

**Keywords:** Atlantis II Red Sea brine pool, Antibacterial, Anticancer, Orphan biosynthetic gene clusters, Specialized metabolism

## Abstract

**Background:**

Cancer and infectious diseases are problematic because of continuous emergence of drug resistance. One way to address this enormous global health threat is bioprospecting the unlikeliest environments, such as extreme marine niches, which have tremendous biodiversity that is barely explored. One such environment is the Red Sea brine pool, Atlantis II Deep (ATII). Here, we functionally screened a fosmid library of metagenomic DNA isolated from the ATII lower convective layer (LCL) for antibacterial and anticancer activities.

**Results:**

Selected clones, 14-7E and 10-2G, displayed antibacterial effects on the marine strain *Bacillus* sp. Cc6. Moreover, whole cell lysates from 14-7E and 10-2G exhibited decreased cell viability against MCF-7 (39.1% ± 6.6, 42% ± 8.1 at 50% v/v) and U2OS cells (35.7% ± 1.9, 79.9% ± 5.9 at 50% v/v), respectively. By sequencing the insert DNA from 14-7E and 10-2G, we identified two putative orphan biosynthetic gene clusters. Both clusters harbored putative ATP-binding cassette (ABC) transporter permeases and *S*-adenosylmethionine-related genes. Interestingly, the biosynthetic gene cluster identified on 14-7E is of archaeal origin and harbors a putative transcription factor. Several identified genes may be responsible for the observed antibacterial and anticancer activities. The 14-7E biosynthetic gene cluster may be encoding enzymes producing a specialized metabolite (effect of detected genes involved in C–C bond formation and glycosylation). The bioactivity may also be due to predicted subtilases encoded by this cluster. The 10-2G cluster harbored putative glycosyltransferase and non-ribosomal peptide synthase genes; thus the observed activity of this clone could be caused by a bioactive peptide.

**Conclusions:**

The ATII LCL prokaryotic metagenome hosts putative orphan biosynthetic gene clusters that confer antibiotic and anticancer effects. Further biochemical studies should characterize the detected bioactive components, and the potential use of 14-7E metabolite for antibiosis and 10-2G metabolite as a selective anti-breast cancer drug.

**Electronic supplementary material:**

The online version of this article (10.1186/s12934-019-1103-3) contains supplementary material, which is available to authorized users.

## Background

Currently the healthcare sector is seriously challenged by a rapidly increasing inefficiency of antibacterial and anticancer drugs. The last years have been referred to as the resistance or post-antibiotic era, as increasing numbers of resistant microbial strains are detected to all or most of the available antimicrobials [1]. Recent reports of resistance to colistins, last-resort antimicrobial agents, are worrying [2]. Cancer treatment is facing a similar problem, as several cancers exhibit multi-drug resistance (MDR) against anticancer drugs [3]. Consequently, there is a need for new antimicrobial and anticancer drugs that could either surmount or bypass the MDR hurdle [3].

Nature is an inexhaustible reservoir of drugs against a wide spectrum of diseases [4]. Almost 73% of the FDA-approved small molecule antibiotics and 83% of the approved small molecule anticancer agents are either natural products, their derivatives or mimics [4]. Thus, mining nature for bioactive molecules has proven valuable in investigating diverse environmental niches, and will undoubtedly shed light on new chemistries with bioactivity, specifically antibiosis and anticancer effects [3–5]. Interestingly, since the early forties some antibiotic compounds have been known to also possess anticancer activity [6]. This group of anticancer antibiotics includes drugs of diverse chemical structures, such as bleomycin, actinomycin D and doxorubicin [6, 7].

Many microbes produce bioactive compounds, known as specialized metabolites, that are not involved in their primary basic activities [8, 9], but rather confer survival advantages to the hosts in their native environment [9]. For example, in marine environments, small molecules help microbes to survive in this competitive niche by quorum quenching or by antagonism [10]. Such specialized metabolites are encoded by an assortment of genes, often arranged in the host genome as biosynthetic gene clusters (BGCs) [9]. BGCs essentially comprise contiguous genes that together encode the production of one or more related specialized metabolites [9]. These clusters are required for the synthesis of a large spectrum of structurally diverse compounds such as polyketides and non-ribosomal peptides [9, 11]. BGCs comprise genes required for the synthesis of the specialized metabolites, as well as regulatory genes and genes that confer resistance to the host against its own metabolites [9]. Computational mining for BGCs in microbial genomes can be conducted by a suite of tools, e.g. antiSMASH (the antibiotics and secondary metabolite analysis shell) [11, 12].

Microbes, the interaction of symbiotic microbes and their hosts, as well as free-living microbes in extreme conditions, all play key roles in producing new natural products of pharmacological importance [4]. Although earlier studies on microbes producing bioactive compounds were restricted to the few culturable organisms or ‘the low hanging fruits’, unculturable organisms became later on accessible by DNA sequence-based approaches [13]. Such high-throughput approaches increased our understanding of the complexity of marine microbiomes, particularly of extreme environments [13–15]. The biodiversity of biomes harboring thermophilic and marine niches is reported to be much higher than that of cultured organisms, and are thus considered hotspots to look for novel microbes and bioactive compounds [13].

Many compounds isolated from marine bacteria were effective against antibiotic-resistant strains [16]. One example is 1-acetyl-β-carboline, isolated from a *Streptomyces* species inhabiting a shallow marine sediment in Korea was effective against methicillin-resistant *Staphylococcus aureus* (MRSA) strains [16, 17]. Another example is salinilactam, that was discovered by mining the genome of the marine actinomycete *Salinispora tropica* and was found to have an antibacterial effect [18, 19]. Also, several marine products have been found to be useful in overcoming the MDR exhibited by cancer cells, such as sipholane triterpenoids isolated from the Red Sea sponge *Callyspongia siphonella*, that could overcome MDR and had anti-proliferative effects against breast cancer cell lines [3]. Another interesting example is salinosporamide K, an anticancer non-ribosomal peptide that was identified in the genome of the marine bacterium *Salinispora pacifica* [18, 20]. Several FDA-approved drugs were derived from natural products of marine origin, e.g. eribulin, a macrocyclic ketone analogue of halichondrin B that is used against metastatic breast cancer [21]. Caboxamycin, produced by a microbe living in the deep-sea sediment of the Canary basin, was active against several cancer cell lines, inhibited phosphodiesterase, and was active against several Gram-positive bacteria [22]. Until 2013, 578 natural products were isolated from deep sea inhabitants, including only 2 from Archaea and 123 from bacteria and fungi [21, 23].

Several compounds with a wide range of bioactivities were isolated from the Red Sea, that exhibit antiviral, antifungal and anti-oxidant activities [24]. The Red Sea hosts 25 deep hypersaline anoxic basins (DHABs) or brine pools [25, 26]. Extracts from microbiota inhabiting Red Sea brine pools (namely: Nereus brine, Kebrit sediment, and brine–seawater interface layers in Atlantis II, Kebrit Deep, Erba Deep, Nereus Deep and Discovery Deep), exhibited cytotoxic activity and in some cases apoptosis towards MCF-7, HeLa and DU1245 cancer cells [27, 28]. The deepest part of the Red Sea is the Atlantis II Deep Lower Convective Layer (ATII LCL), and ATII brine pool is 2194 m deep [25, 29]. It has multiple extreme conditions: high salinity (252 psu), high temperature (~ 67.1 °C) and high heavy metal content [26, 30–32]. Several enzymes have been isolated from ATII LCL, such as a thermophilic esterase [33], a nitrilase [34] and two thermostable antibiotic resistance enzymes [35]. This study uses a culture-independent approach to investigate antibacterial and anticancer activities conferred by the metagenome of the ATII LCL niche. Also, bioinformatic analysis of assembled metagenomic reads from several Red Sea brine pools unraveled 524 specialized metabolism gene clusters in ATII LCL [36]. The computational detection of potential specialized metabolism gene clusters rooted for the experimental detection of specialized metabolites in samples from the same site.

**Fig. 1:**
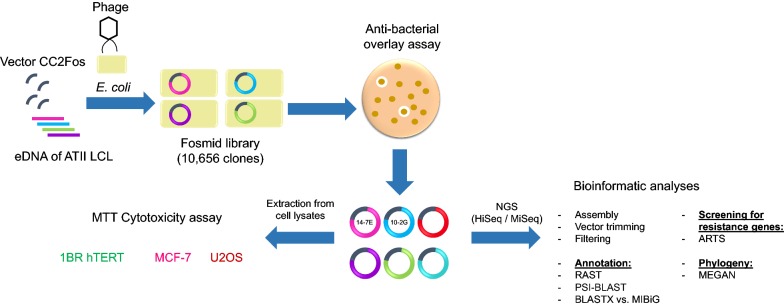
Project workflow. DNA from ATII Red Sea brine pool Lower Convective Layer (LCL) was earlier isolated and a fosmid library was constructed containing 10,656 clones [33]. An anti-bacterial overlay assay was conducted to functionally screen for antibiotic activity. Clones that exhibited zones of inhibition were further sequenced and annotated. This was followed by extraction of whole cell lysates to assess cell viability against different cell lines

Through functional screening of an ATII LCL metagenomic fosmid library, antibacterial activity and anticancer effects were assessed (Fig. 1). Sequencing and gene annotation of selected positive clones indicated potential antibacterial and anticancer activities of gene products. Accordingly, functionally screening extremophile metagenomes could be a valuable strategy to search for novel antibacterial and anticancer agents.

## Results

### Identification of antibacterial activity of Red Sea Atlantis II LCL fosmid library clones

**Fig. 2:**
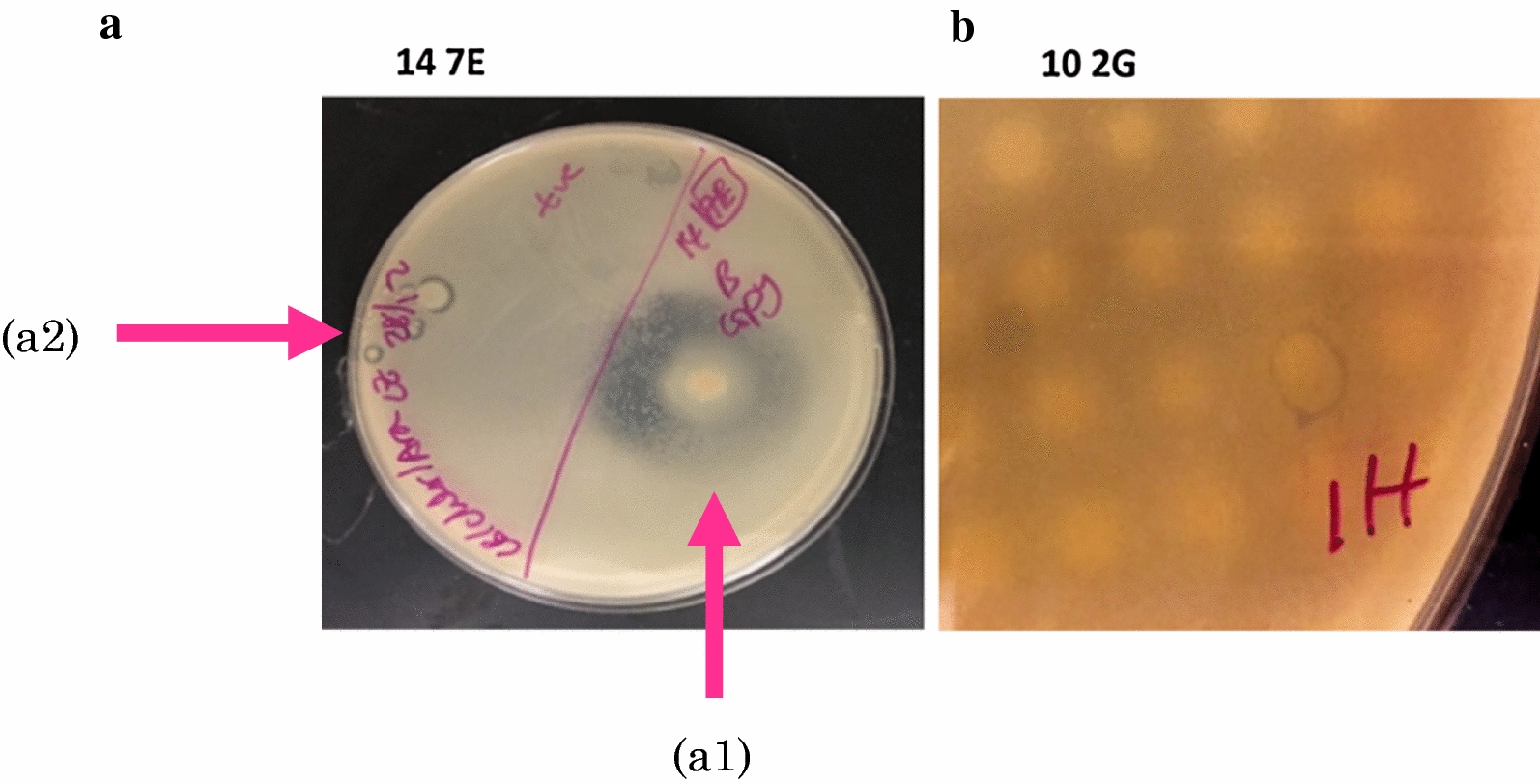
Anti-bacterial overlay assay results. Zones of inhibition of 14-7E (**a1**) and *E. coli* CBAA11 (positive control) (**a2**), against *Bacillus* sp. Cc6 are shown. **b** Portion of the 96-well plate replica showing zone of inhibition of 10-2G

Out of the 10,656 clones screened, 11 exhibited zones of inhibition, indicating antibacterial activity against *Bacillus* sp. Cc6. The largest inhibitory zones were generated by 14-7E (diameter of 1.6 cm) (Additional file 1: Figure S1-a), and 10-2G (diameter of 0.6 cm) (Fig. 2, Additional file 1: Figure S1-b1). Nine other clones also generated zones of inhibition. The positive control strain had an inhibitory zone of 0.7 cm (Additional file 1: Figure S1-b2). The diameters were measured from a single dish containing 96 clones (Additional file 1: Figure S1). For better visualization, 14-7E and the positive control were individually assessed on the same plate (Fig. 2a). Consequently, 14-7E and 10-2G were selected for further experimentation.

### Differential decrease in cell viability by selected Red Sea Atlantis II LCL fosmid library extracts

**Fig. 3:**
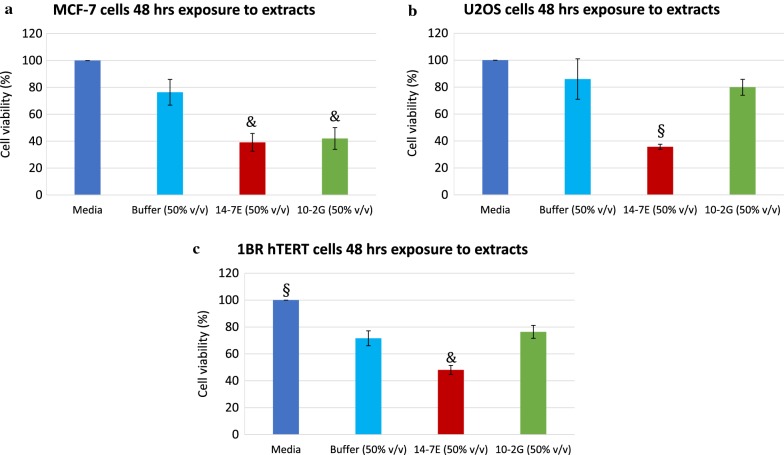
Cell viability percentage of cell lines after exposure to whole cell lysates. **a** MCF-7 cells, **b** U2OS cells and **c** 1BR hTERT cells, after 48 h exposure to 50% v/v extracts of: 14-7E (red) and 10-2G (green). Also presented are the media controls (dark blue) and 50% v/v buffer controls (light blue). The presented data for each condition is the mean of at least three independent experiments. *P* values are denoted as follows: & ≤ 0.05, # ≤ 0.01 and § ≤ 0.001

Whole cell lysates were prepared from 14-7E and 10-2G, and the protein concentrations of the resulting extracts were determined to be 472.8 µg/ml and 642.8 µg/ml, for 14-7E and 10-2G respectively. The effect of the lysates on cell viability was tested on cancerous human breast adenocarcinoma (MCF-7) and bone osteosarcoma (U2OS) cell lines as well as the non-cancerous human telomerase reverse transcriptase immortalized cell line (1BR hTERT), for 48 h (Fig. 3a–c). Generally, a dose-dependent effect was observed, as less cell viability was detected with increasing lysate concentration (Additional file 1: Figure S3a–c). For MCF-7 cells, a similar and significant decrease in cell viability was observed upon addition of either 14-7E extracts (cell viability 39.1% ± 6.6; *P* ≤ 0.05) or 10-2G extracts (cell viability 42% ± 8.1; *P* ≤ 0.05) at 50% v/v. Compared to the buffer (cell viability 76.4% ± 9.6), addition of both extracts reduced cell viability about twofold (Fig. 3a). In the case of U2OS cells, viability was only significantly decreased with the 14-7E extract (cell viability 35.7% ± 1.9; *P* ≤ 0.001), whereas the buffer control (86.0% ± 15) and 10-2G extract (cell viability 79.9 ± 5.9; *P* > 0.05) affected cell viability only marginally at 50% v/v (Fig. 3b). As putative anti-cancer drugs should specifically target cancerous cells without affecting non-cancerous cells, we used the immortalized but non-cancerous 1BR hTERT cell line for the cell viability assay. At 50% v/v, the buffer (71.6% ± 5.6) and 10-2G extract (76.4% ± 4.8; *P* > 0.05) induced only marginal decrease in cell viability, whereas cell viability decreased significantly again with 14-7E extract (48.1% ± 3.4; P ≤ 0.05) (Fig. 3c).

### Annotation of the fosmid insert DNA in antibacterial and anticancer Red Sea Atlantis II LCL clones

Both fosmid clones (14-7E and 10-2G) were deeply sequenced (~ 30,000× and 1500× coverage respectively). The generated assembled reads, following quality control, generated 29 scaffolds for 14-7E, and 14 scaffolds for 10-2G (Table 1). The number of protein-encoding genes (PEGs) detected by Rapid Annotations using Subsystems Technology (RAST) in each assembly was 289 and 30 for 14-7E and 10-2G, respectively (Table 1). The majority of PEGs encoded hypothetical proteins (90% of 14-7E PEGs and 84% of 10-2G PEGs) (Table 2, Additional file 1: Tables S1, S2).

**Table 1:** Assembly metrics of the bioactive fosmid insert DNA from Red Sea ATII LCL

Assembly statistics	14-7E insert	10-2G insert
Sequencing method	Illumina HiSeq: TruSeq DNA PCR Free (350)	Illumina MiSeq: 300 bp paired—end read (Illumina MiSeq V3)
De novo assembly program	SOAPdenovo2	CLC Genomics Workbench v 8.0
Total bases	2,044,687,834	72,217,200
Total reads	20,244,434	240,724
Total reads after quality filtering	14,876,068	240,658
Total bases in scaffolds	201,086	2,452,525
Scaffold N50	15,392	1891
Number of scaffolds	29	1318
Total bases in scaffolds after filtering and trimming	201,086	21,407
Scaffold N50 after filtering and trimming	15,392	11,374
Number of scaffolds after filtering and trimming	29	14
Number of scaffolds with RAST-annotated PEGs	27	2
Number of PEGs	289	30

**Table 2:** Annotation of selected PEGs of putative orphan biosynthetic gene clusters

Proposed functions of cluster elements	Scaffold	Start	Stop	PEG	RAST annotation	Best hit from PSI-BLAST	Query coverage %	E-value	Identity %	Accession number
(a) 14-7E selected PEGs annotation										
Regulatory gene	Scaffold_C361	395	1345	28	Transcription initiation factor B	Transcription initiation factor IIB [*candidate division MSBL1 archaeon SCGC*-*AAA259D14*]	95	3e−138	65	gi|985656859|KXA89394.1
Biosynthesis of specialized metabolite/peptide modification	Scaffold_C371	2077	3141	54	Dolichol-phosphate mannosyltransferase (EC 2.4.1.83) homolog	Dolichol monophosphate mannose synthase [*Chloroflexi bacterium CG08_land_8_20_14_0_20_45_12*]	99	9e−109	47	gi|1277809164|PIU23225.1
	Scaffold_C383	437	1396	73	Radical SAM domain protein	Radical SAM/SPASM domain-containing protein [*Thermodesulfobium narugense*]	83	2e−18	28	gi|503521935|WP_013756246.1
	Scaffold_16	18334	17825	202	Geranylgeranyl diphosphate synthase (EC 2.5.1.29)	Hypothetical protein AKJ41_03425 [*candidate division MSBL1 archaeon SCGC*-*AAA259O05*]	100	8e−80	75	gi|985669946|KXB00917.1
Subtilases	Scaffold_16	11832	13133	200	Peptidase S8 and S53, subtilisin, kexin, sedolisin	Subtilase family protein [*Micromonospora cremea*]	37	1e−11	33	gi|1118770207|SIM81338.1
	Scaffold_3	3824	3937	232	Hypothetical protein	Peptidase S8/S53 subtilisin kexin sedolisin [*Halothermothrix orenii*]	82	8e−14	35	gi|501769773|WP_012635463.1
Resistance to specialized metabolite	Scaffold_3	3169	3348	231	Hypothetical protein	ABC transporter permease [*Desulfitobacterium* sp. *PCE1*]	86	0.076*	32	gi|518688679|WP_019850372.1
	Scaffold_2	9816	8962	218	NAD-dependent glyceraldehyde-3-phosphate dehydrogenase (EC 1.2.1.12)	Glyceraldehyde-3-phosphate dehydrogenase [*candidate division MSBL1 archaeon SCGC*-*AAA261F17*]	99	4e−166	83	gi|985671588|KXB02384.1
	Scaffold_2	9970	9818	219	NAD-dependent glyceraldehyde-3-phosphate dehydrogenase (EC 1.2.1.12)	Glyceraldehyde-3-phosphate dehydrogenase [*candidate division MSBL1 archaeon SCGC*-*AAA261F17*]	92	2e−18	89	gi|985671588|KXB02384.1
(b) 10-2G selected PEGs annotation										
Synthesis of non-ribosomal peptide	Scaffold_7	29	160	20	Hypothetical protein	Non-ribosomal peptide synthetase [*Mycobacterium minnesotense*]	67	0.18*	45	gi|1178501113|WP_083022343.1
Biosynthesis of specialized metabolite/peptide modification	Scaffold_7	3719	4762	29	Glycosyltransferase	Glycosyltransferase Family 4 protein [*Enhydrobacter aerosaccus*]	98	1e−30	28	gi|1194598116|WP_085933361.1
	Scaffold_3	202	702	3	Hypothetical protein	Class I SAM-dependent methyltransferase [*Methanosarcina mazei*]	78	4e−12	34	gi|850504616|WP_048046626.1
	Scaffold_3	2297	2989	6	Glycosyltransferase	Glycosyl transferase [*Parcubacteria group bacterium CG11_big_fil_rev_8_21_14_0_20_39_14*]	77	5e−35	38	gi|1277086632|PIQ92058.1
Resistance to specialized metabolite	Scaffold_3	2961	3542	7	Hypothetical protein	ABC transporter permease [*Hymenobacter* sp. *CRA2*]	47	0.73*	27	gi|1150819492|WP_078012825.1

Annotation of the putative biosynthetic gene cluster elements in: (a) 14-7E and (b) 10-2G. * PEGs with PSI-BLAST hits of E-value > 0.005. The proposed functions of the biosynthetic gene cluster elements are denoted, each function beside its relevant annotated gene. The scaffold number is denoted, as well as the start, stop and PEG number. RAST annotation for each gene is then denoted. Lastly, PSI-BLAST best hit for each gene is denoted, its query coverage, percentage identity and best hit accession number

To gain better understanding of the PEGs, including those encoding hypothetical proteins, we used two tools for further annotation: PSI-BLAST analysis against NCBI non-redundant protein database and BLASTX against curated sequences in Minimum Information about a Biosynthetic Gene cluster (MIBiG) database. The PSI-BLAST analysis elaborated on the closest homolog of each PEG. PSI-BLAST was especially used because it is more powerful in detection of similarities between evolutionary distant protein sequences [37]. On the other hand, the BLASTX/MIBiG analysis allowed the identification of the closest characterized biosynthetic gene cluster homolog of each PEG. The PSI-BLAST analysis allowed the annotation of some hypothetical proteins that had no BLASTX hits (annotation of all PEGs is presented in Additional file 1: Tables S1, S2).

Nine PEGs in 14-7E, and five PEGS in 10-2G putatively encoded specialized metabolism genes (Table 2a, b). These genes were found to constitute interesting putative biosynthetic gene clusters (discussed below). A large number of PSI-BLAST best hits of PEGs were lacking significance (hits with E-value > 0.005). These were 187 and 15 PEGs, for 14-7E and 10-2G, respectively (denoted with asterisks in Table 2a, b, and shaded in grey in Additional file 1: Tables S1, S2).

Also, the BLASTX alignment of the PEGs against curated sequences in MIBiG, identified the closest biosynthetic gene cluster to each of the PEGs (Table 2, Additional file 1: Tables S1, S2). The MIBiG database comprises a thorough assortment of characterized biosynthetic gene clusters [38]. Seventeen PEGs identified in 14-7E resulted in hits with an E-value ≤ 0.005, while five PEGs detected in 10-2G had a hit with an E-value ≤ 0.005 (Additional file 1: Table S4). Annotation results of the BLASTX/MIBiG analysis are detailed in Additional file 1: Tables S1, S2.

### Protein-based phylogeny inference

Although the PSI-BLAST analysis cannot be used for phylogenetic inference, given that the hits are usually distant homologs, the phyla to which PSI-BLAST hits belongs can still make some suggestions about the habitats of the organisms encoding these proteins (Table 2a, b, Additional file 1: Tables S1, S2). For example, the organisms harboring PSI-BLAST hits included *Aquimarina latercula,* a marine bacterium originally isolated from the Sea of Japan [39], the halophilic and thermophilic bacterium *Halothermothrix orenii* [40], and the thermophilic bacterium *Thermoanaerobacterium* sp. *PSU*-*2* [41]. Of note, 77 of the detected PSI-BLAST best hits to 14-7E PEGs aligned with archaeal sequences (Additional file 1: Table S1), e.g., *candidate division MSBL1 archaeon SCGC*-*AAA261F19*, *candidate division MSBL1 archaeon SCGC*-*AAA385D11* (Table 2, Additional file 1: Table S1) [42]. Metagenome Analyzer (MEGAN) algorithm [43] predicted phylogenetic origins of the fosmid insert DNA in 14-7E and 10-2G as denoted in (Additional file 1: Table S3, Figure S4). Although most of the PEGs yielded no hits (261 out of 289 PEGs for 14-7E) and (27 out of 30 PEGs), eight PEGs pertaining to 14-7E were assigned to Archaea.

### Annotation of putative orphan biosynthetic gene clusters

**Fig. 4:**
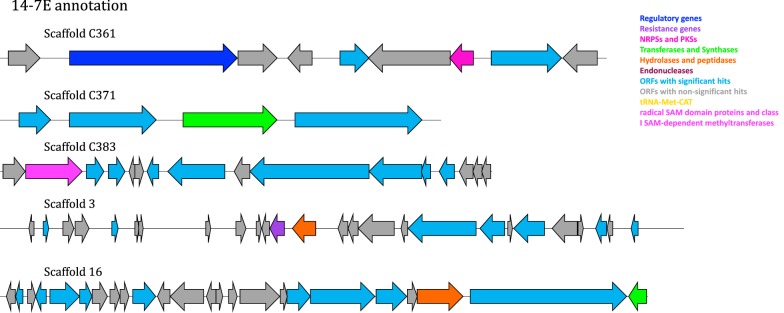
Sequence maps of the putative orphan archaeal biosynthetic gene cluster on 14-7E insert. Five selected scaffolds are depicted (scaffold C361, scaffold C371, scaffold C383, scaffold 3 and scaffold 16). Dark blue: regulatory genes, purple: resistance genes, pink: NRPSs and PKSs, Green: transferases and synthases, orange: hydrolases and peptidases, dark red: endonucleases, blue: ORFs with significant hits, grey: ORFs with non-significant hits yellow: tRNA-Met-CAT and magenta: radical SAM domain proteins and class I SAM-dependent methyltransferases

Nine PEGs in 14-7E, putatively encoding specialized metabolite genes, were identified on six of the scaffolds and were further analyzed (Table 2a). Sequence maps of the putative orphan archaeal biosynthetic gene clusters identified in 14-7E (Fig. 4) had scaffolds harboring putative biosynthetic gene cluster elements, including: (1) a transcription initiation factor IIB on scaffold C361 (65% identity) (2) a dolichol monophosphate mannose synthase on scaffold C371 (47% identity) (3) a subtilase family protein (33% identity) and a geranylgeranyl diphosphate synthase on scaffold 16, (4) a peptidase S8/S53 subtilisin kexin sedolisin (35% identity) and an ATP-binding cassette (ABC) transporter permease on scaffold 3 (32% identity), and lastly a (5) radical *S*-adenosylmethionine (SAM/SPASM) domain-containing protein (28% identity) on scaffold C383. Also, two putative NADH dehydrogenases were detected on 14-7E scaffolds as detected by the Antibiotic Resistant Target Seeker (ARTS) program (Table 2). It is likely that the former genes are part of a putative orphan archaeal biosynthetic gene cluster that includes a transcription initiation factor, two subtilases, a dolichol monophosphate mannose synthase, a geranylgeranyl diphosphate synthase, resistance genes, and a radical SAM domain-containing protein.

**Fig. 5:**
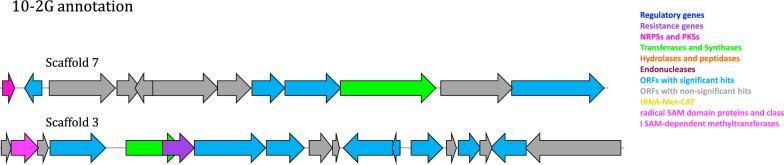
Sequence maps of the putative orphan biosynthetic gene cluster on 10-2G insert. All three annotated scaffolds are depicted (scaffold 7 and scaffold 3). Dark blue: regulatory genes, purple: resistance genes, pink: NRPSs and PKSs, Green: transferases and synthases, orange: hydrolases and peptidases, dark red: Endonucleases, blue: ORFs with significant hits, grey: ORFs with non-significant hits yellow: tRNA-Met-CAT and Magenta: radical SAM domain proteins and class I SAM-dependent methyltransferases

Similarly, five PEGs in 10-2G, encoding for specialized metabolite genes, were identified on two of the scaffolds (Table 2b). Sequence maps of the putative orphan biosynthetic gene clusters on 10-2G (Fig. 5) have the following scaffolds harboring putative biosynthetic gene cluster elements: (1) a non-ribosomal peptide synthetase (NRPS) (45% identity) and a glycosyltransferase Family 4 protein (28% identity) on scaffold 7, (2) a class I SAM-dependent methyltransferase (34% identity), a glycosyl transferase (38% identity) and an ABC transporter permease (27% identity) on scaffold 3. Lastly, the search by ARTS tool did not yield putative resistance genes. It is likely that the former genes are part of a putative orphan biosynthetic gene cluster that includes a NRPS, two glycosyltransferases, a SAM-dependent methyl transferase and a resistance gene.

## Discussion

### Antibacterial activity of Red Sea Atlantis II (ATII LCL) metagenomic library clones

We screened a prokaryotic metagenomic library of the deepest, secluded and extreme Red Sea environment, the ATII LCL, for antibacterial and anticancer effects. Eleven positive clones (out of 10,656) were identified, and two (14-7E and 10-2G) were further sequenced (Fig. 2). In congruence, Yung et al. identified three clones from two prokaryotic metagenomic libraries associated with a green alga and a marine sponge, having collectively 106,500 clones [44]. It is possible that functional screening of metagenomic libraries, using *E. coli* as the host, gives an inherently low yield of positives [13, 45]. Difficulties in heterologous expression are estimated to prevent 60% or more of the enzymes from being natively expressed [13, 45]. Heterologous expression of foreign DNA is problematic mainly because of difficulties in translation or transcription and/or the lack of the precursors [13]. The use of more than one host may improve the heterologous expression of native proteins [13].

In the current study, we used *Bacillus* sp. Cc6 strain as the challenging strain, which is a marine *Bacillus* strain that inhabits an ecological niche relatively similar to the Red Sea and its antibiotic resistance is compatible with the fosmid vector [44]. Marine bacteria are well-known producers of specialized metabolites, that assist in their competitive survival, using mechanisms such as quorum quenching and antibiotic activity [10]. Earlier, two unique antibiotic resistance enzymes were detected in the same ecosystem (ATII LCL) [35]. Although microbes living in ATII LCL site were not subjected a priori to antibiotics, they might have developed competitive advantages for better survival, such as having antibiotic resistance genes [35, 46, 47]. Perhaps the interplay between antibiotic and antibiotic-resistance genes in ATII LCL has a role in the microbial community members’ survival and communication [46].

### Anticancer effects of selected Red Sea Atlantis II (ATII LCL) metagenomic library clones

It is expensive and technically challenging to evaluate the anticancer effect for all the clones, as opposed to screening for antibiosis. By this approach, we aimed to investigate the antibacterial activity of the active clones more thoroughly, and then test for anticancer effect, due to the reason that many anticancer agents were originally discovered by having an antibacterial effect [6]. Although both 14-7E and 10-2G were originally obtained from the same ATII LCL metagenome, they resulted into differential decrease in cell viability. The effect of the extracts on mammalian cell viability was tested on breast cancer (MCF-7), osteosarcoma (U2OS) and non-cancerous fibroblast (1BR HERT) cell lines. This allowed us to compare the effect of the lysates on the viability of cancerous and non-cancerous cell lines.

Among the cancer cell lines, 10-2G extracts only decreased the cell viability of MCF-7 cells (42% ± 8.1 at 50% v/v concentration, *P* ≤ 0.05). 10-2G exhibited a selective decrease in cell viability of MCF-7 cells, since it did not significantly alter the cell viability of the non-cancerous cells and U2OS cells. On the other hand, 14-7E extracts decreased the cell viability of cancer and non-cancer cell lines at 50% v/v concentration as follows: MCF-7: 39.1% ± 6.6 (*P* ≤ 0.05), U2OS: 35.7% ± 1.9 (*P* ≤ 0.001) and 1BR hTERT: 48.1% ± 3.4 (*P* ≤ 0.05) (Fig. 3). Morphological features characteristic of cell death, were observed microscopically, when compared to control cells (Additional file 1: Figure S5). Since the detected genes were different in 14-7E and 10-2G, most likely different specialized metabolites/enzymes were possibly expressed and hence conferred differential decrease in mammalian cell viability (Table 2, Additional file 1: Tables S1, S2).

Although our cell viability assays were performed at different concentrations of extracts (i.e. 1, 5, 10, 15, 20 and 50%) (Additional file 1: Figure S3), significant differences were observed at the highest concentrations, and we therefore focus on the 50% v/v. Although 50% v/v concentration might not be physiologically achievable for the lysate, it is likely that the active molecule is diluted in the cell lysate. Large fluctuations in standard deviation values were detected for the two lowest concentrations of 14-7E lysates (1%, 5% v/v). It is likely that such large standard deviations would be due to the cell lysate containing different lysate components and different dilutions of the active molecule, which was similarly reported in earlier studies [48]. Our results indicate that 10-2G lysate would be a better candidate to look for selective anticancer effect towards breast cancer cells. Moreover, the lack of 10-2G lysate activity against non-cancerous cells seems to be beneficial as it might prevent adverse effects. The mechanism of action of the enzymes/metabolites could be further investigated, especially with regards to MDR [3]. Also, the effects on other cancer cell lines could be further conducted.

A study by Sagar et al. tested the cytotoxic effects of extracts of marine strains inhabiting similar environments, which are brine–seawater interfaces of several of Red Sea brine pools, namely: Discovery Deep, Kebrit Deep, Nereus Deep and Erba Deep [27]. The brine–seawater interfaces are unique environments, but different from the extreme brine pools, and the Atlantis II brine pool anticancer effects were not studied [25, 27]. Moreover, Sagar and coworkers cultured the microbial strains then tested the cytotoxic effects of their lipophilic and hydrophilic extracts [27]. They also performed cultures and extraction on a larger scale (i.e., larger culture volume and 2 weeks duration) [27]. Our approach has an additional advantage of capturing bioactive enzymes from the major, uncultured portion of the metagenome [49].

### Archaeal orphan biosynthetic gene cluster from ATII brine pool LCL on 14-7E

The selected clones were both sequenced by high-throughput sequencing platforms. We sequenced 201,086 bp in 14-7E scaffolds and 21,407 bp in 10-2G scaffolds. 14-7E was sequenced using HiSeq, while 10-2G was sequenced using MiSeq. Despite the different sequencing instruments, both sequencing approaches have similar chemistries. Additionally, similar *de novo* assembly methods and quality filtering were performed. Surprisingly, the number of retrieved sequences for 14-7E were larger than the expected size, and this could be attributed to the possibility that more than one fosmid insert was sequenced.

We detected gene clusters in the assembled sequences of fosmid 14-7E and considered them orphan gene clusters because the metabolites are yet to be characterized [8] (Fig. 4, Additional file 1: Figure S2). Using Sanger sequencing we confirmed one of the scaffolds (scaffold 2). The PSI-BLAST search results suggest an archaeal origin for these sequences because of the large number of hits with archaeal sequences (77 hits) (Table 2, Additional file 1: Table S1). Thirty-three PEGs had hits similar to the candidate division *Mediterranean Sea Brine Lakes 1* (*MSBL1*) archaeon, pertaining to different single-cell amplified genomes [42]. MSBL1 is an uncultured lineage and the amplified genomes from this lineage were obtained from similar environments but not including ATII LCL (sites included: ATII upper convective layer, Discovery Deep brine, ATII brine-interface of 2036 m depth, Nereus brine and Erba brine water) [42].

Out of the 33 hits, 58% were hits with Discovery brine, 18% Atlantis II brine-interface of 2036 m depth, 15% Nereus brine and 9% Erba brine [42]. In agreement, MEGAN phylogenetic analysis corroborates the conclusion that 14-7E fosmid insert DNA is still largely metagenomic dark matter-as most of the PEGs were not assigned to particular taxa (261 out of 289 PEGs (Additional file 1: Table S3, Figure S4-a). Additionally, MEGAN phylogenetic results support the possible archaeal origin of 14-7E sequences, as eight PEGs were assigned to Archaea, one PEG was assigned to the class *Halobacteria*, one PEG was assigned to *Methanococci* class, and two PEGs were assigned to *Methanomicrobia* class. One PEG was assigned to each of the following species: *Halovivax asiaticus* [50]—an extremely halophilic sediment archaeon—, *Methanococcus maripaludis* [51]—a methanogenic sediment archaeon, *Methanosarcina acetivorans* [52]—a methanogenic marine sediment archaeon—and *Methanosarcina soligelidi* [53]—a methanogenic soil archaeon.

Putative components of a BGC were identified on the fosmid insert DNA of 14-7E. First, a transcription factor was detected (on contig 361) and annotated as transcription initiation factor IIB, which is essentially required for archaeal transcription initiation [54]. One way to increase the ability of *E.* *coli* to express heterologous proteins in metagenomic libraries, is to express heterologous sigma factors [55]. Perhaps the presence of TFIIB facilitated the heterologous expression of the putative archaeal genes, although *E. coli* was the host.

Two peptidases of the subtilase family were detected (on scaffold 3 and 16), which may have contributed to the observed antibacterial and anticancer effects. Amidases could be acting as antimicrobials that break the amide bonds in the cell walls [49]. Subtilisins have shown antibiofilm activity against several species, such as *Listeria monocytogenes*, *Pseudomonas* and *Bacillus* sp. [56]. In agreement with our results, subtilases are also reported to have potent anticancer effects, especially the catalytic subunit A (SubA), and researchers are aiming to improve their specificity to cancer cells [57].

Three PEGs were annotated as putative specialized metabolite biosynthetic genes: a geranylgeranyl diphosphate synthase (on scaffold 16), a dolichol monophosphate mannose synthase (on contig 371), and a radical SAM domain protein (on contig 383). Geranylgeranyl diphosphate synthase catalyzes the condensation of the 5-Carbon ring of geranylgeranyl diphosphate of some specialized metabolites e.g. carotenoids [58]. Dolichol monophosphate mannose synthase is an enzyme involved in glycosylation and was detected in Archaea before [59]. The putative biosynthetic genes hint at the possibility that carbon rings are perhaps being formed and that glycosylation of proteins might occur. Radical SAM enzymes are key players in the post-translational modification of ribosomally synthesized and post-translationally modified peptides (RiPPs) [60]. Several RiPPs have antibacterial and anticancer activities, rendering them an interesting group of specialized metabolites [60]. SAM enzymes catalyze a lot of different reactions such as: epimerization, C–C bond formation, thioether formation, complex rearrangements and methylation [60]. Particularly, class C SAM methylases have a role in the biosynthesis of specialized metabolites with antibacterial and anticancer effects e.g. fosfomycin [61]. Consequently, the detected radical SAM domain-containing protein points towards the possibility of its role in either biosynthesis of the specialized metabolite, or post-translational modification of a synthesized RiPP [60, 61].

Finally, a ‘self-defense’ gene was annotated to be coding for an ABC transporter permease (on scaffold 3). Resistance genes are frequently encoded within the specialized metabolism gene clusters to protect the host from the natural product it synthesizes [9, 62]. ABC transporters pump unwanted compounds outside the cell, e.g. toxins [63]. Perhaps the detected ABC permease is protecting the host having the putative specialized metabolism gene cluster. To the best of our knowledge, this could be the first report of a putative orphan archaeal biosynthetic gene cluster, harbored on 14-7E, resulting from functional screening of a Red Sea brine pool metagenome. A recent study, which included 29 genomes of archaeal species, detected 414 putative BGCs [64]. Earlier, an ectoine BGC was identified in the genome of the marine archaeon *Nitrosopumilus maritimus* [65]. BGCs have been previously detected in archaeal genomes that code for a variety of molecules including terpenes, bacteriocins and NRPs [66]. In contrast to the aforementioned genomic mining studies, our study revealed an orphan archaeal BGC from a metagenomic sample. It is noteworthy that two putative NAD-dependent glyceraldehyde-3-phosphate dehydrogenases detected on scaffold 2 (Table 2), were also detected by ARTS pipeline [67]. A new strategy proved its success in characterizing the antibiotic thiotetronic acid BGC, by searching for duplicated housekeeping genes in close proximity to the BGCs [68]. Such duplicated housekeeping genes are playing protective roles to resist the action of the produced natural product on the host [67, 68]. This finding strengthens the approach to further prioritize 14-7E cluster for experimentation, as it is more likely to be producing a novel bioactive natural product. It is also likely that the duplicated housekeeping genes on 14-7E are contributing to the resistance towards the bio-active compound.

Similar studies identified putative hydrolases, serine proteases and amidases [44, 49]. In addition to subtilases, we also detected components of putative orphan biosynthetic gene clusters. Further experiments and computational analyses would attribute more specific functions to each gene in the cluster [20] (Figs. 4, 5, Additional file 1: Figure S1). However, our work paves the way for finding novel metabolites and their clusters, especially in Archaea, because of the scarcity of reports on their natural products and BGCs [23]. Several archaeocins were previously identified and a subset of them are encoded by gene clusters such as halocin C8 [69]. Significant hits with terpene, peptide, polyketide, saccharide and alkaloid classes are leads to the chemical nature of the specialized metabolite produced by 14-7E (Additional file 1: Table S4), which should be further investigated.

### Putative orphan biosynthetic gene cluster from ATII brine pool LCL on 10-2G

Another orphan gene cluster was detected within 10-2G [8]. For that cluster, however, MEGAN phylogenetic analysis was not conclusive, as 27 out of 30 PEGs had no hits to certain taxa (Additional file 1: Table S3, Figure S4-b). Four biosynthetic genes were detected: a non-ribosomal peptide synthase (NRPS) (on contig 7), a class I SAM-dependent methyltransferase (on contig 3), a glycosyltransferase Family 4 protein (on contig 7) and a glycosyltransferase (on contig 3). NRPSs are reported to produce peptides, some of which exhibit antibiotic and/or anticancer effects e.g. bleomycin and daptomycin [70]. Non-ribosomal peptides are a major class of bioactive compounds, whether antimicrobial or anticancer agents. The detected NRPS hints that 10-2G might be producing a bioactive peptide. The detected class I SAM-dependent methyltransferase perhaps is contributing in the biosynthesis of the specialized metabolite encoded by the putative gene cluster [60, 61]. Furthermore, class I SAM-dependent methyltransferases have a potential for biotechnological applications [71]. Glycosyltransferases are frequent contributors to the biosynthesis of specialized metabolites, and bioinformatic tools aim to detect them in the search of specialized metabolism genes [12, 72].

Finally, a resistance gene was detected as an ABC transporter permease (on contig 3). The function of this gene product might be the efflux of the specialized metabolite so that the host is unharmed [9, 62, 63]. The significant hits pertained to alkaloid, polyketide, saccharide and peptide classes, and the chemical nature of the specialized metabolite should be further investigated (Additional file 1: Table S4).

Future studies will determine the chemical nature of the specialized metabolite or whether an enzyme is rather acting. Additionally, different methods may be attempted to extract the specialized metabolite, e.g. such as ethyl acetate extraction method which was used in similar studies [73]. Transposon mutagenesis may be used to further decipher the essential gene(s) behind the observed activity [8, 44, 49]. Additionally, a targeted knock-down approach can be used to pinpoint the particular gene(s) responsible for the observed activities based on the current predicted functions [74, 75].

### Study limitations and future prospects

The metagenomic library phenotypic screening approach used in this study is a high-throughput method to search for specialized metabolites, yet it has limitations [13]: (i) biosynthetic genes are inherently scarce (< 2% of bacterial genome); (ii) heterologous protein expression is a challenge; and (iii) the insert size (40 kb) is much smaller than the typical BGC size (up to > 150 kb) [13]. The antibacterial overlay assay results did not distinguish whether the observed antibiosis was due to the activity of proteins/enzymes coded by the fosmid DNA, or rather due to specialized metabolites produced by BGCs within the fosmid DNA [49]. Similarly, the anticancer activity was determined by using whole cell lysates, which also contain both chemicals and proteins [76]. So, further experiments are needed to determine the chemical nature of the effective agent, i.e. whether it is an enzyme(s) or rather a chemical compound(s).

## Conclusions

In conclusion, two clones from the metagenomic library of the largest Red Sea brine pool exhibited antibacterial and anticancer effects. Sequencing and annotation of selected inserts detected orphan biosynthetic gene clusters, with the specialized metabolites yet to be characterized [8]. Interestingly, 14-7E harbored a putative archaeal orphan biosynthetic gene cluster. One of the clusters (on 14-7E) is predicted to act by producing a specialized metabolite or by the action of subtilases [56]. The second cluster (on 10-2G) is predicted to act by producing a non-ribosomal peptide. The observed antibiosis and anticancer effects of the ATII metagenomic library corroborates the approach of bioprospecting extreme environments, as it could be one of many solutions to the currently emerging antibiotic and chemotherapeutic resistance [3, 77].

## Methods

### Metagenomic fosmid library screening for antibacterial activity

Water samples from the lower convective layer (LCL) of ATII Red Sea brine pool (21° 20.72′ N and 38° 04.59′ E) was previously collected in the 2010 KAUST/WHOI/HCMR expedition [33]. Environmental DNA was extracted from the 0.1 µm filter as previously described [78] (Fig. 1). The ATII LCL fosmid library was previously constructed using pCC2FOS vector with the Copy Control Fosmid Library Production Kit (Epicenter). The library contains 10,656 clones [33]. A fresh copy of the aforementioned fosmid library was prepared prior to the downstream assays and was further used.

An antimicrobial overlay assay, similar to that reported in literature [44, 79], was used to test for antibacterial activity. For the phenotypic assay, the challenging strain was a marine *Bacillus* strain associated with the Australian marine sponge *Cymbastela concentrica*–*Bacillus* sp. Cc6 (gift from Torsten Thomas, University of New South Wales), while the positive control strain was *E.* *coli* CBAA11, which produces the antibacterial tambjamine [44, 80]. *E. coli* clones containing the fosmid library were grown on LB plates supplemented with 0.01% arabinose and 12.5 µg/ml chloramphenicol, incubated overnight at 37 °C and for an additional night at 25 °C. *Bacillus* sp. Cc6 was cultured in 100 ml LB with chloramphenicol at 37 °C with shaking until OD_600_ 0.5. The culture was diluted to 1:100 in top agar (7.5 g/l) and poured on the plates with the grown colonies [79]. The overlaid plates were incubated overnight at 25 °C and observed for clear zones in the top layer [44].

### Extract preparation

Overnight cultures (100 ml culture incubated at 37 °C with shaking) from the positive clones, previously supplemented with auto-induction solution and chloramphenicol, were centrifuged at 3500 rpm for 10 min. Afterwards, the cell pellets were re-suspended in 20 ml of 10 mM Tris–HCl pH 7. The extracts were sonicated on ice at 20% maximal amplitude for 370 s, with 10 s intervals without sonication (Branson 150D Ultrasonic Cell Disruptor with 3 mm diameter sonotrode). The extracts were finally filter-sterilized with 0.2 µm membrane filters (Corning) [76]. Protein concentrations of the extracts were determined by the Pierce™ bicinchoninic acid BCA protein assay kit (ThermoFischer).

### Cell lines and culture conditions

Three cell lines were used for the cell viability assay: a human breast adenocarcinoma cell line (MCF-7) [81], an osteosarcoma cell line (U2OS) [82] (gift from Andreas Kakarougkas, University of Sussex) and skin fibroblast cells (wild-type and non-cancerous cells) immortalized with human telomerase reverse transcriptase (1BR hTERT) [83–85]. The cells were cultured in DMEM (Lonza, Germany), supplemented with 10% fetal bovine serum (Lonza, Germany) and 5% Penicillin–Streptomycin (Lonza, Germany). All cells were grown at 37 °C in an incubator supplied with 5% CO_2_.

### Cell viability assay

The initial seeding density was adjusted to 10^4^ cells/well and left overnight to adhere to the bottom of the 96-well plates (Greiner Bio-One, Germany). The old medium was discarded, and 100 μl of fresh medium containing different concentrations (0, 1, 5, 10, 15, 20 and 50%) of the extracts were added. The percentage of remaining viable cells was assessed by the MTT assay after 48 h of exposure to the extracts. First, the medium was replaced by 100 µl fresh media supplemented with 20 µl of 5 mg/ml MTT reagent (3-(4,5-dimethylthiazolyl-2)-2,5-diphenyltetrazolium bromide, Serva, Germany). After 3 h of incubation, the medium was discarded and 100 µl DMSO (Sigma-Aldrich, USA) was added to solubilize the purple precipitates.

Negative control cells (A_595_ control) were supplemented with complete medium and a cell-free medium was used as the blank (A_595_ blank). Absorbance at 595 nm (A_595_) was measured in a SPECTROstar Nano microplate reader (BMG LabTech, Germany). The percentage of cell viability was calculated as follows:

$$Cell \,Viability\,\% = \left[ {\frac{{\left( {{\text{A}}_{595} \,{\text{sample}}{-} {\text{A}}_{595} \,{\text{blank}}} \right)}}{{\left( {{\text{A}}_{595}\,{\text{control}}{-}{\text{A}}_{595} \,{\text{blank}}} \right)}}} \right] \times 100$$

An additional buffer control experiment was conducted, by adding buffer 50% v/v to each of the three tested cell lines. The data are presented as the average of at least three independent experiments. For pairwise comparisons between the values, one-way ANOVA test was conducted, followed by post hoc Tukey test. The shown *P* values represent the significant differences between the mean of each condition and the mean of the negative control cells with buffer concentration 50% v/v (& *P* ≤ 0.05, # *P* ≤ 0.01 and § *P* ≤ 0.001). The ANOVA, post hoc test and *P* value calculation were conducted using the R program version 3.3.1 (R Development Core Team 2016).

### Sequencing and bioinformatics

Two clones (14-7E and 10-2G) were selected for fosmid DNA extraction followed by sequencing. Overnight cultures were supplemented with auto-inducer/chloramphenicol. Fosmid DNA was extracted by QIAprep Spin Miniprep Kit (Qiagen). The 14-7E fosmid DNA was sequenced by the Illumina HiSeq 2000 100 bp paired-end read platform (Macrogen, Republic of Korea), while 10-2G fosmid DNA was sequenced by the Illumina MiSeq V3 300 bp paired-end read platform (LGC, Germany). After sequencing and quality filtering, the reads were assembled by the de novo assembly programs SOAPdenovo2 [86] and the CLC Genomics Workbench v 8.0 assembler (Qiagen), respectively (Table 1).

Prior to annotation, the vector sequences (pCC2FOS™) were trimmed from the resulting scaffolds. *E. coli* sequence reads were also filtered out. *E. coli* NC_010473 DH10B served as the reference sequence, because the EPI300™-T1R Phage T1-resistant *E. coli* strain, derived from *E. coli* DH10B, was used for the fosmid library construction. Putative PEGs were determined in the resulting scaffolds with the RAST platform [87]. Each PEG was further compared to sequences in the publicly available databases by PSI-BLAST [37]. The PEGs were also compared to the protein sequences curated in the MIBiG database by BLASTX [38]. Phylogenetic origins of the PEGs of 14-7E and 10-2G fosmid insert DNA were predicted by MEGAN algorithm by using BLASTX results against nr database and using default parameters [43]. Lastly, the scaffold sequences were screened for resistance genes including housekeeping genes that are duplicated within BGCs. The search for putative resistance genes was conducted by using ARTS tool [67].

## Additional file

**Additional file 1: Figure S1.** Anti-bacterial overlay assay results on 96-well plates. Zones of inhibition of 14-7E (a), 10-2G (b1) and *E. coli* CBAA11 (positive control) (b2), against *Bacillus* sp. Cc6. A single petri dish contained 96 clones. **Figure S2.** Sequence maps of all the putative PEGs on 14-7E insert. Dark blue: regulatory genes, purple: resistance genes, pink: NRPSs and PKSs, Green: transferases and synthases, orange: hydrolases and peptidases, dark red: Endonucleases, blue: ORFs with significant hits, grey: ORFs with non-significant hits yellow: tRNA-Met-CAT and magenta: radical SAM domain proteins and class I SAM-dependent methyltransferases. **Figure S3.** Cell viability percentage of cell lines after exposure to selected whole cell lysates. (A) MCF-7 cells, (B) U2OS cells and (C) 1BR hTERT cells, after 48 h exposure to extracts of: 14-7E (red) and 10-2G (green). Also presented are the media controls (dark blue) and 50% v/v buffer controls (light blue). The x-axis indicates the concentrations of the whole cell extracts (%v/v). The presented data for each condition is the mean of at least three independent experiments. *P* values are denoted as follows: & ≤ 0.05, # ≤ 0.01 and § ≤ 0.001. **Figure S4.** Phylogenetic trees as predicted by MEGAN for the insert DNA of (a) 14-7E and (b) 10-2G. **Figure S5.** Representative photos of MCF-7 (A) and 1BR hTERT (B) cells after exposure to 14-7E lysates for 48 h (200× magnification).

## Abbreviations

ABC: ATP-binding cassette
antiSMASH: antibiotics and secondary metabolite analysis shell
ARTS: Antibiotic Resistant Target Seeker
ATII: Atlantis II Deep
BGCs: biosynthetic gene clusters
DHABs: deep hypersaline anoxic basins
LCL: lower convective layer
MDR: multi-drug resistance
MIBiG: Minimum Information about a Biosynthetic Gene cluster
MEGAN: Metagenome Analyzer
MRSA: methicillin-resistant *Staphylococcus aureus*
MSBL1: Mediterranean Sea Brine Lakes 1
NRPS: non-ribosomal peptide synthetase
PEG: protein-encoding gene
RAST: Rapid Annotations using Subsystems Technology
RiPP: ribosomally synthesized and post-translationally modified peptide
SAM: *S*-adenosylmethionine
SubA: subunit A
