# Supplementary material for: Antibacterial and anticancer activities of orphan biosynthetic gene clusters from Atlantis II Red Sea brine pool
Source: Microb Cell Fact. 2019 Mar 18;18:56. doi: 10.1186/s12934-019-1103-3 (PMC6423787; doi:10.1186/s12934-019-1103-3)
Supplement: Supplementary file 1 — Additional file 1: Figure S1. Anti-bacterial overlay assay results on 96-well plates. Zones of inhibition of 14-7E (a), 10-2G (b1) and E. coli CBAA11 (positive control) (b2), against Bacillus sp. Cc6. A single petri dish contained 96 clones. Figure S2. Sequence maps of all the putative PEGs on 14-7E insert. Dark blue: regulatory genes, purple: resistance genes, pink: NRPSs and PKSs, Green: transferases and synthases, orange: hydrolases and peptidases, dark red: Endonucleases, blue: ORFs with significant hits, grey: ORFs with non-significant hits yellow: tRNA-Met-CAT and magenta: radical SAM domain proteins and class I SAM-dependent methyltransferases. Figure S3. Cell viability percentage of cell lines after exposure to selected whole cell lysates. (A) MCF-7 cells, (B) U2OS cells and (C) 1BR hTERT cells, after 48 h exposure to extracts of: 14-7E (red) and 10-2G (green). Also presented are the media controls (dark blue) and 50% v/v buffer controls (light blue). The x-axis indicates the concentrations of the whole cell extracts (%v/v). The presented data for each condition is the mean of at least three independent experiments. P values are denoted as follows: & ≤ 0.05, # ≤ 0.01 and § ≤ 0.001. Figure S4. Phylogenetic trees as predicted by MEGAN for the insert DNA of (a) 14-7E and (b) 10-2G. Figure S5. Representative photos of MCF-7 (A) and 1BR hTERT (B) cells after exposure to 14-7E lysates for 48 h (200× magnification). [file 12934_2019_1103_MOESM1_ESM.docx]

**Additional material**

**Figure S1: Anti-bacterial overlay assay results on 96-well plates.** Zones of inhibition of 14-7E (a), 10-2G (b1) and *E. coli* CBAA11 (positive control) (b2), against *Bacillus* sp. Cc6. A single petri dish contained 96 clones.


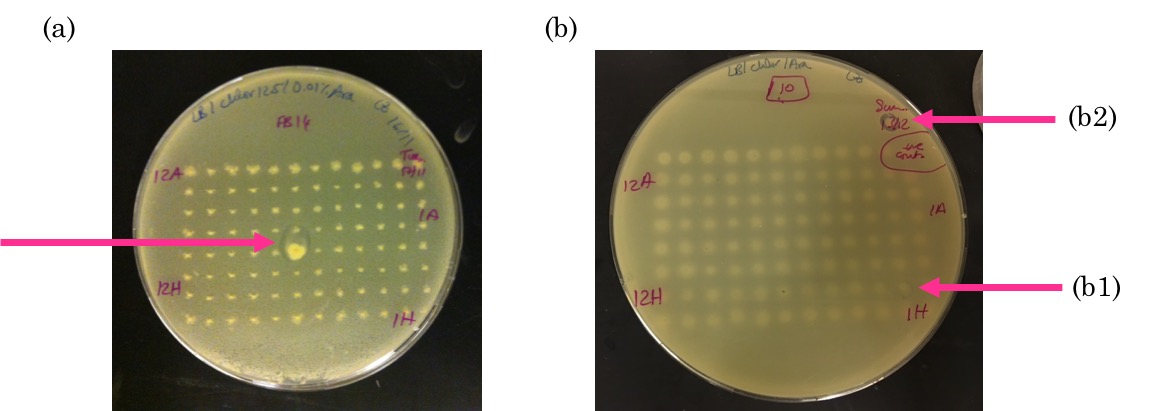


**Figure S2: Sequence maps of all the putative PEGs on 14-7E insert.** Dark blue: regulatory genes, purple: resistance genes, pink: NRPSs and PKSs, Green: transferases and synthases, orange: hydrolases and peptidases, dark red: Endonucleases, blue: ORFs with significant hits, grey: ORFs with non-significant hits yellow: tRNA-Met-CAT and Magenta: radical SAM domain proteins and class I SAM-dependent methyltransferases.

**Figure S3: Cell viability percentage of cell lines after exposure to selected whole cell lysates.** (A) MCF-7 cells, (B) U2OS cells and (C) 1BR hTERT cells, after 48 hr exposure to extracts of: 14-7E (red) and 10-2G (green). Also presented are the media controls (dark blue) and 50% v/v buffer controls (light blue). The x-axis indicates the concentrations of the whole cell extracts (%v/v). The presented data for each condition is the mean of at least three independent experiments. *P* values are denoted as follows: & ≤ 0.05, # ≤ 0.01 and § ≤ 0.001.

**Table S1: Annotation of all PEGs on 14-7E.** Three annotation tools were used: RAST (Aziz et al., 2008), psi-BLAST (Altschul et al., 1997) and BLASTX against MIBiG curated sequences (Medema et al., 2015) are depicted. Shaded in grey are PEGs with psi-BLAST hits of E-value > 0.005.

| **Scaffold** | **Start** | **Stop** | **PEG** | **RAST annotation** | **Putative gene** | **Query coverage** | **E-value** | **Identity %** | **Accession number** | **PEG length (bp)** | **MIBIG hit** | **BGC reference in MIBiG** | **Organism** | **Score** | **E-Value** | **Other** |
| --- | --- | --- | --- | --- | --- | --- | --- | --- | --- | --- | --- | --- | --- | --- | --- | --- |
| Scaffold_C311 | 421 | 197 | 1 | hypothetical protein | hypothetical protein [*Haloferax sp. Q22*] | 52% | 0.87 | 42% | gi\|1177515633\|WP_082677708.1 | 225 | BGC0000703\|c1\|100646-101065\|-\|no_locus_tag\|hypothetical_protein\|BAE95504.1 Length=139 | Kanamycin biosynthetic gene cluster | Streptomyces kanamyceticus  Bacteria; Actinobacteria; Actinobacteridae; Actinomycetales;  Streptomycineae; Streptomycetaceae; Streptomyces. | 26.2 | 2.1 | Identities = 17/45 (38%), Positives = 21/45 (47%), Gaps = 3/45 (7%)  Frame = +1 |
| Scaffold_C343 | 137 | 331 | 2 | hypothetical protein | hypothetical protein AKJ61_00845 [*candidate divison MSBL1 archaeon SCGC-AAA259B11*] | 93% | 0.013 | 27% | gi\|985657941\|KXA90379.1 | 195 | BGC0000565\|c1\|4772-6979\|+\|no_locus_tag\|goadsporin_biosynthetic_protein\|BAE46919.1 Length=735 | Goadsporin biosynthetic gene cluster | Streptomyces sp. TP-A0584  Bacteria; Actinobacteria; Actinobacteridae; Actinomycetales;  Streptomycineae; Streptomycetaceae; Streptomyces. | 27.7 | 0.58 | Identities = 13/25 (52%), Positives = 16/25 (64%), Gaps = 2/25 (8%)  Frame = +1 |
| Scaffold_C343 | 699 | 382 | 3 | hypothetical protein | YjeF protein 3 [*Heterocephalus glaber*] | 50% | 0.27 | 36% | gi\|351695538\|EHA98456.1 | 318 | BGC0001031\|c1\|7252-18210\|+\|no_locus_tag\|polyketide_synthase\|AFX60309.1 Length=3652 | Oocydin A biosynthetic gene cluster | Serratia marcescens  Bacteria; Proteobacteria; Gammaproteobacteria; Enterobacteriales;  Enterobacteriaceae; Serratia. | 27.3 | 3 | Identities = 15/40 (38%), Positives = 21/40 (53%), Gaps = 5/40 (13%)  Frame = -1 |
| Scaffold_C343 | 676 | 1293 | 4 | hypothetical protein | multifunctional nuclease/2',3'-cyclic-nucleotide 2'-phosphodiesterase/5'-nucleotidase/3'-nucleotidase [*Microbacterium sp. CH1*] | 80% | 7e-06 | 33% | gi\|1011816254\|WP_062635874.1 | 618 | BGC0001308\|c1\|5960-7231\|+\|IE98_RS42480\|hypothetical_protein\|WP_043174234.1 Length=423 | Simocyclinone D9 / simocyclinone D10 / simocyclinone D11  biosynthetic gene cluster | Streptomyces sp. NRRL B-24484  Bacteria; Actinobacteria; Streptomycetales; Streptomycetaceae;  Streptomyces. | 31.2 | 0.55 | Identities = 19/54 (35%), Positives = 25/54 (46%), Gaps = 1/54 (2%)  Frame = +1 |
| Scaffold_C343 | 1853 | 1344 | 5 | Octaprenyl diphosphate synthase (EC 2.5.1.90) / Dimethylallyltransferase (EC 2.5.1.1) / Geranyltranstransferase (EC 2.5.1.10) / Geranylgeranyl diphosphate synthase (EC 2.5.1.29) | hypothetical protein AKJ41_03425 [*candidate divison MSBL1 archaeon SCGC-AAA259O05*] | 100% | 2e-79 | 75% | gi\|985669946\|KXB00917.1 | 510 | BGC0000648\|c1\|1058-2143\|+\|no_locus_tag\|geranylgeranyl_pyrophosphate_synthetase\|CAA79955.1 Length=361 | Carotenoid biosynthetic gene cluster | Myxococcus xanthus  Bacteria; Proteobacteria; Deltaproteobacteria; Myxococcales;  Cystobacterineae; Myxococcaceae; Myxococcus. | 82.4 | 5.00E-19 | Identities = 43/108 (40%), Positives = 60/108 (56%), Gaps = 3/108 (3%)  Frame = +1 |
| Scaffold_C349 | 2136 | 1894 | 6 | hypothetical protein | hypothetical protein PYDG_00047 [*Pseudoalteromonas phage pYD6-A*] | 61% | 3.8 | 28% | gi\|472340734\|YP_007674256.1 | 243 | BGC0000027\|c1\|66477-67841\|-\|2380\|hypothetical_protein\|AGN71622.1 Length=454 | Azaphilone biosynthetic gene cluster | Monascus pilosus  Eukaryota; Fungi; Dikarya; Ascomycota; Pezizomycotina;  Eurotiomycetes; Eurotiomycetidae; Eurotiales; Aspergillaceae;  Monascus. | 28.1 | 0.61 | Identities = 15/33 (45%), Positives = 17/33 (52%), Gaps = 3/33 (9%)  Frame = +3 |
| Scaffold_C353 | 141 | 260 | 7 | hypothetical protein | No significant similarity found. |  |  |  |  | 120 | BGC0001210\|c1\|5567-8788\|-\|bpmyx0001_45470\|hypothetical_protein\|EEM14529.1 Length=1073 | Pseudomycoicidin biosynthetic gene cluster | Bacillus pseudomycoides DSM 12442  Bacteria; Firmicutes; Bacilli; Bacillales; Bacillaceae; Bacillus. | 28.1 | 0.14 | Identities = 10/26 (38%), Positives = 17/26 (65%), Gaps = 0/26 (0%)  Frame = +3 |
| Scaffold_C353 | 1837 | 1962 | 8 | hypothetical protein | No significant similarity found. |  |  |  |  | 126 | BGC0001089\|c1\|47347-63648\|+\|no_locus_tag\|hybrid_NRPS/PKS\|CAG23960.2 Length=5433 | Bacillaene biosynthetic gene cluster | Bacillus amyloliquefaciens subsp. plantarum str. FZB42  Bacteria; Firmicutes; Bacilli; Bacillales; Bacillaceae; Bacillus. | 25.4 | 1.4 | Identities = 7/23 (30%), Positives = 17/23 (74%), Gaps = 0/23 (0%)  Frame = -1 |
| Scaffold_C355 | 6 | 596 | 9 | Integrase-recombinase protein XERCD family | Phage integrase family protein [*Dehalogenimonas formicexedens*] | 93% | 5e-47 | 47% | gi\|1132445260\|APV44476.1 | 591 | BGC0001381\|c1\|18872-19354\|+\|no_locus_tag\|hypothetical_protein\|AJO72711.1 Length=160 | Brasilinolide biosynthetic gene cluster | Nocardia terpenica  Bacteria; Actinobacteria; Corynebacteriales; Nocardiaceae; Nocardia. | 31.2 | 0.33 | Identities = 15/38 (39%), Positives = 21/38 (55%), Gaps = 1/38 (3%)  Frame = +1 |
| Scaffold_C355 | 820 | 1131 | 10 | hypothetical protein | hypothetical protein emb_1c0597 [*Coriobacteriaceae bacterium EMTCatB1*] | 35% | 2e-12 | 70% | gi\|1122514007\|GAV31985.1 | 312 | BGC0000352\|c1\|16075-19320\|-\|ACIAD2772\|putative_non-ribosomal_peptide_synthetase_with_condensation,_AMP-binding_and_phosphopantetheine-binding_domains\|CAG69516.1 Length=1081 | Fimsbactin biosynthetic gene cluster | Acinetobacter sp. ADP1  Bacteria; Proteobacteria; Gammaproteobacteria; Pseudomonadales;  Moraxellaceae; Acinetobacter. | 28.1 | 1.4 | Identities = 21/87 (24%), Positives = 35/87 (40%), Gaps = 6/87 (7%)  Frame = -2 |
| Scaffold_C355 | 1466 | 1128 | 11 | hypothetical protein | hypothetical protein [*Archaeoglobus profundus*] | 57% | 2e-06 | 41% | gi\|502704606\|WP_012939856.1 | 339 | BGC0000981\|c1\|25587-30590\|-\|no_locus_tag\|polyketide_synthase\|ADF88277.1 Length=1667 | Toyocamycin biosynthetic gene cluster | Streptomyces ahygroscopicus subsp. wuzhouensis  Bacteria; Actinobacteria; Actinobacteridae; Actinomycetales;  Streptomycineae; Streptomycetaceae; Streptomyces. | 26.6 | 6.3 | Identities = 21/57 (37%), Positives = 28/57 (49%), Gaps = 1/57 (2%)  Frame = +1 |
| Scaffold_C355 | 2221 | 2030 | 12 | hypothetical protein | hypothetical protein FOXG_14201 [*Fusarium oxysporum f. sp. lycopersici 4287*] | 92% | 5.2 | 38% | gi\|1070361176\|XP_018253879.1 | 192 | BGC0001041\|c1\|32605-32760\|+\|Strop_1033\|hypothetical_protein\|ABP53507.1 Length=51 | Salinosporamide biosynthetic gene cluster | Salinispora tropica CNB-440  Bacteria; Actinobacteria; Actinobacteridae; Actinomycetales;  Micromonosporineae; Micromonosporaceae; Salinispora. | 28.1 | 0.11 | Identities = 12/21 (57%), Positives = 13/21 (62%), Gaps = 0/21 (0%)  Frame = +1 |
| Scaffold_C355 | 2740 | 2573 | 13 | hypothetical protein | WLM domain-domain-containing protein [*Aspergillus candidus*] | 96% | 0.41 | 41% | gi\|1317901917\|PLB36457.1 | 168 | BGC0000605\|c1\|2943-5087\|+\|no_locus_tag\|lantibiotic-type_dehydratase\|AEM00617.1 Length=714 | GE37468 biosynthetic gene cluster | Streptomyces sp. ATCC 55365  Bacteria; Actinobacteria; Actinobacteridae; Actinomycetales;  Streptomycineae; Streptomycetaceae; Streptomyces. | 27.7 | 0.38 | Identities = 13/39 (33%), Positives = 17/39 (44%), Gaps = 0/39 (0%)  Frame = +1 |
| Scaffold_C357 | 320 | 78 | 14 | hypothetical protein | 16S rRNA (uracil(1498)-N(3))-methyltransferase [*Oribacterium sinus*] | 68% | 0.15 | 39% | gi\|494270725\|WP_007156532.1 | 243 | BGC0000117\|c1\|56417-70474\|+\|no_locus_tag\|modular_polyketide_synthase\|BAB69195.1 Length=4685 | Oligomycin biosynthetic gene cluster | Streptomyces avermitilis  Bacteria; Actinobacteria; Actinobacteridae; Actinomycetales;  Streptomycineae; Streptomycetaceae; Streptomyces. | 28.5 | 0.5 | Identities = 13/30 (43%), Positives = 20/30 (67%), Gaps = 2/30 (7%)  Frame = +1 |
| Scaffold_C357 | 336 | 533 | 15 | hypothetical protein | PREDICTED: mitogen-activated protein kinase 11 [*Saimiri boliviensis boliviensis*] | 66% | 2.2 | 40% | gi\|725580211\|XP_010341869.1 | 198 | BGC0001101\|c1\|15681-16277\|-\|no_locus_tag\|putative_tetR-family_transcriptional_regulator\|AAN85486.1 Length=198 | Leinamycin biosynthetic gene cluster | Streptomyces atroolivaceus  Bacteria; Actinobacteria; Actinobacteridae; Actinomycetales;  Streptomycineae; Streptomycetaceae; Streptomyces. | 28.9 | 0.22 | Identities = 12/26 (46%), Positives = 17/26 (65%), Gaps = 0/26 (0%)  Frame = +1 |
| Scaffold_C357 | 945 | 679 | 16 | hypothetical protein | PREDICTED: melanoma-associated antigen 8-like [*Octodon degus*] | 97% | 1.3 | 30% | gi\|820999737\|XP_004648573.2 | 267 | BGC0001233\|c1\|21744-38087\|+\|no_locus_tag\|non-ribosomal_peptide_synthase\|ALK27914.1 Length=5447 | Feglymycin biosynthetic gene cluster | Streptomyces sp. DSM 11171  Bacteria; Actinobacteria; Streptomycetales; Streptomycetaceae;  Streptomyces. | 29.6 | 0.27 | Identities = 13/20 (65%), Positives = 13/20 (65%), Gaps = 0/20 (0%)  Frame = +3 |
| Scaffold_C357 | 2497 | 1034 | 17 | hypothetical protein | MULTISPECIES: methylmalonyl Co-A mutase-associated GTPase MeaB [*Bacillus*] | 15% | 0.69 | 28% | gi\|1246853214\|WP_096437675.1 | 1464 | BGC0000081\|c1\|72974-74854\|+\|no_locus_tag\|radical_SAM_C-methyltransferase\|AFV52163.1 Length=626 | Kedarcidin biosynthetic gene cluster | Streptoalloteichus sp. ATCC 53650  Bacteria; Actinobacteria; Actinobacteridae; Actinomycetales;  Pseudonocardineae; Pseudonocardiaceae; Streptoalloteichus. | 30.8 | 2.7 | Identities = 23/81 (28%), Positives = 41/81 (51%), Gaps = 11/81 (14%)  Frame = +1 |
| Scaffold_C357 | 3057 | 2572 | 18 | hypothetical protein | hypothetical protein AYO20_00344 [*Fonsecaea nubica*] | 52% | 0.11 | 33% | gi\|1248736091\|XP_022505620.1 | 486 | BGC0000345\|c1\|13453-19515\|+\|no_locus_tag\|EpnH\|AHB38509.1 Length=2020 | Eponemycin biosynthetic gene cluster | Streptomyces hygroscopicus  Bacteria; Actinobacteria; Actinobacteridae; Actinomycetales;  Streptomycineae; Streptomycetaceae; Streptomyces. | 29.6 | 1.2 | Identities = 15/48 (31%), Positives = 25/48 (52%), Gaps = 0/48 (0%)  Frame = +1 |
| Scaffold_C359 | 22 | 162 | 19 | hypothetical protein | methyltransferase domain-containing protein [*Pedobacter sp. Hv1*] | 86% | 3.4 | 35% | gi\|941472720\|WP_055130295.1 | 141 | BGC0000178\|c1\|10135-18318\|+\|no_locus_tag\|polyketide_synthase\|AEC04356.1 Length=2727 | Elansolid biosynthetic gene cluster | Chitinophaga sancti  Bacteria; Bacteroidetes; Sphingobacteriia; Sphingobacteriales;  Chitinophagaceae; Chitinophaga. | 25.8 | 1.3 | Identities = 11/31 (35%), Positives = 17/31 (55%), Gaps = 0/31 (0%)  Frame = +1 |
| Scaffold_C359 | 188 | 439 | 20 | hypothetical protein | hypothetical protein [*Mycoplasma crocodyli*] | 85% | 0.14 | 30% | gi\|502819680\|WP_013054656.1 | 252 | BGC0001392\|c1\|18665-20242\|-\|Cpin_5331\|drug_resistance_transporter,_EmrB/QacA_subfamily\|ACU62762.1 Length=525 | Pinensins biosynthetic gene cluster | Chitinophaga pinensis DSM 2588  Bacteria; Bacteroidetes; Sphingobacteriia; Sphingobacteriales;  Chitinophagaceae; Chitinophaga. | 27.3 | 1.2 | Identities = 14/33 (42%), Positives = 20/33 (61%), Gaps = 0/33 (0%)  Frame = -1 |
| Scaffold_C359 | 541 | 744 | 21 | hypothetical protein | hypothetical protein AKJ65_08090 [*candidate divison MSBL1 archaeon SCGC-AAA259E19*] | 98% | 4e-10 | 46% | gi\|985659833\|KXA92072.1 | 204 | BGC0001032\|c1\|28221-48065\|+\|no_locus_tag\|polyketide_synthase\|AFX60336.1 Length=6614 | Oocydin A biosynthetic gene cluster | Serratia plymuthica  Bacteria; Proteobacteria; Gammaproteobacteria; Enterobacteriales;  Enterobacteriaceae; Serratia. | 28.5 | 0.31 | Identities = 20/58 (34%), Positives = 28/58 (48%), Gaps = 15/58 (26%)  Frame = +1 |
| Scaffold_C359 | 934 | 1068 | 22 | hypothetical protein | hypothetical protein JCHSAcid_15200 [*uncultured Acidilobus sp. JCHS*] | 100% | 2e-06 | 43% | gi\|557078280\|ESQ24117.1 | 135 | BGC0000613\|c1\|14252-15586\|+\|no_locus_tag\|peptidase\|ACS83788.1 Length=444 | Thiomuracins biosynthetic gene cluster | Nonomuraea sp. Bp3714-39  Bacteria; Actinobacteria; Actinobacteridae; Actinomycetales;  Streptosporangineae; Streptosporangiaceae; Nonomuraea. | 25.4 | 1.6 | Identities = 12/21 (57%), Positives = 13/21 (62%), Gaps = 1/21 (5%)  Frame = -2 |
| Scaffold_C359 | 1071 | 1184 | 23 | hypothetical protein | No significant similarity found. |  |  |  |  | 114 | BGC0001208\|c2\|15039-29504\|+\|H045_07040\|amino_acid_adenylation_protein\|AGE25480.1 Length=4821 | Poaeamide biosynthetic gene cluster | Pseudomonas poae RE*1-1-14  Bacteria; Proteobacteria; Gammaproteobacteria; Pseudomonadales;  Pseudomonadaceae; Pseudomonas. | 25 | 1.6 | Identities = 11/23 (48%), Positives = 15/23 (65%), Gaps = 0/23 (0%)  Frame = -2 |
| Scaffold_C359 | 1433 | 1510 | **RNA.1** | tRNA-Met-CAT | **tRNA-Met-CAT (annotation by RAST, not by psi-BLAST)** |  |  |  |  |  |  |  |  |  |  |  |
| Scaffold_C359 | 2221 | 2682 | 24 | hypothetical protein | LAFE_0E10374g1_1 [*Lachancea fermentati*] | 97% | 0.23 | 26% | gi\|1092387644\|SCW01929.1 | 462 | BGC0001000\|c1\|38547-39881\|+\|no_locus_tag\|HctH\|AAY42400.1 Length=444 | Hectochlorin biosynthetic gene cluster | Lyngbya majuscula  Bacteria; Cyanobacteria; Oscillatoriophycideae; Oscillatoriales;  Lyngbya. | 31.2 | 0.32 | Identities = 20/55 (36%), Positives = 25/55 (45%), Gaps = 3/55 (5%)  Frame = -1 |
| Scaffold_C359 | 2914 | 3114 | 25 | hypothetical protein | hypothetical protein BCR44DRAFT_277786 [*Catenaria anguillulae PL171*] | 48% | 0.14 | 44% | gi\|1183514646\|ORZ37870.1 | 201 | BGC0000193\|c1\|1-1986\|-\|no_locus_tag\|AknN\|AAF73450.1 Length=662 | Aclacinomycin biosynthetic gene cluster | Streptomyces galilaeus  Bacteria; Actinobacteria; Actinobacteridae; Actinomycetales;  Streptomycineae; Streptomycetaceae; Streptomyces. | 25.4 | 3.3 | Identities = 11/32 (34%), Positives = 18/32 (56%), Gaps = 2/32 (6%)  Frame = +1 |
| Scaffold_C359 | 3107 | 3268 | 26 | hypothetical protein | hypothetical protein Y032_0266g683 [*Ancylostoma ceylanicum*] | 94% | 3.3 | 42% | gi\|597837574\|EYB87199.1 | 162 | BGC0000021\|c1\|65477-81277\|-\|no_locus_tag\|polyketide_synthase_type_I\|AEP40936.1 Length=5266 | Apoptolidin biosynthetic gene cluster | Nocardiopsis sp. FU40  Bacteria; Actinobacteria; Actinobacteridae; Actinomycetales;  Streptosporangineae; Nocardiopsaceae; Nocardiopsis. | 28.1 | 0.21 | Identities = 11/28 (39%), Positives = 20/28 (71%), Gaps = 0/28 (0%)  Frame = +1 |
| Scaffold_C361 | 47 | 226 | 27 | hypothetical protein | hypothetical protein SAMN04487948_12912 [*Halogranum amylolyticum*] | 74% | 0.81 | 41% | gi\|1103313132\|SEP26485.1 | 180 | BGC0000273\|c1\|28239-29417\|+\|no_locus_tag\|O-methyl_transferase\|CAJ42340.1 Length=392 | Steffimycin biosynthetic gene cluster | Streptomyces steffisburgensis  Bacteria; Actinobacteria; Actinobacteridae; Actinomycetales;  Streptomycineae; Streptomycetaceae; Streptomyces. | 25.8 | 1.9 | Identities = 10/25 (40%), Positives = 15/25 (60%), Gaps = 0/25 (0%)  Frame = -1 |
| Scaffold_C361 | 395 | 1345 | 28 | Transcription initiation factor B | transcription initiation factor IIB [*candidate divison MSBL1 archaeon SCGC-AAA259D14*] | 95% | 3e-138 | 65% | gi\|985656859\|KXA89394.1 | 951 | BGC0000993\|c1\|27503-33181\|+\|no_locus_tag\|DepE\|ABP57749.1 Length=1892 | FK228 biosynthetic gene cluster | Chromobacterium violaceum  Bacteria; Proteobacteria; Betaproteobacteria; Neisseriales;  Chromobacteriaceae; Chromobacterium. | 29.3 | 4.7 | Identities = 21/79 (27%), Positives = 33/79 (42%), Gaps = 18/79 (23%)  Frame = +1 |
| Scaffold_C361 | 1350 | 1571 | 29 | hypothetical protein | membrane protein [uncultured organism] | 47% | 0.009 | 51% | gi\|452077110\|AGF93079.1 | 222 | BGC0001042\|c1\|85823-96040\|+\|no_locus_tag\|type_I_polyketide_synthase\|ACY06290.1 Length=3405 | Sanglifehrin A biosynthetic gene cluster | Streptomyces flaveolus  Bacteria; Actinobacteria; Actinobacteridae; Actinomycetales;  Streptomycineae; Streptomycetaceae; Streptomyces. | 26.9 | 1.3 | Identities = 15/40 (38%), Positives = 18/40 (45%), Gaps = 12/40 (30%)  Frame = +1 |
| Scaffold_C361 | 1769 | 1632 | 30 | hypothetical protein | Serine chemoreceptor protein [*Labrenzia alba*] | 82% | 1.8 | 49% | gi\|930851733\|CTQ52056.1 | 138 | BGC0000229\|c1\|12497-13717\|+\|no_locus_tag\|chain_length_determinant\|AGO50611.1 Length=406 | Grincamycin biosynthetic gene cluster | Streptomyces lusitanus  Bacteria; Actinobacteria; Actinobacteridae; Actinomycetales;  Streptomycineae; Streptomycetaceae; Streptomyces. | 27.3 | 0.35 | Identities = 10/18 (56%), Positives = 15/18 (83%), Gaps = 0/18 (0%)  Frame = -1 |
| Scaffold_C361 | 1928 | 2092 | 31 | hypothetical protein | hypothetical protein AKJ66_01420 [*candidate divison MSBL1 archaeon SCGC-AAA259E22*] | 100% | 2e-13 | 50% | gi\|985661700\|KXA93684.1 | 165 | BGC0000128\|c1\|31895-32701\|+\|no_locus_tag\|transcriptional_regulatory_protein\|AAY59074.1 Length=268 | Pyoluteorin biosynthetic gene cluster | Pseudomonas sp. M18  Bacteria; Proteobacteria; Gammaproteobacteria; Pseudomonadales;  Pseudomonadaceae; Pseudomonas. | 26.9 | 0.66 | Identities = 16/40 (40%), Positives = 22/40 (55%), Gaps = 0/40 (0%)  Frame = +1 |
| Scaffold_C361 | 2553 | 2089 | 32 | hypothetical protein | metallophosphoesterase [*Sphingobacteriaceae bacterium GW460-11-11-14-LB5*] | 65% | 0.89 | 27% | gi\|1197332626\|WP_086546200.1 | 465 | BGC0000222\|c1\|8465-9724\|+\|no_locus_tag\|putative_cytochrome_P450\|BAJ52675.1 Length=419 | FD-594 biosynthetic gene cluster | Streptomyces sp. TA-0256  Bacteria; Actinobacteria; Actinobacteridae; Actinomycetales;  Streptomycineae; Streptomycetaceae; Streptomyces. | 27.7 | 4.2 | Identities = 16/46 (35%), Positives = 23/46 (50%), Gaps = 0/46 (0%)  Frame = -2 |
| Scaffold_C361 | 2684 | 2550 | 33 | hypothetical protein | non-ribosomal peptide synthetase [*Clostridium aurantibutyricum*] | 52% | 8.2 | 52% | gi\|1149923283\|WP_077853227.1 | 135 | BGC0000297\|c1\|23429-24685\|-\|no_locus_tag\|putative_glycosyltransferase\|CAM59608.1 Length=418 | Aeruginoside biosynthetic gene cluster | Planktothrix agardhii NIVA-CYA 126  Bacteria; Cyanobacteria; Oscillatoriophycideae; Oscillatoriales;  Planktothrix. | 25 | 2.2 | Identities = 10/19 (53%), Positives = 11/19 (58%), Gaps = 0/19 (0%)  Frame = -3 |
| Scaffold_C361 | 2786 | 3184 | 34 | hypothetical protein | hypothetical protein [*Alkalibacterium subtropicum*] | 74% | 0.003 | 35% | gi\|1223492678\|WP_091529977.1 | 399 | BGC0001057\|c1\|27570-34937\|+\|no_locus_tag\|polyketide_synthase\|ABB90283.1 Length=2345 | Zearalenone biosynthetic gene cluster | Fusarium graminearum  Eukaryota; Fungi; Dikarya; Ascomycota; Pezizomycotina;  Sordariomycetes; Hypocreomycetidae; Hypocreales; Nectriaceae;  Fusarium. | 28.1 | 2.9 | Identities = 11/20 (55%), Positives = 16/20 (80%), Gaps = 0/20 (0%)  Frame = -1 |
| Scaffold_C361 | 3386 | 3192 | 35 | hypothetical protein | cardiolipin synthase B [*Halomonas pantelleriensis*] | 54% | 2.1 | 47% | gi\|1221518913\|WP_089659031.1 | 195 | BGC0000327\|c1\|14971-16104\|-\|SAMR0912\|hypothetical_protein\|CAJ88621.1 Length=377 | Congocidine biosynthetic gene cluster | Streptomyces ambofaciens ATCC 23877  Bacteria; Actinobacteria; Actinobacteridae; Actinomycetales;  Streptomycineae; Streptomycetaceae; Streptomyces. | 27.3 | 0.8 | Identities = 12/31 (39%), Positives = 18/31 (58%), Gaps = 0/31 (0%)  Frame = +1 |
| Scaffold_C363 | 91 | 321 | 36 | hypothetical protein | putative membrane protein [*Candidatus Nomurabacteria bacterium GW2011_GWD2_39_12*] | 84% | 0.003 | 36% | gi\|818431191\|KKR02232.1 | 231 | BGC0000033\|c1\|89143-90348\|-\|no_locus_tag\|CalG4\|AAM70365.1 Length=401 | Calicheamicin biosynthetic gene cluster | Micromonospora echinospora  Bacteria; Actinobacteria; Actinobacteridae; Actinomycetales;  Micromonosporineae; Micromonosporaceae; Micromonospora. | 25.8 | 3.2 | Identities = 10/26 (38%), Positives = 13/26 (50%), Gaps = 0/26 (0%)  Frame = +2 |
| Scaffold_C363 | 588 | 394 | 37 | hypothetical protein | No significant similarity found. |  |  |  |  | 195 | BGC0001128\|c1\|36-15686\|-\|plu3263\|\|CAE15637.1 Length=5216 | Luminmide biosynthetic gene cluster | Photorhabdus luminescens subsp. laumondii TTO1  Bacteria; Proteobacteria; Gammaproteobacteria; Enterobacteriales;  Enterobacteriaceae; Photorhabdus. | 31.2 | 0.031 | Identities = 26/63 (41%), Positives = 32/63 (51%), Gaps = 11/63 (17%)  Frame = -1 |
| Scaffold_C363 | 725 | 976 | 38 | hypothetical protein | ribonuclease Y [*Candidatus Levybacteria bacterium RIFCSPHIGHO2_01_FULL_36_15b*] | 66% | 0.011 | 39% | gi\|1083619581\|OGH11706.1 | 252 | BGC0001018\|c1\|23920-28080\|+\|no_locus_tag\|nonribosomal_peptide_synthetase\|BAH22765.1 Length=1386 | Micropeptin biosynthetic gene cluster | Microcystis aeruginosa K-139  Bacteria; Cyanobacteria; Oscillatoriophycideae; Chroococcales;  Microcystis. | 26.6 | 2.3 | Identities = 11/32 (34%), Positives = 20/32 (63%), Gaps = 0/32 (0%)  Frame = -3 |
| Scaffold_C363 | 1322 | 948 | 39 | hypothetical protein | PREDICTED: HEAT repeat-containing protein 5B isoform X5 [*Nicrophorus vespilloides*] | 88% | 1.3 | 29% | gi\|1059408698\|XP_017777277.1 | 375 | BGC0001059\|c1\|24505-25722\|+\|no_locus_tag\|ZmaF\|AAD40110.1 Length=405 | Zwittermycin A biosynthetic gene cluster | Bacillus cereus  Bacteria; Firmicutes; Bacilli; Bacillales; Bacillaceae; Bacillus;  Bacillus cereus group. | 29.6 | 0.53 | Identities = 27/110 (25%), Positives = 50/110 (45%), Gaps = 14/110 (13%)  Frame = -1 |
| Scaffold_C363 | 1694 | 1386 | 40 | hypothetical protein | autophagy-related cysteine peptidase atg4, putative [*Hammondia hammondi*] | 88% | 0.043 | 30% | gi\|675128264\|XP_008886019.1 | 309 | BGC0001165\|c1\|36316-42915\|+\|no_locus_tag\|CurH\|AAT70103.1 Length=2199 | Curacin biosynthetic gene cluster | Lyngbya majuscula  Bacteria; Cyanobacteria; Oscillatoriophycideae; Oscillatoriales;  Lyngbya. | 28.5 | 0.96 | Identities = 13/25 (52%), Positives = 14/25 (56%), Gaps = 0/25 (0%)  Frame = -1 |
| Scaffold_C363 | 2205 | 1735 | 41 | hypothetical protein | glycerol dehydrogenase [*Halarchaeum acidiphilum*] | 80% | 0.049 | 30% | gi\|545902496\|WP_021779575.1 | 471 | BGC0000210\|c1\|23643-24395\|-\|no_locus_tag\|membrane_protein\|CAE17539.1 Length=250 | Chromomycin A3 biosynthetic gene cluster | Streptomyces griseus subsp. griseus  Bacteria; Actinobacteria; Actinobacteridae; Actinomycetales;  Streptomycineae; Streptomycetaceae; Streptomyces. | 28.5 | 2.2 | Identities = 14/38 (37%), Positives = 19/38 (50%), Gaps = 0/38 (0%)  Frame = +1 |
| Scaffold_C363 | 2571 | 2251 | 42 | hypothetical protein | HAD family hydrolase [*Mycobacterium vulneris*] | 79% | 4.1 | 34% | gi\|1045344087\|WP_065462665.1 | 321 | BGC0001335\|c1\|7660-9102\|-\|no_locus_tag\|tryptophan_oxidase\|AGI62213.1 Length=480 | Borregomycin biosynthetic gene cluster | uncultured bacterium  Bacteria; environmental samples. | 30 | 0.3 | Identities = 16/49 (33%), Positives = 24/49 (49%), Gaps = 0/49 (0%)  Frame = +1 |
| Scaffold_C363 | 3102 | 2662 | 43 | hypothetical protein | ribonuclease Y [*Candidatus Nomurabacteria bacterium RIFCSPHIGHO2_01_FULL_39_9*] | 74% | 0.028 | 30% | gi\|1083781922\|OGI61270.1 | 441 | BGC0000843\|c1\|2653-4011\|-\|SCLAV_2922\|Putative_pyridoxal_phosphate-dependent_aminotransferase\|EFG07993.1 Length=452 | Clavams biosynthetic gene cluster | Streptomyces clavuligerus ATCC 27064  Bacteria; Actinobacteria; Actinobacteridae; Actinomycetales;  Streptomycineae; Streptomycetaceae; Streptomyces. | 28.5 | 2.1 | Identities = 15/38 (39%), Positives = 21/38 (55%), Gaps = 2/38 (5%)  Frame = +1 |
| Scaffold_C363 | 3532 | 3104 | 44 | hypothetical protein | restriction endonuclease subunit S [*Hydrogenophaga taeniospiralis*] | 95% | 2.4 | 28% | gi\|1056747327\|WP_068170662.1 | 429 | BGC0000429\|c1\|84713-85858\|-\|no_locus_tag\|putative_DNA-binding_protein\|AEA30291.1 Length=381 | Skyllamycin biosynthetic gene cluster | Streptomyces sp. Acta 2897  Bacteria; Actinobacteria; Actinobacteridae; Actinomycetales;  Streptomycineae; Streptomycetaceae; Streptomyces. | 26.9 | 5.5 | Identities = 25/82 (30%), Positives = 34/82 (41%), Gaps = 4/82 (5%)  Frame = +1 |
| Scaffold_C369 | 400 | 164 | 45 | hypothetical protein | aldolase [*Nonomuraea pusilla*] | 65% | 0.73 | 41% | gi\|1223058779\|WP_091100088.1 | 237 | BGC0001212\|c1\|35165-45550\|+\|no_locus_tag\|non-ribosomal_peptide_synthase\|ALD82526.1 Length=3461 | annocystin biosynthetic gene cluster | Nannocystis sp. MB1016  Bacteria; Proteobacteria; Deltaproteobacteria; Myxococcales;  Nannocystineae; Nannocystaceae; Nannocystis. | 27.7 | 0.86 | Identities = 13/39 (33%), Positives = 20/39 (51%), Gaps = 0/39 (0%)  Frame = +1 |
| Scaffold_C369 | 1312 | 1160 | 46 | hypothetical protein | 30S ribosomal protein S14 [*Enterococcus faecalis*] | 100% | 3.5 | 38% | gi\|895826113\|WP_048948179.1 | 153 | BGC0000388\|c1\|10069-18312\|+\|no_locus_tag\|mannopeptimycin_peptide_synthetase_MppA\|AAU34202.1 Length=2747 | Mannopeptimycin biosynthetic gene cluster | Streptomyces hygroscopicus  Bacteria; Actinobacteria; Actinobacteridae; Actinomycetales;  Streptomycineae; Streptomycetaceae; Streptomyces. | 25 | 2.9 | Identities = 15/36 (42%), Positives = 19/36 (53%), Gaps = 0/36 (0%)  Frame = +1 |
| Scaffold_C369 | 1476 | 1324 | 47 | hypothetical protein | No significant similarity found. |  |  |  |  | 153 | BGC0000863\|c1\|13522-16560\|+\|no_locus_tag\|polyunsaturated_fatty_acid_synthase\|ABF00130.1 Length=1012 | Eicosapentaenoic acid biosynthetic gene cluster | Pseudoalteromonas sp. DS-12  Bacteria; Proteobacteria; Gammaproteobacteria; Alteromonadales;  Pseudoalteromonadaceae; Pseudoalteromonas. | 27.3 | 0.44 | Identities = 10/24 (42%), Positives = 16/24 (67%), Gaps = 0/24 (0%)  Frame = -3 |
| Scaffold_C369 | 2356 | 2057 | 48 | hypothetical protein | polyprenol monophosphomannose synthase [*Nocardiopsis ganjiahuensis*] | 94% | 3.5 | 33% | gi\|516163599\|WP_017588659.1 | 300 | BGC0000804\|c1\|4857-7985\|-\|SMCF_977\|alpha-amylase_ScatZ1\|EHN79424.1 Length=1042 | Acarviostatin biosynthetic gene cluster | Streptomyces coelicoflavus ZG0656  Bacteria; Actinobacteria; Actinobacteridae; Actinomycetales;  Streptomycineae; Streptomycetaceae; Streptomyces. | 25.8 | 8 | Identities = 14/39 (36%), Positives = 20/39 (51%), Gaps = 3/39 (8%)  Frame = +3 |
| Scaffold_C369 | 3202 | 3390 | 49 | hypothetical protein | hypothetical protein [*Candidatus Chloroploca asiatica*] | 80% | 1e-05 | 44% | gi\|1258453861\|WP_097650322.1 | 189 | BGC0000148\|c1\|59803-76569\|+\|no_locus_tag\|polyketide_synthase_extender_modules_8-10\|AAG23262.1 Length=5588 | Spinosad biosynthetic gene cluster | Saccharopolyspora spinosa  Bacteria; Actinobacteria; Actinobacteridae; Actinomycetales;  Pseudonocardineae; Pseudonocardiaceae; Saccharopolyspora. | 28.9 | 0.16 | Identities = 13/30 (43%), Positives = 17/30 (57%), Gaps = 0/30 (0%)  Frame = -2 |
| Scaffold_C369 | 4007 | 4153 | 50 | hypothetical protein | No significant similarity found. |  |  |  |  | 147 | BGC0001192\|c1\|15150-18458\|+\|no_locus_tag\|PmxB\|AJM89734.1 Length=1102 | Colistin biosynthetic gene cluster | Paenibacillus alvei  Bacteria; Firmicutes; Bacilli; Bacillales; Paenibacillaceae;  Paenibacillus. | 27.7 | 0.3 | Identities = 12/25 (48%), Positives = 16/25 (64%), Gaps = 0/25 (0%)  Frame = +1 |
| Scaffold_C369 | 4150 | 4587 | 51 | hypothetical protein | hypothetical protein BGN82_08610 [*Alphaproteobacteria bacterium 65-7*] | 66% | 1.3 | 29% | gi\|1113123555\|OJT99191.1 | 438 | BGC0000098\|c1\|1535-9615\|-\|no_locus_tag\|polyketide_synthase\|ABA02240.1 Length=2547 | Monacolin K biosynthetic gene cluster | Monascus pilosus  Eukaryota; Fungi; Dikarya; Ascomycota; Pezizomycotina;  Eurotiomycetes; Eurotiomycetidae; Eurotiales; Aspergillaceae;  Monascus. | 31.2 | 0.31 | Identities = 14/44 (32%), Positives = 25/44 (57%), Gaps = 0/44 (0%)  Frame = +3 |
| Scaffold_C371 | 217 | 573 | 52 | hypothetical protein | hypothetical protein [*Bacillus clausii*] | 73% | 3e-05 | 37% | gi\|1236609581\|WP_094979054.1 | 357 | BGC0001117\|c1\|11170-11868\|-\|no_locus_tag\|putative_thioesterase\|CBZ42142.1 Length=232 | Himastatin biosynthetic gene cluster | Streptomyces himastatinicus ATCC 53653  Bacteria; Actinobacteria; Actinobacteridae; Actinomycetales;  Streptomycineae; Streptomycetaceae; Streptomyces. | 28.1 | 1.4 | Identities = 16/48 (33%), Positives = 25/48 (52%), Gaps = 1/48 (2%)  Frame = -1 |
| Scaffold_C371 | 789 | 1772 | 53 | hypothetical protein | hypothetical protein AMJ56_01640 [*Anaerolineae bacterium SG8_19*] | 90% | 2e-10 | 23% | gi\|931427643\|KPK13576.1 | 984 | BGC0000845\|c1\|14421-15797\|+\|SSCG_00160\|beta-lactamase\|EDY47132.1 Length=458 | Clavulanic acid biosynthetic gene cluster | Streptomyces clavuligerus ATCC 27064  Bacteria; Actinobacteria; Actinobacteridae; Actinomycetales;  Streptomycineae; Streptomycetaceae; Streptomyces. | 29.3 | 4.2 | Identities = 17/43 (40%), Positives = 24/43 (56%), Gaps = 6/43 (14%)  Frame = +1 |
| Scaffold_C371 | 2077 | 3141 | 54 | Dolichol-phosphate mannosyltransferase (EC 2.4.1.83) homolog | dolichol monophosphate mannose synthase [*Chloroflexi bacterium CG08_land_8_20_14_0_20_45_12*] | 99% | 9e-109 | 47% | gi\|1277809164\|PIU23225.1 | 1065 | GC0000388\|c1\|4359-5636\|+\|no_locus_tag\|polyprenyl_mannose_synthase_MppG\|AAU34198.1 Length=425 | Mannopeptimycin biosynthetic gene cluster | Streptomyces hygroscopicus  Bacteria; Actinobacteria; Actinobacteridae; Actinomycetales;  Streptomycineae; Streptomycetaceae; Streptomyces. | 125 | 2.00E-32 | Identities = 99/263 (38%), Positives = 146/263 (56%), Gaps = 19/263 (7%)  Frame = +1 |
| Scaffold_C371 | 3346 | 4785 | 55 | conserved protein | hypothetical protein [*Halalkalicoccus jeotgali*] | 97% | 6e-37 | 26% | gi\|495691755\|WP_008416334.1 | 1440 | BGC0000700\|c1\|50534-51037\|-\|no_locus_tag\|hypothetical_protein\|CAI59987.1 Length=167 | Istamycin biosynthetic gene cluster | Streptomyces tenjimariensis  Bacteria; Actinobacteria; Actinobacteridae; Actinomycetales;  Streptomycineae; Streptomycetaceae; Streptomyces. | 30.4 | 1.8 | Identities = 13/34 (38%), Positives = 21/34 (62%), Gaps = 0/34 (0%)  Frame = +1 |
| Scaffold_C379 | 146 | 6 | 56 | hypothetical protein | hypothetical protein [*Natronorubrum bangense*] | 95% | 1e-04 | 43% | gi\|492957717\|WP_006065802.1 | 141 | BGC0000424\|c1\|38384-39388\|-\|Hsero_2347\|acetyltransferases,_including_N-acetylases_of_ribosomal_proteins\|ADJ63846.1 Length=334 | Serobactins biosynthetic gene cluster | Herbaspirillum seropedicae SmR1  Bacteria; Proteobacteria; Betaproteobacteria; Burkholderiales;  Oxalobacteraceae; Herbaspirillum. | 26.9 | 0.49 | Identities = 11/21 (52%), Positives = 13/21 (62%), Gaps = 0/21 (0%)  Frame = -3 |
| Scaffold_C379 | 1377 | 277 | 57 | hypothetical protein | PREDICTED: probable serine/threonine-protein kinase kinX [*Lates calcarifer*] | 77% | 2e-07 | 26% | gi\|1079787834\|XP_018518905.1 | 1101 | BGC0000595\|c1\|16470-20519\|+\|SCO2450\|Ser/Thr_protein_kinase_(regulator)\|NP_626693.1 Length=1349 | SCO-2138 biosynthetic gene cluster | Streptomyces coelicolor A3(2)  Bacteria; Actinobacteria; Actinobacteridae; Actinomycetales;  Streptomycineae; Streptomycetaceae; Streptomyces; Streptomyces  albidoflavus group. | 35 | 0.11 | Identities = 33/113 (29%), Positives = 48/113 (42%), Gaps = 19/113 (17%)  Frame = +1 |
| Scaffold_C379 | 1809 | 1534 | 58 | hypothetical protein | proline--tRNA ligase [*Chryseobacterium hungaricum*] | 85% | 0.24 | 32% | gi\|1221747315\|WP_089874552.1 | 276 | BGC0001032\|c1\|18577-19881\|+\|no_locus_tag\|flavin-dependent_monooxygenase\|AFX60333.1 Length=434 | Oocydin A biosynthetic gene cluster | Serratia plymuthica  Bacteria; Proteobacteria; Gammaproteobacteria; Enterobacteriales;  Enterobacteriaceae; Serratia. | 26.2 | 4.7 | Identities = 11/34 (32%), Positives = 17/34 (50%), Gaps = 1/34 (3%)  Frame = -1 |
| Scaffold_C379 | 3721 | 1802 | 59 | DNA primase (EC 2.7.7.-) | hypothetical protein B6U99_07805 [*Candidatus Geothermarchaeota archaeon ex4572_27*] | 48% | 1e-27 | 28% | gi\|1249057761\|PCN49804.1 | 1920 | BGC0001381\|c1\|14638-15429\|+\|no_locus_tag\|IclR_family_transcriptional_regulator\|AJO72706.1 Length=263 | Brasilinolide biosynthetic gene cluster | Nocardia terpenica  Bacteria; Actinobacteria; Corynebacteriales; Nocardiaceae; Nocardia. | 32 | 1.1 | Identities = 19/48 (40%), Positives = 25/48 (52%), Gaps = 5/48 (10%)  Frame = +1 |
| Scaffold_C379 | 4101 | 3874 | 60 | hypothetical protein | hypothetical protein AKJ56_01330 [*candidate divison MSBL1 archaeon SCGC-AAA382N08*] | 84% | 6e-05 | 39% | gi\|985678424\|KXB08423.1 | 228 | BGC0001178\|c1\|72450-72812\|+\|no_locus_tag\|Chorismate_mutase\|AGS77337.1 Length=120 | UK-68,597 biosynthetic gene cluster | Actinoplanes sp. ATCC 53533  Bacteria; Actinobacteria; Actinobacteridae; Actinomycetales;  Micromonosporineae; Micromonosporaceae; Actinoplanes. | 25 | 5.6 | Identities = 13/53 (25%), Positives = 26/53 (49%), Gaps = 3/53 (6%)  Frame = +1 |
| Scaffold_C379 | 4362 | 4204 | 61 | hypothetical protein | peptidase S41 [*Pelagibacteraceae bacterium TMED170*] | 88% | 1.7 | 43% | gi\|1200509506\|OUW23128.1 | 159 | BGC0000398\|c1\|24120-38810\|+\|no_locus_tag\|nonribosomal_peptide_synthetase\|AFH75330.1 Length=4896 | Orfamide biosynthetic gene cluster | Pseudomonas sp. CMR12a  Bacteria; Proteobacteria; Gammaproteobacteria; Pseudomonadales;  Pseudomonadaceae; Pseudomonas. | 25.4 | 2.3 | Identities = 9/14 (64%), Positives = 12/14 (86%), Gaps = 0/14 (0%)  Frame = +1 |
| Scaffold_C379 | 4824 | 4414 | 62 | Very-short-patch mismatch repair endonuclease (G-T specific) | very short patch repair endonuclease [*Methanosarcina soligelidi*] | 98% | 3e-25 | 42% | gi\|850531552\|WP_048051503.1 | 411 | BGC0000229\|c1\|32089-33624\|+\|no_locus_tag\|carboxyl_transferase\|AGO50629.1 Length=511 | Grincamycin biosynthetic gene cluster | Streptomyces lusitanus  Bacteria; Actinobacteria; Actinobacteridae; Actinomycetales;  Streptomycineae; Streptomycetaceae; Streptomyces. | 27.7 | 3.8 | Identities = 10/22 (45%), Positives = 15/22 (68%), Gaps = 0/22 (0%)  Frame = +1 |
| Scaffold_C379 | 6112 | 4808 | 63 | DNA-cytosine methyltransferase (EC 2.1.1.37) | DNA cytosine methyltransferase [*Halorubrum sodomense*] | 98% | 5e-48 | 35% | gi\|1224908361\|WP_092924335.1 | 1305 | BGC0000974\|c1\|51991-52713\|+\|no_locus_tag\|O-methyltransferase\|CBD77751.1 Length=240 | Crocacin biosynthetic gene cluster | Chondromyces crocatus  Bacteria; Proteobacteria; Deltaproteobacteria; Myxococcales;  Sorangiineae; Polyangiaceae; Chondromyces. | 33.9 | 0.16 | Identities = 15/41 (37%), Positives = 22/41 (54%), Gaps = 0/41 (0%)  Frame = +1 |
| Scaffold_C379 | 6363 | 6130 | 64 | hypothetical protein | PREDICTED: NADH dehydrogenase [ubiquinone] iron-sulfur protein 5-like [*Rhinopithecus roxellana*] | 67% | 1.1 | 40% | gi\|724805094\|XP_010386677.1 | 234 | BGC0001401\|c1\|1-2053\|+\|no_locus_tag\|putative_halogenase\|AEM76785.1 Length=522 | Melleolides biosynthetic gene cluster | Armillaria mellea  Eukaryota; Fungi; Dikarya; Basidiomycota; Agaricomycotina;  Agaricomycetes; Agaricomycetidae; Agaricales; Physalacriaceae;  Armillaria. | 26.6 | 1.9 | Identities = 13/29 (45%), Positives = 14/29 (48%), Gaps = 0/29 (0%)  Frame = +2 |
| Scaffold_C379 | 6518 | 6360 | 65 | hypothetical protein | restriction endonuclease [*Hymenobacter sp. CRA2*] | 71% | 1.2 | 47% | gi\|1150819157\|WP_078012490.1 | 159 | BGC0001350\|c1\|59325-78353\|+\|no_locus_tag\|PKS\|AMH40443.1 Length=6342 | Phormidolide biosynthetic gene cluster | Leptolyngbya sp. ISBN3-Nov-94-8  Bacteria; Cyanobacteria; Oscillatoriophycideae; Oscillatoriales;  Leptolyngbya. | 25.8 | 1.6 | Identities = 11/38 (29%), Positives = 24/38 (63%), Gaps = 0/38 (0%)  Frame = -1 |
| Scaffold_C381 | 1327 | 1046 | 66 | hypothetical protein | amino acid permease [*Streptomyces scabiei*] | 87% | 0.91 | 34% | gi\|972520994\|WP_059078868.1 | 282 | BGC0001039\|c1\|19866-27221\|+\|no_locus_tag\|putative_peptide_synthetase\|AEF33080.1 Length=2451 | Pyridomycin biosynthetic gene cluster | Streptomyces pyridomyceticus  Bacteria; Actinobacteria; Actinobacteridae; Actinomycetales;  Streptomycineae; Streptomycetaceae; Streptomyces. | 25.8 | 7.4 | Identities = 14/36 (39%), Positives = 20/36 (56%), Gaps = 0/36 (0%)  Frame = -1 |
| Scaffold_C381 | 3080 | 1350 | 67 | hypothetical protein | DUF3494 domain-containing protein [*Salinimicrobium xinjiangense*] | 58% | 1e-58 | 37% | gi\|916598274\|WP_051205365.1 | 1731 | BGC0000700\|c1\|27460-28293\|-\|no_locus_tag\|putative_N-methyltransferase\|CAH60154.1 Length=277 | Istamycin biosynthetic gene cluster | Streptomyces tenjimariensis  Bacteria; Actinobacteria; Actinobacteridae; Actinomycetales;  Streptomycineae; Streptomycetaceae; Streptomyces. | 33.9 | 0.28 | Identities = 20/77 (26%), Positives = 34/77 (44%), Gaps = 0/77 (0%)  Frame = +1 |
| Scaffold_C381 | 4771 | 3107 | 68 | hypothetical protein | hypothetical protein AYK21_02985 [*Thermoplasmatales archaeon SG8-52-2*] | 70% | 3e-35 | 33% | gi\|1008839869\|KYK23215.1 | 1665 | BGC0000172\|c1\|4864-5841\|+\|P615_14830\|hypothetical_protein\|ERM18787.1 Length=325 | Basiliskamides biosynthetic gene cluster | Brevibacillus laterosporus PE36  Bacteria; Firmicutes; Bacilli; Bacillales; Paenibacillaceae;  Brevibacillus. | 32.7 | 0.64 | Identities = 25/101 (25%), Positives = 38/101 (38%), Gaps = 18/101 (18%)  Frame = +1 |
| Scaffold_C381 | 4911 | 5291 | 69 | hypothetical protein | universal stress protein [*Halogranum gelatinilyticum*] | 53% | 0.48 | 33% | gi\|1221564082\|WP_089699065.1 | 381 | BGC0000300\|c1\|8533-15621\|-\|SSMG_02536\|hypothetical_protein\|EFL06865.1 Length=2362 | Amychelin biosynthetic gene cluster | Streptomyces sp. AA4  Bacteria; Actinobacteria; Actinobacteridae; Actinomycetales;  Streptomycineae; Streptomycetaceae; Streptomyces. | 28.9 | 1.2 | Identities = 22/74 (30%), Positives = 35/74 (47%), Gaps = 1/74 (1%)  Frame = +1 |
| Scaffold_C381 | 6774 | 5644 | 70 | hypothetical protein | type I secretion C-terminal target domain (VC_A0849 subclass) [*Devosia lucknowensis*] | 55% | 0.006 | 26% | gi\|1191930390\|SMQ60375.1 | 1131 | BGC0000436\|c1\|6976-19461\|+\|PMI03_05101\|amino_acid_adenylation_enzyme/thioester_reductase_family_protein\|EJK79843.1 Length=4161 | Syringolin A biosynthetic gene cluster | Rhizobium sp. AP16  Bacteria; Proteobacteria; Alphaproteobacteria; Rhizobiales;  Rhizobiaceae; Rhizobium/Agrobacterium group; Rhizobium. | 30.4 | 3.1 | Identities = 13/29 (45%), Positives = 18/29 (62%), Gaps = 0/29 (0%)  Frame = +3 |
| Scaffold_C381 | 7181 | 6852 | 71 | hypothetical protein | AarF/ABC1/UbiB kinase family protein [*Salipaludibacillus aurantiacus*] | 86% | 0.067 | 27% | gi\|1225034216\|WP_093048293.1 | 330 | BGC0001045\|c1\|32106-36674\|+\|no_locus_tag\|polyketide_synthase_SpiC2\|AFR69335.1 Length=1522 | Spiruchostatin biosynthetic gene cluster | Pseudomonas sp. Q71576  Bacteria; Proteobacteria; Gammaproteobacteria; Pseudomonadales;  Pseudomonadaceae; Pseudomonas. | 27.7 | 2.3 | Identities = 16/53 (30%), Positives = 29/53 (55%), Gaps = 2/53 (4%)  Frame = +1 |
| Scaffold_C383 | 51 | 428 | 72 | hypothetical protein | PREDICTED: triadin-like isoform X3 [*Sinocyclocheilus rhinocerous*] | 100% | 0.044 | 28% | gi\|1025344283\|XP_016393021.1 | 378 | No hits found |  |  |  |  |  |
| Scaffold_C383 | 437 | 1396 | 73 | radical SAM domain protein | radical SAM/SPASM domain-containing protein [*Thermodesulfobium narugense*] | 83% | 2e-18 | 28% | gi\|503521935\|WP_013756246.1 | 960 | BGC0001209\|c1\|1089-2408\|-\|STER_1356\|Radical_SAM_superfamily_enzyme\|ABJ66529.1 Length=439 | Streptide biosynthetic gene cluster | Streptococcus thermophilus LMD-9  Bacteria; Firmicutes; Bacilli; Lactobacillales; Streptococcaceae;  Streptococcus. | 45.8 | 2.00E-05 | Identities = 59/264 (22%), Positives = 113/264 (43%), Gaps = 45/264 (17%)  Frame = +1 |
| Scaffold_C383 | 1471 | 1767 | 74 | hypothetical protein | hypothetical protein AKJ59_00445 [*candidate divison MSBL1 archaeon SCGC-AAA385M02*] | 87% | 2e-11 | 42% | gi\|985678815\|KXB08777.1 | 297 | BGC0000401\|c1\|5773-12882\|+\|no_locus_tag\|nonribosomal_peptide_synthetase_NRPS\|AEI70245.1 Length=2369 | Paenibactin biosynthetic gene cluster | Paenibacillus elgii B69  Bacteria; Firmicutes; Bacilli; Bacillales; Paenibacillaceae;  Paenibacillus. | 28.1 | 1.4 | Identities = 13/38 (34%), Positives = 17/38 (45%), Gaps = 0/38 (0%)  Frame = +3 |
| Scaffold_C383 | 1846 | 2127 | 75 | hypothetical protein | MULTISPECIES: glutaredoxin family protein [*Rhodococcus*] | 98% | 0.001 | 28% | gi\|1132145882\|WP_075833576.1 | 282 | BGC0001370\|c1\|16367-38248\|+\|no_locus_tag\|CDA_peptide_synthetase_I\|ALV82356.1 Length=7293 | Lipopeptide 8D1-1 / lipopeptide 8D1-2 biosynthetic gene cluster | Streptomyces rochei  Bacteria; Actinobacteria; Streptomycetales; Streptomycetaceae;  Streptomyces. | 28.1 | 0.91 | Identities = 15/24 (63%), Positives = 17/24 (71%), Gaps = 1/24 (4%)  Frame = +1 |
| Scaffold_C383 | 2338 | 2195 | 76 | hypothetical protein | fluoride efflux transporter CrcB [*SAR86 cluster bacterium*] | 97% | 1.3 | 37% | gi\|1247468918\|PCJ26075.1 | 144 | BGC0001178\|c1\|46730-48715\|+\|no_locus_tag\|ABC_transporter\|AGS77319.1 Length=661 | UK-68,597 biosynthetic gene cluster | Actinoplanes sp. ATCC 53533  Bacteria; Actinobacteria; Actinobacteridae; Actinomycetales;  Micromonosporineae; Micromonosporaceae; Actinoplanes. | 24.6 | 3.3 | Identities = 8/21 (38%), Positives = 15/21 (71%), Gaps = 0/21 (0%)  Frame = +1 |
| Scaffold_C383 | 2295 | 2441 | 77 | hypothetical protein | Glycosyl transferase family 2 [*Paenibacillus sp. UNC499MF*] | 93% | 7.0 | 37% | gi\|1090789188\|SEG25077.1 | 147 | BGC0000823\|c1\|890-2155\|-\|no_locus_tag\|N-glycosyl_transferase_RebG\|BAC15749.1 Length=421 | Rebeccamycin biosynthetic gene cluster | Lechevalieria aerocolonigenes  Bacteria; Actinobacteria; Actinobacteridae; Actinomycetales;  Pseudonocardineae; Pseudonocardiaceae; Lechevalieria. | 25 | 2.3 | Identities = 12/30 (40%), Positives = 17/30 (57%), Gaps = 1/30 (3%)  Frame = +1 |
| Scaffold_C383 | 2699 | 2502 | 78 | hypothetical protein | membrane protein [uncultured organism] | 60% | 1e-04 | 46% | gi\|452077110\|AGF93079.1 | 198 | BGC0001211\|c1\|2476-24696\|+\|AMYAL_RS0130210\|hypothetical_protein\|WP_020635019.1 Length=7406 | Albachelin biosynthetic gene cluster | mycolatopsis alba DSM 44262  Bacteria; Actinobacteria; Pseudonocardiales; Pseudonocardiaceae;  Amycolatopsis. | 28.5 | 0.3 | Identities = 18/41 (44%), Positives = 23/41 (56%), Gaps = 3/41 (7%)  Frame = -1 |
| Scaffold_C383 | 3825 | 2854 | 79 | hypothetical protein | hypothetical protein BA874_06665 [*Desulfuromonadales bacterium C00003068*] | 37% | 6e-11 | 34% | gi\|1072877864\|OEU76116.1 | 972 | BGC0000703\|c1\|172854-174539\|-\|no_locus_tag\|putative_ABC_transporter_solute_binding_lipoprotein\|BAE95570.1 Length=561 | Kanamycin biosynthetic gene cluster | Streptomyces kanamyceticus  Bacteria; Actinobacteria; Actinobacteridae; Actinomycetales;  Streptomycineae; Streptomycetaceae; Streptomyces. | 31.6 | 0.95 | Identities = 15/38 (39%), Positives = 21/38 (55%), Gaps = 2/38 (5%)  Frame = +1 |
| Scaffold_C383 | 4251 | 3985 | 80 | hypothetical protein | proline--tRNA ligase [*Chryseobacterium hungaricum*] | 88% | 0.77 | 31% | gi\|1221747315\|WP_089874552.1 | 267 | BGC0000120\|c1\|12080-14006\|+\|no_locus_tag\|carboxylesterase_family_protein\|AIG62138.1 Length=561 | Patulin biosynthetic gene cluster | Penicillium expansum  Eukaryota; Fungi; Dikarya; Ascomycota; Pezizomycotina;  Eurotiomycetes; Eurotiomycetidae; Eurotiales; Aspergillaceae;  Penicillium. | 25.4 | 7.3 | Identities = 13/23 (57%), Positives = 15/23 (65%), Gaps = 0/23 (0%)  Frame = -2 |
| Scaffold_C383 | 6279 | 4252 | 81 | hypothetical protein | hypothetical protein AKJ55_01585 [*candidate divison MSBL1 archaeon SCGC-AAA382M17*] | 82% | 2e-135 | 44% | gi\|985677923\|KXB07980.1 | 2028 | BGC0000556\|c1\|4932-6176\|+\|no_locus_tag\|SrtC\|BAB08164.1 Length=414 | Streptin biosynthetic gene cluster | Streptococcus pyogenes  Bacteria; Firmicutes; Bacilli; Lactobacillales; Streptococcaceae;  Streptococcus. | 36.2 | 0.073 | Identities = 26/81 (32%), Positives = 38/81 (47%), Gaps = 12/81 (15%)  Frame = +1 |
| Scaffold_C383 | 7178 | 6279 | 82 | hypothetical protein | hypothetical protein AKJ61_04790 [*candidate divison MSBL1 archaeon SCGC-AAA259B11*] | 51% | 6e-26 | 44% | gi\|985655732\|KXA88494.1 | 900 | BGC0000449\|c1\|4869-46985\|+\|no_locus_tag\|TriD\|AHF21228.1 Length=14038 | Tridecaptin biosynthetic gene cluster | Paenibacillus terrae  Bacteria; Firmicutes; Bacilli; Bacillales; Paenibacillaceae;  Paenibacillus. | 30.8 | 1.9 | Identities = 16/44 (36%), Positives = 24/44 (55%), Gaps = 1/44 (2%)  Frame = -1 |
| Scaffold_C383 | 7326 | 7168 | 83 | hypothetical protein | hypothetical protein AKJ65_01545 [*candidate divison MSBL1 archaeon SCGC-AAA259E19*] | 84% | 2e-04 | 41% | gi\|985663837\|KXA95556.1 | 159 | BGC0000954\|c1\|28650-35318\|+\|no_locus_tag\|polyketide_synthase\|CAQ18830.1 Length=2222 | Ajudazol biosynthetic gene cluster | Chondromyces crocatus  Bacteria; Proteobacteria; Deltaproteobacteria; Myxococcales;  Sorangiineae; Polyangiaceae; Chondromyces. | 26.6 | 0.9 | Identities = 12/27 (44%), Positives = 16/27 (59%), Gaps = 2/27 (7%)  Frame = +1 |
| Scaffold_C383 | 7729 | 7472 | 84 | hypothetical protein | hypothetical protein COS40_10735 [*Deltaproteobacteria bacterium CG03_land_8_20_14_0_80_45_14*] | 83% | 4e-11 | 45% | gi\|1277919625\|PIV20788.1 | 258 | BGC0000075\|c1\|60954-61580\|-\|no_locus_tag\|hypothetical_protein\|AFV30258.1 Length=208 | Hygrocin biosynthetic gene cluster | Streptomyces sp. LZ35  Bacteria; Actinobacteria; Actinobacteridae; Actinomycetales;  Streptomycineae; Streptomycetaceae; Streptomyces. | 28.5 | 0.53 | Identities = 18/70 (26%), Positives = 31/70 (44%), Gaps = 5/70 (7%)  Frame = +1 |
| Scaffold_C383 | 8048 | 7815 | 85 | hypothetical protein | PREDICTED: NADH dehydrogenase [ubiquinone] iron-sulfur protein 5-like [*Rhinopithecus roxellana*] | 90% | 1.1 | 34% | gi\|724805094\|XP_010386677.1 | 234 | BGC0001069\|c1\|76341-93941\|+\|no_locus_tag\|polyketide_synthase\|AAY89053.1 Length=5866 | Chivosazole biosynthetic gene cluster | Sorangium cellulosum  Bacteria; Proteobacteria; Deltaproteobacteria; Myxococcales;  Sorangiineae; Polyangiaceae; Sorangium. | 27.3 | 1.1 | Identities = 17/53 (32%), Positives = 22/53 (42%), Gaps = 3/53 (6%)  Frame = -3 |
| Scaffold_C383 | 8200 | 8045 | 86 | hypothetical protein | 30S ribosomal protein S27ae [*Candidatus Bathyarchaeota archaeon ex4484_218*] | 98% | 2.6 | 40% | gi\|1231993443\|OYT54120.1 | 156 | BGC0000047\|c1\|73062-74804\|+\|no_locus_tag\|putative_transcriptional_regulator\|ABB52549.1 Length=580 | Dihydrochalcomycin biosynthetic gene cluster | Streptomyces sp. KCTC 0041BP  Bacteria; Actinobacteria; Actinobacteridae; Actinomycetales;  Streptomycineae; Streptomycetaceae; Streptomyces. | 25 | 3 | Identities = 11/17 (65%), Positives = 12/17 (71%), Gaps = 0/17 (0%)  Frame = +3 |
| Scaffold_C383 | 8349 | 8197 | 87 | hypothetical protein | hypothetical protein AKJ38_00555 [*candidate divison MSBL1 archaeon SCGC-AAA259I14*] | 94% | 0.007 | 39% | gi\|985666320\|KXA97689.1 | 153 | BGC0000040\|c1\|91758-92081\|-\|no_locus_tag\|hypothetical_protein\|AAZ94404.1 Length=107 | Concanamycin A biosynthetic gene cluster | Streptomyces neyagawaensis  Bacteria; Actinobacteria; Actinobacteridae; Actinomycetales;  Streptomycineae; Streptomycetaceae; Streptomyces. | 25 | 1.9 | Identities = 16/45 (36%), Positives = 22/45 (49%), Gaps = 6/45 (13%)  Frame = +3 |
| Scaffold_C387 | 3 | 152 | 88 | hypothetical protein | type I-F CRISPR-associated protein Csy3 [*Vibrio natriegens*] | 91% | 3.9 | 33% | gi\|1042735364\|WP_065298158.1 | 150 | BGC0001304\|c1\|2602-8047\|-\|AFLA_108550\|polyketide_synthase,_putative\|EED53479.1 Length=1751 | Aflavarin biosynthetic gene cluster | Aspergillus flavus NRRL3357  Eukaryota; Fungi; Dikarya; Ascomycota; Pezizomycotina;  Eurotiomycetes; Eurotiomycetidae; Eurotiales; Aspergillaceae;  Aspergillus. | 27.3 | 0.46 | Identities = 16/40 (40%), Positives = 20/40 (50%), Gaps = 7/40 (18%)  Frame = +1 |
| Scaffold_C387 | 174 | 566 | 89 | Uncharacterized protein MJ0497 | nucleoid-structuring protein H-NS [*Haloplanus vescus]* | 96% | 3e-24 | 44% | gi\|1224615606\|WP_092635134.1 | 393 | BGC0000137\|c1\|90002-91573\|-\|Sare_1278\|FAD_dependent_oxidoreductase\|ABV97183.1 Length=523 | Rifamycin biosynthetic gene cluster | Salinispora arenicola CNS-205  Bacteria; Actinobacteria; Actinobacteridae; Actinomycetales;  Micromonosporineae; Micromonosporaceae; Salinispora. | 26.6 | 8.2 | Identities = 12/20 (60%), Positives = 13/20 (65%), Gaps = 0/20 (0%)  Frame = +1 |
| Scaffold_C387 | 1081 | 929 | 90 | hypothetical protein | hypothetical protein AKJ41_06430 [*candidate divison MSBL1 archaeon SCGC-AAA259O05*] | 96% | 6e-16 | 65% | gi\|985667374\|KXA98645.1 | 153 | BGC0001114\|c1\|22470-46580\|+\|no_locus_tag\|TstDEF\|AGN11881.1 Length=8036 | Thailanstatin biosynthetic gene cluster | Burkholderia thailandensis  Bacteria; Proteobacteria; Betaproteobacteria; Burkholderiales;  Burkholderiaceae; Burkholderia; pseudomallei group. | 27.7 | 0.26 | Identities = 12/31 (39%), Positives = 19/31 (61%), Gaps = 0/31 (0%)  Frame = -1 |
| Scaffold_C387 | 1334 | 1459 | 91 | hypothetical protein | zinc ribbon domain-containing protein [*Methanocaldococcus jannaschii*] | 68% | 2e-04 | 57% | gi\|499172328\|WP_010869915.1 | 126 | BGC0000457\|c1\|2048-6040\|-\|RHE_PF00457\|vicibactin_biosynthesis_non-ribosomal_peptide_synthase_protein\|ABC94347.1 Length=1330 | Vicibactin biosynthetic gene cluster | Rhizobium etli CFN 42  Bacteria; Proteobacteria; Alphaproteobacteria; Rhizobiales;  Rhizobiaceae; Rhizobium/Agrobacterium group; Rhizobium. | 24.6 | 2.6 | Identities = 8/24 (33%), Positives = 14/24 (58%), Gaps = 0/24 (0%)  Frame = +3 |
| Scaffold_C387 | 1882 | 2259 | 92 | hypothetical protein | hypothetical protein J07AB43_00980 [*Candidatus Nanosalina sp. J07AB43*] | 84% | 0.021 | 23% | gi\|339759081\|EGQ44332.1 | 378 | BGC0000957\|c1\|31887-40389\|+\|no_locus_tag\|PKS\|AHH25595.1 Length=2834 | Ansatrienin (mycotrienin) biosynthetic gene cluster | Streptomyces sp. XZQH13  Bacteria; Actinobacteria; Actinobacteridae; Actinomycetales;  Streptomycineae; Streptomycetaceae; Streptomyces. | 28.9 | 1.4 | Identities = 14/29 (48%), Positives = 18/29 (62%), Gaps = 5/29 (17%)  Frame = +1 |
| Scaffold_C387 | 2418 | 2606 | 93 | hypothetical protein | hypothetical protein [*Metallosphaera yellowstonensis*] | 95% | 0.001 | 39% | gi\|496362751\|WP_009071741.1 | 189 | BGC0000096\|c1\|15490-17466\|+\|ctg1_orf15\|\|ctg1_orf15 Length=658 | Midecamycin biosynthetic gene cluster | Streptomyces mycarofaciens  Bacteria; Actinobacteria; Actinobacteridae; Actinomycetales;  Streptomycineae; Streptomycetaceae; Streptomyces. | 27.7 | 0.47 | Identities = 17/56 (30%), Positives = 27/56 (48%), Gaps = 4/56 (7%)  Frame = -2 |
| Scaffold_C387 | 2613 | 2924 | 94 | hypothetical protein | hypothetical protein [*Methylobacter tundripaludum*] | 86% | 7.5 | 33% | gi\|493945967\|WP_006889921.1 | 312 | BGC0001186\|c1\|33050-60031\|+\|no_locus_tag\|malonyl_CoA-acyl_carrier_protein_transacylase\|AKQ22698.1 Length=8993 | Misakinolide biosynthetic gene cluster | Candidatus Entotheonella sp. (ex. Theonella swinhoei)  Bacteria; Proteobacteria; Deltaproteobacteria; Candidatus  Entotheonella. | 26.6 | 5 | Identities = 11/33 (33%), Positives = 15/33 (45%), Gaps = 0/33 (0%)  Frame = -1 |
| Scaffold_C387 | 3247 | 3438 | 95 | hypothetical protein | type III restriction endonuclease subunit R [*Thermodesulfovibrio yellowstonii*] | 65% | 1.5 | 39% | gi\|501539858\|WP_012545345.1 | 192 | BGC0000655\|c1\|36030-37805\|+\|no_locus_tag\|SioT\|ACN80657.1 Length=591 | Siomycin biosynthetic gene cluster | Streptomyces sioyaensis  Bacteria; Actinobacteria; Actinobacteridae; Actinomycetales;  Streptomycineae; Streptomycetaceae; Streptomyces. | 29.3 | 0.13 | Identities = 11/31 (35%), Positives = 18/31 (58%), Gaps = 0/31 (0%)  Frame = +1 |
| Scaffold_C387 | 3495 | 3704 | 96 | hypothetical protein | MarR family transcriptional regulator [*Cystobacter fuscus*] | 89% | 2e-05 | 37% | gi\|1243263867\|WP_095985690.1 | 210 | BGC0001065\|c1\|135060-135515\|+\|sccontig008-70\|Marr_family_transcriptional_regulator\|AEZ64578.1 Length=151 | Herboxidiene biosynthetic gene cluster | Streptomyces chromofuscus  Bacteria; Actinobacteria; Actinobacteridae; Actinomycetales;  Streptomycineae; Streptomycetaceae; Streptomyces. | 36.2 | 5.00E-04 | Identities = 20/62 (32%), Positives = 32/62 (52%), Gaps = 1/62 (2%)  Frame = +1 |
| Scaffold_C387 | 3713 | 4636 | 97 | hypothetical protein | tyrosine recombinase XerS [*Thalassobius aestuarii*] | 70% | 1e-05 | 26% | gi\|1225080260\|WP_093093705.1 | 924 | BGC0000093\|c1\|2432-3331\|+\|no_locus_tag\|LysR-family_transcriptional_regulator\|ADC45513.1 Length=299 | Meilingmycin biosynthetic gene cluster | Streptomyces nanchangensis  Bacteria; Actinobacteria; Actinobacteridae; Actinomycetales;  Streptomycineae; Streptomycetaceae; Streptomyces. | 30.4 | 1.8 | Identities = 14/33 (42%), Positives = 19/33 (58%), Gaps = 0/33 (0%)  Frame = +3 |
| Scaffold_C387 | 4656 | 5072 | 98 | hypothetical protein | phage tail tape measure protein [*Bradyrhizobium yuanmingense*] | 91% | 0.71 | 27% | gi\|916256801\|WP_050991882.1 | 417 | BGC0001342\|c1\|17602-36369\|+\|no_locus_tag\|TugA\|ADH04657.1 Length=6255 | Thuggacin biosynthetic gene cluster | Chondromyces crocatus  Bacteria; Proteobacteria; Deltaproteobacteria; Myxococcales;  Sorangiineae; Polyangiaceae; Chondromyces. | 31.2 | 0.26 | Identities = 19/60 (32%), Positives = 31/60 (52%), Gaps = 4/60 (7%)  Frame = -2 |
| Scaffold_C387 | 5124 | 6224 | 99 | Glycosyltransferase | glycogen synthase GlgA [*Thermococcus barophilus*] | 96% | 2e-60 | 36% | gi\|948744483\|WP_056934594.1 | 1101 | BGC0000808\|c2\|4303-6768\|-\|Bmul_4608\|glycosyl_transferase_group_1\|ABX18286.1 Length=821 | Cepacian biosynthetic gene cluster | Burkholderia multivorans ATCC 17616  Bacteria; Proteobacteria; Betaproteobacteria; Burkholderiales;  Burkholderiaceae; Burkholderia; Burkholderia cepacia complex. | 111 | 1.00E-26 | Identities = 110/382 (29%), Positives = 181/382 (47%), Gaps = 32/382 (8%)  Frame = +1 |
| Scaffold_C387 | 6214 | 6456 | 100 | hypothetical protein | hypothetical protein [*Anoxybacillus sp. UARK-01*] | 83% | 5.2 | 33% | gi\|1172488465\|WP_080860765.1 | 243 | BGC0000452\|c1\|22858-42321\|+\|BBR47_27890\|tyrocidine_synthetase_III\|BAH43766.1 Length=6487 | Tyrocidine biosynthetic gene cluster | Brevibacillus brevis NBRC 100599  Bacteria; Firmicutes; Bacilli; Bacillales; Paenibacillaceae;  Brevibacillus. | 27.7 | 0.87 | Identities = 11/24 (46%), Positives = 14/24 (58%), Gaps = 0/24 (0%)  Frame = +1 |
| Scaffold_C387 | 6590 | 6453 | 101 | hypothetical protein | chromosome segregation protein SMC [*Xanthomonas sp. NCPPB1128*] | 66% | 2.4 | 43% | gi\|868610415\|WP_048492025.1 | 138 | BGC0001283\|c1\|18329-18730\|-\|SLI_RS05070\|transcriptional_regulator\|WP_016325505.1 Length=133 | Arsenopolyketides biosynthetic gene cluster | Streptomyces lividans 1326  Bacteria; Actinobacteria; Streptomycetales; Streptomycetaceae;  Streptomyces. | 25.8 | 1.2 | Identities = 9/25 (36%), Positives = 14/25 (56%), Gaps = 0/25 (0%)  Frame = +1 |
| Scaffold_C387 | 6627 | 6884 | 102 | hypothetical protein | demethylmenaquinone methyltransferase [*Thermococcus barophilus*] | 100% | 9e-25 | 52% | gi\|948744395\|WP_056934592.1 | 258 | BGC0000222\|c1\|25743-26438\|+\|no_locus_tag\|putative_AHL-lactonase\|BAJ52695.1 Length=231 | FD-594 biosynthetic gene cluster | Streptomyces sp. TA-0256  Bacteria; Actinobacteria; Actinobacteridae; Actinomycetales;  Streptomycineae; Streptomycetaceae; Streptomyces. | 27.3 | 1.1 | Identities = 17/42 (40%), Positives = 21/42 (50%), Gaps = 0/42 (0%)  Frame = +1 |
| Scaffold_C387 | 7066 | 7926 | 103 | hypothetical protein | Type I phosphodiesterase / nucleotide pyrophosphatase [*Candidatus Bathyarchaeota archaeon B26-1*] | 99% | 2e-16 | 25% | gi\|1007514390\|KYH42239.1 | 861 | BGC0000335\|c1\|19123-26763\|+\|no_locus_tag\|nonribosomal_peptide_synthetase\|AID65224.1 Length=2546 | Cystomanamides biosynthetic gene cluster | Cystobacter fuscus  Bacteria; Proteobacteria; Deltaproteobacteria; Myxococcales;  Cystobacterineae; Cystobacteraceae; Cystobacter. | 32.3 | 0.57 | Identities = 35/99 (35%), Positives = 44/99 (44%), Gaps = 9/99 (9%)  Frame = -1 |
| Scaffold_C387 | 7923 | 8429 | 104 | hypothetical protein | hypothetical protein AKJ50_02060 [*candidate divison MSBL1 archaeon SCGC-AAA382A13*] | 96% | 1e-33 | 40% | gi\|985674229\|KXB04721.1 | 507 | BGC0001247\|c7\|1400-2800\|+\|no_locus_tag\|oxidoreductase\|ABK64184.1 Length=450 | Cercosporin biosynthetic gene cluster | Cercospora nicotianae  Eukaryota; Fungi; Dikarya; Ascomycota; Pezizomycotina;  Dothideomycetes; Dothideomycetidae; Capnodiales; Mycosphaerellaceae;  Cercospora. | 30.4 | 0.75 | Identities = 12/35 (34%), Positives = 23/35 (66%), Gaps = 0/35 (0%)  Frame = +3 |
| Scaffold_C387 | 9249 | 8563 | 105 | hypothetical protein | hypothetical protein AKJ44_00660 [*candidate divison MSBL1 archaeon SCGC-AAA261F17*] | 67% | 7e-16 | 35% | gi\|985671597\|KXB02393.1 | 687 | BGC0000845\|c1\|18174-19505\|-\|SSCG_00163\|dipeptide-binding_lipoprotein\|EDY47135.1 Length=443 | Clavulanic acid biosynthetic gene cluster | Streptomyces clavuligerus ATCC 27064  Bacteria; Actinobacteria; Actinobacteridae; Actinomycetales;  Streptomycineae; Streptomycetaceae; Streptomyces. | 28.5 | 4.9 | Identities = 27/84 (32%), Positives = 39/84 (46%), Gaps = 5/84 (6%)  Frame = -1 |
| Scaffold_C387 | 9386 | 10147 | 106 | hypothetical protein | hypothetical protein AKJ66_01500 [*candidate divison MSBL1 archaeon SCGC-AAA259E22*] | 100% | 1e-110 | 61% | gi\|985661672\|KXA93659.1 | 762 | BGC0000398\|c1\|11026-24123\|+\|no_locus_tag\|nonribosomal_peptide_synthetase\|AFH75329.1 Length=4365 | Orfamide biosynthetic gene cluster | Pseudomonas sp. CMR12a  Bacteria; Proteobacteria; Gammaproteobacteria; Pseudomonadales;  Pseudomonadaceae; Pseudomonas. | 35.8 | 0.034 | Identities = 18/49 (37%), Positives = 27/49 (55%), Gaps = 0/49 (0%)  Frame = -2 |
| Scaffold_1 | 343 | 140 | 107 | hypothetical protein | zinc finger protein ZAT9-like [*Aegilops tauschii subsp. tauschii*] | 91% | 0.004 | 33% | gi\|1149713660\|XP_020182857.1 | 204 | BGC0001069\|c1\|76341-93941\|+\|no_locus_tag\|polyketide_synthase\|AAY89053.1 Length=5866 | Chivosazole biosynthetic gene cluster | Sorangium cellulosum  Bacteria; Proteobacteria; Deltaproteobacteria; Myxococcales;  Sorangiineae; Polyangiaceae; Sorangium. | 28.5 | 0.28 | Identities = 13/29 (45%), Positives = 18/29 (62%), Gaps = 0/29 (0%)  Frame = -3 |
| scaffold_1 | 811 | 368 | 108 | hypothetical protein | PREDICTED: retrotransposon-like protein 1 [*Mandrillus leucophaeus*] | 82% | 0.16 | 26% | gi\|795152535\|XP_011838852.1 | 444 | BGC0000627\|c1\|5977-6496\|-\|AFLA_094970\|hypothetical_protein\|EED49416.1 Length=93 | Ustiloxin B biosynthetic gene cluster | Aspergillus flavus NRRL3357  Eukaryota; Fungi; Dikarya; Ascomycota; Pezizomycotina;  Eurotiomycetes; Eurotiomycetidae; Eurotiales; Aspergillaceae;  Aspergillus. | 28.1 | 1.1 | Identities = 12/26 (46%), Positives = 18/26 (69%), Gaps = 0/26 (0%)  Frame = +2 |
| scaffold_1 | 973 | 845 | 109 | hypothetical protein | MULTISPECIES: EamA/RhaT family transporter [*Pseudomonas*] | 92% | 2.9 | 44% | gi\|985599342\|WP_060838839.1 | 129 | BGC0000128\|c1\|27715-29157\|+\|no_locus_tag\|outer_membrane_channel_protein\|AAY59070.1 Length=480 | Pyoluteorin biosynthetic gene cluster | Pseudomonas sp. M18  Bacteria; Proteobacteria; Gammaproteobacteria; Pseudomonadales;  Pseudomonadaceae; Pseudomonas. | 25 | 1.8 | Identities = 11/14 (79%), Positives = 11/14 (79%), Gaps = 0/14 (0%)  Frame = +3 |
| scaffold_1 | 1928 | 1800 | 110 | hypothetical protein | Holliday junction DNA helicase [*Methanobacterium paludis*] | 89% | 0.065 | 37% | gi\|503592544\|WP_013826620.1 | 141 | BGC0000093\|c1\|78606-96074\|-\|no_locus_tag\|modular_polyketide_synthase\|ADC45538.1 Length=5822 | Meilingmycin biosynthetic gene cluster | Streptomyces nanchangensis  Bacteria; Actinobacteria; Actinobacteridae; Actinomycetales;  Streptomycineae; Streptomycetaceae; Streptomyces. | 25 | 2.5 | Identities = 10/23 (43%), Positives = 15/23 (65%), Gaps = 0/23 (0%)  Frame = -1 |
| scaffold_1 | 2143 | 1928 | 111 | hypothetical protein | hypothetical protein AKJ41_06430 [*candidate divison MSBL1 archaeon SCGC-AAA259O05*] | 77% | 1e-16 | 65% | gi\|985667374\|KXA98645.1 | 192 | BGC0000039\|c1\|18389-26483\|-\|no_locus_tag\|polyketide_synthase\|BAC20566.1 Length=2563 | Compactin biosynthetic gene cluster | Penicillium citrinum  Eukaryota; Fungi; Dikarya; Ascomycota; Pezizomycotina;  Eurotiomycetes; Eurotiomycetidae; Eurotiales; Aspergillaceae;  Penicillium. | 27.7 | 0.6 | Identities = 15/58 (26%), Positives = 24/58 (41%), Gaps = 6/58 (10%)  Frame = +1 |
| scaffold_1 | 2777 | 2475 | 112 | hypothetical protein | titin-like isoform X11 [*Leptinotarsa decemlineata*] | 87% | 0.46 | 24% | gi\|1285010384\|XP_023017183.1 | 747 | BGC0000209\|c1\|1986-2753\|+\|no_locus_tag\|Ctc7\|AEI98639.1 Length=255 | Chlortetracycline biosynthetic gene cluster | Streptomyces aureofaciens  Bacteria; Actinobacteria; Actinobacteridae; Actinomycetales;  Streptomycineae; Streptomycetaceae; Streptomyces. | 31.2 | 0.54 | Identities = 16/35 (46%), Positives = 22/35 (63%), Gaps = 2/35 (6%)  Frame = +1 |
| scaffold_1 | 3933 | 3292 | 113 | hypothetical protein | hypothetical protein [*Lachnospiraceae bacterium A4*] | 68% | 5.3 | 29% | gi\|511028138\|WP_016282353.1 | 330 | BGC0001405\|c1\|4-2698\|-\|CHGG_07633\|hypothetical_protein\|EAQ86380.1 Length=880 | Chaetoviridin / chaetomugilin biosynthetic gene cluster | Chaetomium globosum CBS 148.51  Eukaryota; Fungi; Dikarya; Ascomycota; Pezizomycotina;  Sordariomycetes; Sordariomycetidae; Sordariales; Chaetomiaceae;  Chaetomium. | 27.7 | 2 | Identities = 14/43 (33%), Positives = 24/43 (56%), Gaps = 1/43 (2%)  Frame = +1 |
| scaffold_1 | 4255 | 3992 | 114 | hypothetical protein | peptidase M16 [*Lachnospiraceae bacterium MC2017*] | 59% | 1.1 | 35% | gi\|769123853\|WP_044906012.1 | 267 | BGC0001060\|c1\|2116-3345\|+\|no_locus_tag\|hypothetical_protein\|ACB47031.1 Length=409 | Dynemicin biosynthetic gene cluster | Micromonospora chersina  Bacteria; Actinobacteria; Actinobacteridae; Actinomycetales;  Micromonosporineae; Micromonosporaceae; Micromonospora. | 26.2 | 4.2 | Identities = 12/28 (43%), Positives = 15/28 (54%), Gaps = 0/28 (0%)  Frame = -1 |
| scaffold_1 | 4952 | 4728 | 115 | hypothetical protein | PREDICTED: PDZ domain-containing protein 11-like [*Branchiostoma belcheri*] | 55% | 1.0 | 26% | gi\|1126178638\|XP_019621686.1 | 702 | BGC0000189\|c1\|4110-5090\|-\|XBJ1_3898\|3-oxoacyl-[acyl-carrier-protein]_synthase_III\|CBJ83016.1 Length=326 | Xenocyloins biosynthetic gene cluster | Xenorhabdus bovienii SS-2004  Bacteria; Proteobacteria; Gammaproteobacteria; Enterobacteriales;  Enterobacteriaceae; Xenorhabdus. | 30.8 | 0.96 | Identities = 17/47 (36%), Positives = 24/47 (51%), Gaps = 2/47 (4%)  Frame = +1 |
| scaffold_1 | 5174 | 4959 | 116 | hypothetical protein | hypothetical protein C445_03778 [*Halobiforma lacisalsi AJ5*] | 52% | 1e-10 | 63% | gi\|445785150\|EMA35945.1 | 219 | BGC0001381\|c1\|154819-157857\|+\|no_locus_tag\|alpha-Mannosidase\|AJO72751.1 Length=1012 | Brasilinolide biosynthetic gene cluster | Nocardia terpenica  Bacteria; Actinobacteria; Corynebacteriales; Nocardiaceae; Nocardia. | 26.2 | 2.4 | Identities = 14/41 (34%), Positives = 22/41 (54%), Gaps = 1/41 (2%)  Frame = +1 |
| scaffold_1 | 5985 | 6125 | 117 | hypothetical protein | hypothetical protein [*Thermococcus sp. AM4]* | 85% | 2.1 | 29% | gi\|503888403\|WP_014122397.1 | 171 | BGC0000455\|c1\|54431-55618\|+\|no_locus_tag\|putative_antiporter\|AEI58883.1 Length=395 | Vancomycin biosynthetic gene cluster | Amycolatopsis orientalis HCCB10007  Bacteria; Actinobacteria; Actinobacteridae; Actinomycetales;  Pseudonocardineae; Pseudonocardiaceae; Amycolatopsis. | 26.2 | 1.3 | Identities = 10/24 (42%), Positives = 16/24 (67%), Gaps = 0/24 (0%)  Frame = +3 |
| scaffold_1 | 6601 | 6410 | 118 | hypothetical protein | Lactamase_B domain containing protein [*Magnaporthe oryzae Y34*] | 90% | 0.59 | 35% | gi\|440474699\|ELQ43427.1 | 195 | BGC0000038\|c1\|53146-54738\|+\|no_locus_tag\|putative_decarboxylase\|CAC37885.1 Length=530 | Coelimycin biosynthetic gene cluster | Streptomyces coelicolor A3(2)  Bacteria; Actinobacteria; Actinobacteridae; Actinomycetales;  Streptomycineae; Streptomycetaceae; Streptomyces; Streptomyces  albidoflavus group. | 24.3 | 8.9 | Identities = 12/29 (41%), Positives = 16/29 (55%), Gaps = 0/29 (0%)  Frame = +1 |
| scaffold_1 | 6857 | 7603 | 119 | hypothetical protein | Phage integrase [*Pseudomonas plecoglossicida*] | 86% | 8e-06 | 23% | gi\|752309407\|AJG12016.1 | 774 | BGC0000164\|c1\|64292-82183\|+\|no_locus_tag\|type_I_polyketide_synthase\|BAE93731.1 Length=5963 | Tetronomycin biosynthetic gene cluster | Streptomyces sp. NRRL 11266  Bacteria; Actinobacteria; Actinobacteridae; Actinomycetales;  Streptomycineae; Streptomycetaceae; Streptomyces. | 29.3 | 4.3 | Identities = 15/35 (43%), Positives = 23/35 (66%), Gaps = 4/35 (11%)  Frame = -1 |
| scaffold_1 | 7575 | 7904 | 120 | hypothetical protein | hypothetical protein AKJ45_03820 [*candidate divison MSBL1 archaeon SCGC-AAA261F19*] | 95% | 6e-05 | 43% | gi\|985671108\|KXB01950.1 | 129 | BGC0001365\|c1\|8582-9413\|+\|CPUR_02676\|uncharacterized_protein\|CCE28985.1 Length=249 | Clapurines biosynthetic gene cluster | Claviceps purpurea 20.1  Eukaryota; Fungi; Dikarya; Ascomycota; Pezizomycotina;  Sordariomycetes; Hypocreomycetidae; Hypocreales; Clavicipitaceae;  Claviceps. | 25.4 | 1.4 | Identities = 11/30 (37%), Positives = 16/30 (53%), Gaps = 0/30 (0%)  Frame = +1 |
| scaffold_1 | 7974 | 8240 | 121 | hypothetical protein | MULTISPECIES: sulfite exporter TauE/SafE family protein [*Rhodococcus*] | 93% | 2.2 | 29% | gi\|1233996907\|WP_094639404.1 | 393 | BGC0000098\|c1\|1535-9615\|-\|no_locus_tag\|polyketide_synthase\|ABA02240.1 Length=2547 | Monacolin K biosynthetic gene cluster | Monascus pilosus  Eukaryota; Fungi; Dikarya; Ascomycota; Pezizomycotina;  Eurotiomycetes; Eurotiomycetidae; Eurotiales; Aspergillaceae;  Monascus. | 32 | 0.14 | Identities = 27/90 (30%), Positives = 45/90 (50%), Gaps = 12/90 (13%)  Frame = -2 |
| scaffold_1 | 8580 | 9281 | 122 | hypothetical protein | glycosyl transferase [*Candidatus Altiarchaeales archaeon ex4484_96*] | 70% | 3e-18 | 33% | gi\|1231964345\|OYT27700.1 | 966 | BGC0000776\|c1\|8542-9525\|+\|no_locus_tag\|beta-1,3-glucosyltransferase\|CAD19788.1 Length=327 | Lipopolysaccharide biosynthetic gene cluster | Escherichia coli  Bacteria; Proteobacteria; Gammaproteobacteria; Enterobacteriales;  Enterobacteriaceae; Escherichia. | 53.1 | 8.00E-08 | Identities = 25/99 (25%), Positives = 50/99 (51%), Gaps = 9/99 (9%)  Frame = +1 |
| scaffold_1 | 9711 | 9929 | 123 | hypothetical protein | hypothetical protein AKJ65_07070 [*candidate divison MSBL1 archaeon SCGC-AAA259E19*] | 98% | 2e-59 | 41% | gi\|985660686\|KXA92773.1 | 831 | BGC0001381\|c1\|47333-60052\|+\|no_locus_tag\|Type_I_modular_polyketide_synthase\|AJO72734.1 Length=4239 | Brasilinolide biosynthetic gene cluster | Nocardia terpenica  Bacteria; Actinobacteria; Corynebacteriales; Nocardiaceae; Nocardia. | 32.3 | 0.51 | Identities = 25/98 (26%), Positives = 41/98 (42%), Gaps = 11/98 (11%)  Frame = -1 |
| scaffold_1 | 10070 | 9900 | 124 | hypothetical protein | hypothetical protein A3D62_00230 [*Candidatus Kaiserbacteria bacterium RIFCSPHIGHO2_02_FULL_49_11*] | 79% | 2e-20 | 40% | gi\|1083558182\|OGG55055.1 | 489 | BGC0001077\|c1\|2646-3353\|-\|AMIS_77580\|3-alkyl-2-hydroxyresorcinol_methyltransferase\|BAL92978.1 Length=235 | Alkyl-O-Dihydrogeranyl-Methoxyhydroquinones biosynthetic gene  cluster | Actinoplanes missouriensis 431  Bacteria; Actinobacteria; Actinobacteridae; Actinomycetales;  Micromonosporineae; Micromonosporaceae; Actinoplanes. | 42 | 7.00E-05 | Identities = 18/44 (41%), Positives = 29/44 (66%), Gaps = 0/44 (0%)  Frame = +1 |
| scaffold_1 | 10238 | 10432 | 125 | hypothetical protein | hypothetical protein AKJ44_00660 [*candidate divison MSBL1 archaeon SCGC-AAA261F17*] | 67% | 3e-14 | 34% | gi\|985671597\|KXB02393.1 | 684 | BGC0001030\|c1\|3246-5384\|-\|no_locus_tag\|ochratoxin_A_non-ribosomal_peptide_synthetase\|AAS98174.1 Length=712 | Ochratoxin A biosynthetic gene cluster | Penicillium nordicum  Eukaryota; Fungi; Dikarya; Ascomycota; Pezizomycotina;  Eurotiomycetes; Eurotiomycetidae; Eurotiales; Aspergillaceae;  Penicillium. | 31.6 | 0.57 | Identities = 18/52 (35%), Positives = 25/52 (48%), Gaps = 3/52 (6%)  Frame = -1 |
| scaffold_1 | 10642 | 11415 | 126 | hypothetical protein | No significant similarity found. |  |  |  |  | 129 | BGC0000242\|c1\|5269-6654\|+\|no_locus_tag\|putative_FAD-depending_monooxygenase\|CAM34336.1 Length=461 | Lysolipin biosynthetic gene cluster | Streptomyces tendae  Bacteria; Actinobacteria; Actinobacteridae; Actinomycetales;  Streptomycineae; Streptomycetaceae; Streptomyces. | 26.9 | 0.38 | Identities = 10/18 (56%), Positives = 14/18 (78%), Gaps = 0/18 (0%)  Frame = +1 |
| scaffold_1 | 11429 | 11557 | 127 | hypothetical protein | MULTISPECIES: Crp/Fnr family transcriptional regulator [*Methylobacterium*] | 95% | 6.7 | 32% | gi\|985605765\|WP_060845260.1 | 216 | BGC0000360\|c1\|36242-37996\|-\|no_locus_tag\|Halogenase\|CBL93722.1 Length=584 | GE81112 biosynthetic gene cluster | Streptomyces sp. L-49973  Bacteria; Actinobacteria; Actinobacteridae; Actinomycetales;  Streptomycineae; Streptomycetaceae; Streptomyces. | 28.1 | 0.56 | Identities = 15/41 (37%), Positives = 21/41 (51%), Gaps = 0/41 (0%)  Frame = -1 |
| scaffold_1 | 11575 | 11967 | 128 | hypothetical protein | hypothetical protein [*Haloterrigena daqingensis*] | 97% | 5e-06 | 36% | gi\|1134344576\|WP_076584096.1 | 303 | BGC0000077\|c1\|6151-12661\|-\|no_locus_tag\|non-reducing_polyketide_synthase\|ACD39762.1 Length=2049 | Hypothemycin biosynthetic gene cluster | Hypomyces subiculosus  Eukaryota; Fungi; Dikarya; Ascomycota; Pezizomycotina;  Sordariomycetes; Hypocreomycetidae; Hypocreales; Hypocreaceae;  Hypomyces. | 27.7 | 1.6 | Identities = 12/37 (32%), Positives = 21/37 (57%), Gaps = 0/37 (0%)  Frame = +1 |
| scaffold_1 | 12118 | 13083 | 129 | Glycosyltransferase | hypothetical protein [*Halioglobus sp. HI00S01*] | 61% | 4e-04 | 26% | gi\|1053761548\|WP_066054323.1 | 642 | BGC0000266\|c1\|29882-31063\|+\|no_locus_tag\|DNA_methylase\|AAM97371.1 Length=393 | Rubromycin biosynthetic gene cluster | Streptomyces collinus  Bacteria; Actinobacteria; Actinobacteridae; Actinomycetales;  Streptomycineae; Streptomycetaceae; Streptomyces. | 29.6 | 1.6 | Identities = 22/79 (28%), Positives = 36/79 (46%), Gaps = 17/79 (22%)  Frame = -1 |
| scaffold_1 | 13088 | 13918 | 130 | hypothetical protein | hypothetical protein [*halophilic archaeon DL31*] | 93% | 0.004 | 28% | gi\|503819043\|WP_014053037.1 | 264 | BGC0001385\|c1\|14721-15503\|-\|no_locus_tag\|ketosteroid_isomerase-like_protein\|ANF07282.1 Length=242 | Byssochlamic acid / agnestadrides biosynthetic gene cluster | Byssochlamys fulva  Eukaryota; Fungi; Dikarya; Ascomycota; Pezizomycotina;  Eurotiomycetes; Eurotiomycetidae; Eurotiales; Thermoascaceae;  Byssochlamys. | 30.4 | 0.12 | Identities = 17/50 (34%), Positives = 26/50 (52%), Gaps = 2/50 (4%)  Frame = +1 |
| scaffold_1 | 13908 | 14396 | 131 | hypothetical protein | endonuclease MutS2 [*Thermoanaerobacter kivui*] | 100% | 0.099 | 31% | gi\|902966403\|WP_049685449.1 | 225 | BGC0000270\|c1\|32084-32932\|+\|no_locus_tag\|endonuclease/N-glycosylase\|AAL15611.1 Length=282 | Simocyclinone biosynthetic gene cluster | Streptomyces antibioticus  Bacteria; Actinobacteria; Actinobacteridae; Actinomycetales;  Streptomycineae; Streptomycetaceae; Streptomyces. | 26.2 | 2.7 | Identities = 17/49 (35%), Positives = 23/49 (47%), Gaps = 7/49 (14%)  Frame = -3 |
| scaffold_1 | 15225 | 14542 | 132 | hypothetical protein | Zinc finger CCCH domain-containing protein 13 [*Ophiophagus hannah*] | 66% | 0.008 | 47% | gi\|565306998\|ETE61454.1 | 216 | No hits found |  |  |  |  |  |
| scaffold_10 | 347 | 33 | 133 | hypothetical protein | unknown [*Firmicutes bacterium CAG:822*] | 99% | 1.2 | 28% | gi\|524066053\|CCY46597.1 | 315 | BGC0000339\|c1\|19078-28485\|+\|no_locus_tag\|putative_non-ribosomal_peptide_synthetase\|BAE98156.1 Length=3135 | Echinomycin biosynthetic gene cluster | Streptomyces lasaliensis  Bacteria; Actinobacteria; Actinobacteridae; Actinomycetales;  Streptomycineae; Streptomycetaceae; Streptomyces. | 28.1 | 1.3 | Identities = 23/67 (34%), Positives = 33/67 (49%), Gaps = 8/67 (12%)  Frame = +3 |
| scaffold_10 | 786 | 646 | 134 | hypothetical protein | hypothetical protein [*Streptomyces sp. CT34*] | 60% | 9.3 | 43% | gi\|759545095\|WP_043264986.1 | 141 | BGC0001358\|c1\|63670-99575\|+\|VFPBJ_02539\|nonribosomal_peptide_synthase\|OAQ83772.1 Length=11872 | Leucinostatins biosynthetic gene cluster | Purpureocillium lilacinum  Eukaryota; Fungi; Dikarya; Ascomycota; Pezizomycotina;  Sordariomycetes; Hypocreomycetidae; Hypocreales;  Ophiocordycipitaceae; Purpureocillium. | 25 | 2.5 | Identities = 10/17 (59%), Positives = 12/17 (71%), Gaps = 0/17 (0%)  Frame = -1 |
| scaffold_10 | 952 | 1104 | 135 | hypothetical protein | AbrB family transcriptional regulator [*Chloroflexi bacterium RBG_16_56_11*] | 96% | 3.0 | 33% | gi\|1084576458\|OGO32659.1 | 153 | BGC0001096\|c1\|80014-80904\|+\|no_locus_tag\|malonyl_CoA-acyl_carrier_protein_transacylase\|ADH01491.1 Length=296 | FR901464 biosynthetic gene cluster | Pseudomonas sp. 2663  Bacteria; Proteobacteria; Gammaproteobacteria; Pseudomonadales;  Pseudomonadaceae; Pseudomonas. | 27.3 | 0.43 | Identities = 14/28 (50%), Positives = 19/28 (68%), Gaps = 1/28 (4%)  Frame = +1 |
| scaffold_12 | 818 | 636 | 136 | hypothetical protein | HD family phosphohydrolase [*Desulfovibrio vulgaris*] | 66% | 0.94 | 43% | gi\|499240731\|WP_010938271.1 | 183 | BGC0000179\|c1\|3178-18618\|+\|sce3188\|Polyketide_synthase\|CAN93347.1 Length=5146 | Etnangien biosynthetic gene cluster | Sorangium cellulosum So ce56  Bacteria; Proteobacteria; Deltaproteobacteria; Myxococcales;  Sorangiineae; Polyangiaceae; Sorangium. | 27.3 | 0.68 | Identities = 15/42 (36%), Positives = 19/42 (45%), Gaps = 0/42 (0%)  Frame = +1 |
| scaffold_12 | 1281 | 1153 | 137 | hypothetical protein | MULTISPECIES: hypothetical protein [*Methanoculleus*] | 92% | 0.008 | 38% | gi\|1011349207\|WP_062263082.1 | 129 | BGC0001370\|c1\|12463-13788\|-\|no_locus_tag\|putative_aminotransferase\|ALV82375.1 Length=441 | Lipopeptide 8D1-1 / lipopeptide 8D1-2 biosynthetic gene cluster | Streptomyces rochei  Bacteria; Actinobacteria; Streptomycetales; Streptomycetaceae;  Streptomyces. | 25.8 | 0.92 | Identities = 10/20 (50%), Positives = 13/20 (65%), Gaps = 0/20 (0%)  Frame = +1 |
| scaffold_13 | 74 | 373 | 138 | hypothetical protein | alpha/beta hydrolase [*Clostridium sp. CAG:590*] | 92% | 0.019 | 30% | gi\|524004773\|CCX87972.1 | 300 | BGC0000012\|c1\|11261-19092\|+\|no_locus_tag\|PKSN_polyketide_synthase_for_alternapyrone_biosynthesis\|BAD83684.1 Length=2551 | Alternapyrone biosynthetic gene cluster | Alternaria solani  Eukaryota; Fungi; Dikarya; Ascomycota; Pezizomycotina;  Dothideomycetes; Pleosporomycetidae; Pleosporales; Pleosporineae;  Pleosporaceae; Alternaria. | 29.3 | 0.46 | Identities = 11/39 (28%), Positives = 25/39 (64%), Gaps = 3/39 (8%)  Frame = +1 |
| scaffold_13 | 1347 | 1562 | 139 | hypothetical protein | hypothetical protein AKJ51_00535 [*candidate divison MSBL1 archaeon SCGC-AAA382A20*] | 97% | 0.15 | 29% | gi\|985677542\|KXB07666.1 | 216 | BGC0000780\|c1\|4692-5402\|+\|no_locus_tag\|3-deoxy-D-manno-octulosonic-acid_kinase\|BAG50455.1 Length=236 | O&K-antigen biosynthetic gene cluster | Vibrio parahaemolyticus  Bacteria; Proteobacteria; Gammaproteobacteria; Vibrionales;  Vibrionaceae; Vibrio. | 28.5 | 0.3 | Identities = 21/57 (37%), Positives = 26/57 (46%), Gaps = 4/57 (7%)  Frame = +3 |
| scaffold_15 | 871 | 2 | 140 | hypothetical protein | Uncharacterized protein PCOAH_00055550 [*Plasmodium coatneyi*] | 99% | 0.001 | 23% | gi\|1139868638\|XP_019917675.1 | 870 | BGC0000093\|c1\|154052-167044\|+\|no_locus_tag\|modular_polyketide_synthase\|ADC45586.1 Length=4330 | Meilingmycin biosynthetic gene cluster | Streptomyces nanchangensis  Bacteria; Actinobacteria; Actinobacteridae; Actinomycetales;  Streptomycineae; Streptomycetaceae; Streptomyces. | 31.2 | 1.1 | Identities = 21/63 (33%), Positives = 29/63 (46%), Gaps = 0/63 (0%)  Frame = +1 |
| scaffold_15 | 1091 | 873 | 141 | hypothetical protein | helicase [*Lachnospiraceae bacterium NLAE-zl-G231*] | 93% | 0.32 | 31% | gi\|1221887044\|WP_089977582.1 | 219 | BGC0001359\|c2\|43973-51199\|-\|no_locus_tag\|Type_I_polyketide_synthase\|CUW01191.1 Length=2408 | PM100117 / PM100118 biosynthetic gene cluster | Streptomyces caniferus  Bacteria; Actinobacteria; Streptomycetales; Streptomycetaceae;  Streptomyces. | 26.6 | 1.7 | Identities = 14/23 (61%), Positives = 15/23 (65%), Gaps = 2/23 (9%)  Frame = -1 |
| scaffold_15 | 1874 | 2101 | 142 | hypothetical protein | GDSL family lipase [*Clostridiales bacterium CHKCI001*] | 84% | 1.2 | 32% | gi\|1221698663\|WP_089826884.1 | 228 | BGC0000661\|c1\|1645-3054\|-\|Npun_R2755\|cyclic_nucleotide-binding_protein\|ACC81292.1 Length=469 | Geosmin biosynthetic gene cluster | Nostoc punctiforme PCC 73102  Bacteria; Cyanobacteria; Nostocales; Nostocaceae; Nostoc. | 26.9 | 1.3 | Identities = 14/41 (34%), Positives = 26/41 (63%), Gaps = 1/41 (2%)  Frame = +1 |
| scaffold_15 | 2101 | 2247 | 143 | hypothetical protein | hypothetical protein [*Vibrio cholerae*] | 93% | 0.87 | 31% | gi\|694132552\|WP_032472795.1 | 147 | BGC0001411\|c1\|28695-29558\|+\|no_locus_tag\|putative_reductase\|AAG26479.1 Length=287 | Polysaccharide B biosynthetic gene cluster | Bacteroides fragilis  Bacteria; Bacteroidetes; Bacteroidia; Bacteroidales; Bacteroidaceae;  Bacteroides. | 25.8 | 1.3 | Identities = 11/31 (35%), Positives = 16/31 (52%), Gaps = 0/31 (0%)  Frame = -1 |
| scaffold_15 | 2409 | 2843 | 144 | hypothetical protein | hypothetical protein AKJ65_02255 [*candidate divison MSBL1 archaeon SCGC-AAA259E19*] | 94% | 4e-08 | 31% | gi\|985663487\|KXA95233.1 | 435 | BGC0001114\|c1\|22470-46580\|+\|no_locus_tag\|TstDEF\|AGN11881.1 Length=8036 | Thailanstatin biosynthetic gene cluster | Burkholderia thailandensis  Bacteria; Proteobacteria; Betaproteobacteria; Burkholderiales;  Burkholderiaceae; Burkholderia; pseudomallei group. | 33.5 | 0.049 | Identities = 16/45 (36%), Positives = 22/45 (49%), Gaps = 1/45 (2%)  Frame = -2 |
| scaffold_15 | 3059 | 3436 | 145 | hypothetical protein | AAA family ATPase [*Coccidioides immitis RS*] | 75% | 0.018 | 30% | gi\|119195753\|XP_001248480.1 | 378 | BGC0000459\|c1\|97608-98756\|-\|no_locus_tag\|sarcosine_oxidase\|AGN74901.1 Length=382 | Griseoviridin / viridogrisein biosynthetic gene cluster | Streptomyces griseoviridis  Bacteria; Actinobacteria; Actinobacteridae; Actinomycetales;  Streptomycineae; Streptomycetaceae; Streptomyces. | 26.2 | 8.4 | Identities = 12/27 (44%), Positives = 17/27 (63%), Gaps = 0/27 (0%)  Frame = +1 |
| scaffold_15 | 3647 | 4105 | 146 | hypothetical protein | PREDICTED: putative sodium-coupled neutral amino acid transporter 10 isoform X4 [*Haplochromis burtoni*] | 76% | 0.40 | 28% | gi\|554845087\|XP_005932893.1 | 459 | BGC0001127\|c1\|41922-43214\|-\|no_locus_tag\|amidase_family_protein\|CCM43858.1 Length=430 | Jagaricin biosynthetic gene cluster | Janthinobacterium agaricidamnosum  Bacteria; Proteobacteria; Betaproteobacteria; Burkholderiales;  Oxalobacteraceae; Janthinobacterium. | 31.6 | 0.24 | Identities = 20/56 (36%), Positives = 27/56 (48%), Gaps = 5/56 (9%)  Frame = +1 |
| scaffold_15 | 4614 | 4850 | 147 | hypothetical protein | MULTISPECIES: HNH endonuclease [*Paenibacillus*] | 89% | 0.002 | 35% | gi\|546575134\|WP_021878590.1 | 237 | BGC0000031\|c1\|501-3107\|+\|no_locus_tag\|hypothetical_protein\|CAE45654.1 Length=868 | Borrelidin biosynthetic gene cluster | Streptomyces parvulus  Bacteria; Actinobacteria; Actinobacteridae; Actinomycetales;  Streptomycineae; Streptomycetaceae; Streptomyces. | 28.5 | 0.54 | Identities = 17/61 (28%), Positives = 26/61 (43%), Gaps = 7/61 (11%)  Frame = +1 |
| scaffold_15 | 4855 | 4932 | **RNA.2** | tRNA-Met-CAT | **tRNA-Met-CAT (annotation by RAST, not by psi-BLAST)** |  |  |  |  |  |  |  |  |  |  |  |
| scaffold_15 | 5700 | 6002 | 148 | hypothetical protein | hypothetical protein [*Borrelia persica*] | 84% | 2.9 | 29% | gi\|1181195104\|WP_084538420.1 | 303 | BGC0001358\|c1\|63670-99575\|+\|VFPBJ_02539\|nonribosomal_peptide_synthase\|OAQ83772.1 Length=11872 | Leucinostatins biosynthetic gene cluster | Purpureocillium lilacinum  Eukaryota; Fungi; Dikarya; Ascomycota; Pezizomycotina;  Sordariomycetes; Hypocreomycetidae; Hypocreales;  Ophiocordycipitaceae; Purpureocillium. | 27.3 | 2.4 | Identities = 16/52 (31%), Positives = 28/52 (54%), Gaps = 17/52 (33%)  Frame = -1 |
| scaffold_15 | 5999 | 6829 | 149 | hypothetical protein | hypothetical protein AKJ58_00980 [*candidate divison MSBL1 archaeon SCGC-AAA385D11*] | 91% | 2e-12 | 28% | gi\|985678035\|KXB08075.1 | 831 | BGC0000862\|c1\|32753-34330\|+\|no_locus_tag\|unknown\|AAB81127.1 Length=525 | Eicosapentaenoic acid biosynthetic gene cluster | Shewanella sp. SCRC-2738  Bacteria; Proteobacteria; Gammaproteobacteria; Alteromonadales;  Shewanellaceae; Shewanella. | 32 | 0.49 | Identities = 25/101 (25%), Positives = 46/101 (46%), Gaps = 6/101 (6%)  Frame = +1 |
| scaffold_15 | 6830 | 7075 | 150 | hypothetical protein | rod shape-determining protein [*Candidatus Harrisonbacteria bacterium CG10_big_fil_rev_8_21_14_0_10_40_38*] | 85% | 0.80 | 33% | gi\|1277192796\|PIR89272.1 | 246 | BGC0000700\|c1\|36122-37168\|-\|no_locus_tag\|putative_metallo_cofactor_biosynthesis_protein\|CAH60160.1 Length=348 | Istamycin biosynthetic gene cluster | Streptomyces tenjimariensis  Bacteria; Actinobacteria; Actinobacteridae; Actinomycetales;  Streptomycineae; Streptomycetaceae; Streptomyces. | 26.9 | 1.6 | Identities = 17/47 (36%), Positives = 26/47 (55%), Gaps = 2/47 (4%)  Frame = -1 |
| scaffold_15 | 7092 | 7301 | 151 | hypothetical protein | rotatin, isoform CRA_a [*Homo sapiens*] | 82% | 3.5 | 32% | gi\|119586920\|EAW66516.1 | 210 | BGC0000380\|c1\|14047-15186\|-\|no_locus_tag\|prolyl-ACP_dehydrogenase\|ADZ24990.1 Length=379 | Leupyrrin biosynthetic gene cluster | Sorangium cellulosum  Bacteria; Proteobacteria; Deltaproteobacteria; Myxococcales;  Sorangiineae; Polyangiaceae; Sorangium. | 26.9 | 1.1 | Identities = 15/36 (42%), Positives = 21/36 (58%), Gaps = 2/36 (6%)  Frame = +1 |
| scaffold_15 | 7875 | 8657 | 152 | hypothetical protein | oxidoreductase [*Mycobacterium sp. Root265*] | 78% | 0.64 | 23% | gi\|950085217\|WP_057170638.1 | 783 | BGC0000054\|c1\|58328-59569\|-\|no_locus_tag\|putative_oxidoreductase\|AAU93812.1 Length=413 | Erythromycin biosynthetic gene cluster | Aeromicrobium erythreum  Bacteria; Actinobacteria; Actinobacteridae; Actinomycetales;  Propionibacterineae; Nocardioidaceae; Aeromicrobium. | 31.2 | 0.79 | Identities = 34/143 (24%), Positives = 56/143 (39%), Gaps = 23/143 (16%)  Frame = +1 |
| scaffold_15 | 8800 | 9084 | 153 | hypothetical protein | class I SAM-dependent methyltransferase [*Lachnoclostridium sp. An138*] | 70% | 4.2 | 30% | gi\|1199570493\|WP_087305847.1 | 285 | BGC0001411\|c1\|5702-6973\|+\|no_locus_tag\|unknown\|AAL61892.1 Length=423 | Polysaccharide B biosynthetic gene cluster | Bacteroides fragilis  Bacteria; Bacteroidetes; Bacteroidia; Bacteroidales; Bacteroidaceae;  Bacteroides. | 28.1 | 0.85 | Identities = 14/43 (33%), Positives = 23/43 (53%), Gaps = 0/43 (0%)  Frame = +1 |
| scaffold_15 | 9102 | 9425 | 154 | hypothetical protein | PadR family transcriptional regulator [*Bacteroidetes bacterium HGW-Bacteroidetes-14*] | 84% | 3e-04 | 36% | gi\|1309047150\|PKP37070.1 | 324 | BGC0000847\|c1\|26980-27393\|-\|no_locus_tag\|putative_cysteine_transferase\|CAD18990.1 Length=137 | Thienamycin biosynthetic gene cluster | Streptomyces cattleya  Bacteria; Actinobacteria; Actinobacteridae; Actinomycetales;  Streptomycineae; Streptomycetaceae; Streptomyces. | 29.6 | 0.32 | Identities = 23/62 (37%), Positives = 35/62 (56%), Gaps = 6/62 (10%)  Frame = +1 |
| scaffold_15 | 9697 | 10143 | 155 | hypothetical protein | hypothetical protein [*Propionibacteriaceae bacterium NML 160184*] | 76% | 1.2 | 28% | gi\|1231902823\|WP_094450608.1 | 447 | BGC0001218\|c1\|13982-26398\|+\|no_locus_tag\|fumosorinone_biosynthesis_polyketide_synthase\|AKC54422.1 Length=4138 | Fumosorinone biosynthetic gene cluster | Isaria fumosorosea  Eukaryota; Fungi; Dikarya; Ascomycota; Pezizomycotina;  Sordariomycetes; Hypocreomycetidae; Hypocreales; Cordycipitaceae;  Isaria. | 29.3 | 1.6 | Identities = 15/42 (36%), Positives = 23/42 (55%), Gaps = 3/42 (7%)  Frame = -1 |
| scaffold_15 | 10149 | 10373 | 156 | hypothetical protein | endonuclease MutS2 [*Thermoanaerobacter kivui*] | 100% | 3.1 | 30% | gi\|902966403\|WP_049685449.1 | 225 | BGC0000304\|c1\|35951-51438\|+\|no_locus_tag\|APS1\|ACZ66258.1 Length=5143 | Apicidin biosynthetic gene cluster | Fusarium incarnatum  Eukaryota; Fungi; Dikarya; Ascomycota; Pezizomycotina;  Sordariomycetes; Hypocreomycetidae; Hypocreales; Nectriaceae;  Fusarium; Fusarium incarnatum-equiseti species complex. | 25 | 6.2 | Identities = 11/38 (29%), Positives = 19/38 (50%), Gaps = 0/38 (0%)  Frame = +1 |
| scaffold_15 | 10406 | 10522 | 157 | hypothetical protein | MULTISPECIES: NUDIX hydrolase [*Paenibacillus*] | 94% | 7.1 | 47% | gi\|506215049\|WP_015734824.1 | 117 | BGC0001043\|c1\|5685-15155\|-\|SGR_814\|putative_NRPS-type-I_PKS_fusion_protein\|BAG17643.1 Length=3156 | SGR PTMs biosynthetic gene cluster | Streptomyces griseus subsp. griseus NBRC 13350  Bacteria; Actinobacteria; Actinobacteridae; Actinomycetales;  Streptomycineae; Streptomycetaceae; Streptomyces. | 24.3 | 3.4 | Identities = 14/35 (40%), Positives = 17/35 (49%), Gaps = 0/35 (0%)  Frame = +1 |
| scaffold_15 | 10547 | 10669 | 158 | hypothetical protein | hypothetical protein [*Terasakiella sp. PR1*] | 97% | 2.6 | 46% | gi\|1061382496\|WP_069185518.1 | 123 | No hits found |  |  |  |  |  |
| scaffold_15 | 10706 | 11893 | 159 | DNA replication helicase protein MCM | ATPase involved in replication control Cdc46/Mcm family [*Candidatus Methanohalarchaeum thermophilum*] | 75% | 4e-97 | 50% | gi\|1124902176\|OKY78794.1 | 1188 | BGC0001281\|c1\|131440-133980\|+\|UMAG_06402\|putative_mini-chromosome_maintenance_complex_protein_7\|XP_011392688.1 Length=846 | Ustilagic acid biosynthetic gene cluster | Ustilago maydis 521  Eukaryota; Fungi; Dikarya; Basidiomycota; Ustilaginomycotina;  Ustilaginomycetes; Ustilaginales; Ustilaginaceae; Ustilago. | 79.3 | 1.00E-15 | Identities = 63/203 (31%), Positives = 95/203 (47%), Gaps = 27/203 (13%)  Frame = +1 |
| scaffold_15 | 12437 | 12066 | 160 | hypothetical protein | LuxR family transcriptional regulator [*Gammaproteobacteria bacterium*] | 73% | 5.8 | 30% | gi\|1273754530\|PIE46336.1 | 372 | BGC0000126\|c1\|70017-72725\|-\|no_locus_tag\|transcriptional_regulator\|BAH02275.1 Length=902 | Pladienolide biosynthetic gene cluster | Streptomyces platensis  Bacteria; Actinobacteria; Actinobacteridae; Actinomycetales;  Streptomycineae; Streptomycetaceae; Streptomyces. | 26.6 | 6.4 | Identities = 28/70 (40%), Positives = 31/70 (44%), Gaps = 20/70 (29%)  Frame = +3 |
| scaffold_15 | 12555 | 12442 | 161 | hypothetical protein | translation initiation factor 2 [*Thermococcus pacificus*] | 81% | 0.005 | 53% | gi\|1214743057\|WP_088854659.1 | 114 | BGC0000928\|c1\|2955-3314\|-\|no_locus_tag\|InsA-like_protein\|ABI75091.1 Length=119 | T3 toxin biosynthetic gene cluster | Cylindrospermopsis raciborskii T3  Bacteria; Cyanobacteria; Nostocales; Nostocaceae;  Cylindrospermopsis. | 30 | 0.021 | Identities = 12/32 (38%), Positives = 20/32 (63%), Gaps = 0/32 (0%)  Frame = +1 |
| scaffold_15 | 12676 | 12557 | 162 | hypothetical protein | hypothetical protein [*Haloterrigena mahii*] | 100% | 0.71 | 41% | gi\|1054523924\|WP_066301516.1 | 120 | BGC0000394\|c1\|30195-39491\|+\|no_locus_tag\|non-ribosomal_peptide_synthetase\|AGD80623.1 Length=3098 | Naphthyridinomycin biosynthetic gene cluster | Streptomyces lusitanus  Bacteria; Actinobacteria; Actinobacteridae; Actinomycetales;  Streptomycineae; Streptomycetaceae; Streptomyces. | 28.1 | 0.11 | Identities = 14/26 (54%), Positives = 19/26 (73%), Gaps = 1/26 (4%)  Frame = -1 |
| scaffold_15 | 13204 | 12677 | 163 | hypothetical protein | hypothetical protein AMJ94_10750 [*Deltaproteobacteria bacterium SM23_61*] | 96% | 3e-30 | 38% | gi\|931511814\|KPK89927.1 | 528 | BGC0001068\|c1\|26861-31855\|+\|ctg1_orf5\|\|ctg1_orf5 Length=1346 | Pyripyropene A biosynthetic gene cluster | Penicillium coprobium PF1169  Eukaryota; Fungi; Dikarya; Ascomycota; Pezizomycotina;  Eurotiomycetes; Eurotiomycetidae; Eurotiales; Aspergillaceae;  Penicillium. | 32 | 0.26 | Identities = 10/29 (34%), Positives = 20/29 (69%), Gaps = 0/29 (0%)  Frame = +1 |
| scaffold_15 | 13486 | 13214 | 164 | hypothetical protein | hypothetical protein [*Thermoflexus hugenholtzii*] | 63% | 0.046 | 37% | gi\|1212021376\|WP_088571670.1 | 273 | BGC0001396\|c1\|67508-68719\|+\|no_locus_tag\|AlmBII\|ANC94983.1 Length=403 | Aldgamycin biosynthetic gene cluster | Streptomyces sp. A1(2016)  Bacteria; Actinobacteria; Streptomycetales; Streptomycetaceae;  Streptomyces. | 27.7 | 1.1 | Identities = 14/26 (54%), Positives = 16/26 (62%), Gaps = 1/26 (4%)  Frame = +3 |
| scaffold_15 | 13631 | 13488 | 165 | hypothetical protein | DUF418 domain-containing protein [*Corynebacterium callunae*] | 63% | 8.5 | 47% | gi\|1174929237\|WP_081602457.1 | 144 | BGC0001318\|c1\|278-1846\|-\|no_locus_tag\|\|Manes.12G132300 Length=457 | Linamarin / Lotaustralin biosynthetic gene cluster. | Manihot esculenta  Eukaryota; Viridiplantae; Streptophyta; Embryophyta; Tracheophyta;  Spermatophyta; Magnoliophyta; eudicotyledons; Gunneridae;  Pentapetalae; rosids; fabids; Malpighiales; Euphorbiaceae;  Crotonoideae; Manihoteae; Manihot. | 26.6 | 0.71 | Identities = 16/41 (39%), Positives = 24/41 (59%), Gaps = 4/41 (10%)  Frame = -2 |
| scaffold_15 | 15131 | 13896 | 166 | hypothetical protein | serine protease [*Shewanella marina*] | 72% | 3e-43 | 40% | gi\|739612508\|WP_037469491.1 | 1236 | BGC0001053\|c1\|26597-27517\|+\|no_locus_tag\|hypothetical_protein\|CAF05641.1 Length=306 | Tubulysin biosynthetic gene cluster | Angiococcus disciformis  Bacteria; Proteobacteria; Deltaproteobacteria; Myxococcales;  Cystobacterineae; Cystobacteraceae; Cystobacter. | 28.9 | 7.6 | Identities = 17/64 (27%), Positives = 32/64 (50%), Gaps = 0/64 (0%)  Frame = +1 |
| scaffold_15 | 15372 | 15169 | 167 | hypothetical protein | ABC transporter permease [*Serratia marcescens*] | 59% | 4.2 | 40% | gi\|505486861\|WP_015671507.1 | 204 | BGC0000440\|c1\|10677-12020\|+\|no_locus_tag\|murF-like_protein\|CAE53342.1 Length=447 | Teicoplanin biosynthetic gene cluster | Actinoplanes teichomyceticus  Bacteria; Actinobacteria; Actinobacteridae; Actinomycetales;  Micromonosporineae; Micromonosporaceae; Actinoplanes. | 27.3 | 0.71 | Identities = 21/56 (38%), Positives = 30/56 (54%), Gaps = 2/56 (4%)  Frame = +1 |
| scaffold_15 | 15724 | 15410 | 168 | hypothetical protein | hypothetical protein PTTG_00890 [*Puccinia triticina 1-1 BBBD Race 1*] | 83% | 0.28 | 32% | gi\|1034739244\|OAV93278.1 | 315 | BGC0000062\|c1\|66684-68006\|+\|no_locus_tag\|ORF20_protein\|AAN74823.1 Length=440 | Fumonisin biosynthetic gene cluster | Fusarium verticillioides  Eukaryota; Fungi; Dikarya; Ascomycota; Pezizomycotina;  Sordariomycetes; Hypocreomycetidae; Hypocreales; Nectriaceae;  Fusarium; Fusarium fujikuroi species complex. | 27.7 | 1.6 | Identities = 15/46 (33%), Positives = 27/46 (59%), Gaps = 4/46 (9%)  Frame = +1 |
| scaffold_15 | 16128 | 16003 | 169 | hypothetical protein | hypothetical protein AGDE_14793 [*Angomonas deanei*] | 92% | 2.7 | 45% | gi\|528216968\|EPY20217.1 | 126 | BGC0001348\|c1\|53713-60162\|+\|IF55_RS32385\|polyketide_synthase\|WP_063764078.1 Length=2149 | JBIR-100 biosynthetic gene cluster | Streptomyces varsoviensis  Bacteria; Actinobacteria; Streptomycetales; Streptomycetaceae;  Streptomyces. | 23.9 | 4 | Identities = 11/19 (58%), Positives = 14/19 (74%), Gaps = 0/19 (0%)  Frame = +2 |
| scaffold_15 | 16258 | 16100 | 170 | hypothetical protein | toxin-antitoxin system protein [*Desulfotomaculum arcticum*] | 59% | 1.2 | 39% | gi\|1224444779\|WP_092467533.1 | 159 | BGC0000951\|c1\|21954-23450\|+\|no_locus_tag\|argininosuccinate_lyase\|ADN26253.1 Length=498 | Pacidamycin biosynthetic gene cluster | Streptomyces coeruleorubidus  Bacteria; Actinobacteria; Actinobacteridae; Actinomycetales;  Streptomycineae; Streptomycetaceae; Streptomyces. | 24.6 | 3.8 | Identities = 9/25 (36%), Positives = 16/25 (64%), Gaps = 0/25 (0%)  Frame = -1 |
| scaffold_15 | 17052 | 16417 | 171 | hypothetical protein | hypothetical protein [*Phaeobacter inhibens*] | 18% | 0.008 | 67% | gi\|992326757\|WP_061048219.1 | 636 | BGC0000591\|c1\|4350-5093\|-\|no_locus_tag\|\|BAA81786.1 Length=247 | Marinostatin biosynthetic gene cluster | Alteromonas sp. B-10-31  Bacteria; Proteobacteria; Gammaproteobacteria; Alteromonadales;  Alteromonadaceae; Alteromonas. | 30.8 | 0.59 | Identities = 14/41 (34%), Positives = 25/41 (61%), Gaps = 1/41 (2%)  Frame = +1 |
| scaffold_15 | 17817 | 17095 | 172 | hypothetical protein | hypothetical protein CSIM01_00387 [*Colletotrichum simmondsii*] | 52% | 0.93 | 28% | gi\|996591206\|KXH34476.1 | 723 | BGC0001186\|c1\|60103-73629\|+\|no_locus_tag\|malonyl_CoA-acyl_carrier_protein_transacylase\|AKQ22697.1 Length=4508 | Misakinolide biosynthetic gene cluster | Candidatus Entotheonella sp. (ex. Theonella swinhoei)  Bacteria; Proteobacteria; Deltaproteobacteria; Candidatus  Entotheonella. | 32.7 | 0.33 | Identities = 14/36 (39%), Positives = 21/36 (58%), Gaps = 0/36 (0%)  Frame = -1 |
| scaffold_15 | 18056 | 17886 | 173 | hypothetical protein | hypothetical protein [*Halorientalis persicus*] | 82% | 2e-09 | 59% | gi\|1224642838\|WP_092662089.1 | 171 | BGC0001093\|c1\|76888-80100\|+\|no_locus_tag\|glycosyl_hydrolase\|AAY32974.1 Length=1070 | Disorazole biosynthetic gene cluster | Sorangium cellulosum  Bacteria; Proteobacteria; Deltaproteobacteria; Myxococcales;  Sorangiineae; Polyangiaceae; Sorangium. | 27.3 | 0.5 | Identities = 13/39 (33%), Positives = 19/39 (49%), Gaps = 0/39 (0%)  Frame = +1 |
| scaffold_15 | 19646 | 18336 | 174 | hypothetical protein-transmembrane prediction | hypothetical protein STSP2_02216 [*Phycisphaerae bacterium ST-NAGAB-D1*] | 81% | 2e-45 | 32% | gi\|1150420369\|AQT69035.1 | 1311 | BGC0000941\|c1\|2342-3619\|-\|SGR_4749\|putative_monooxygenase\|BAG21578.1 Length=425 | Desferrioxamine B biosynthetic gene cluster | Streptomyces griseus subsp. griseus NBRC 13350  Bacteria; Actinobacteria; Actinobacteridae; Actinomycetales;  Streptomycineae; Streptomycetaceae; Streptomyces. | 29.3 | 7.4 | Identities = 20/66 (30%), Positives = 25/66 (38%), Gaps = 12/66 (18%)  Frame = -2 |
| scaffold_15 | 20286 | 19795 | 175 | hypothetical protein | PREDICTED: uncharacterized protein LOC101864606 [*Aplysia californica*] | 39% | 2.9 | 31% | gi\|871223607\|XP_005095448.2 | 492 | BGC0000040\|c1\|5746-14271\|+\|no_locus_tag\|modular_polyketide_synthase\|AAZ94386.1 Length=2841 | Concanamycin A biosynthetic gene cluster | Streptomyces neyagawaensis  Bacteria; Actinobacteria; Actinobacteridae; Actinomycetales;  Streptomycineae; Streptomycetaceae; Streptomyces. | 32 | 0.22 | Identities = 13/38 (34%), Positives = 23/38 (61%), Gaps = 0/38 (0%)  Frame = +1 |
| scaffold_15 | 20450 | 20322 | 176 | hypothetical protein | No significant similarity found. |  |  |  |  | 129 | BGC0000004\|c1\|15203-17096\|+\|no_locus_tag\|putative_ABC_transporter\|BAE71313.1 Length=513 | Aflatoxin biosynthetic gene cluster | Aspergillus oryzae  Eukaryota; Fungi; Dikarya; Ascomycota; Pezizomycotina;  Eurotiomycetes; Eurotiomycetidae; Eurotiales; Aspergillaceae;  Aspergillus. | 25 | 2 | Identities = 10/22 (45%), Positives = 12/22 (55%), Gaps = 0/22 (0%)  Frame = -3 |
| scaffold_15 | 21728 | 20502 | 177 | hypothetical protein | hypothetical protein [*Halovivax asiaticus*] | 57% | 6e-30 | 34% | gi\|494971854\|WP_007697880.1 | 1227 | BGC0000809\|c1\|25737-26543\|-\|no_locus_tag\|D-glucose_O-methyltransferase\|ABC02795.1 Length=268 | AT2433 biosynthetic gene cluster | Actinomadura melliaura  Bacteria; Actinobacteria; Actinobacteridae; Actinomycetales;  Streptosporangineae; Thermomonosporaceae; Actinomadura. | 43.1 | 2.00E-04 | Identities = 32/91 (35%), Positives = 40/91 (44%), Gaps = 19/91 (21%)  Frame = +1 |
| scaffold_15 | 22242 | 21733 | 178 | hypothetical protein | MULTISPECIES: metalloenzyme [*Rhodobacteraceae*] | 88% | 1e-12 | 34% | gi\|1126831250\|WP_075222047.1 | 510 | BGC0001387\|c1\|13365-14534\|-\|no_locus_tag\|NucI\|AMP46600.1 Length=389 | Nucleocidin biosynthetic gene cluster | Streptomyces calvus  Bacteria; Actinobacteria; Streptomycetales; Streptomycetaceae;  Streptomyces. | 34.7 | 0.027 | Identities = 43/160 (27%), Positives = 62/160 (39%), Gaps = 37/160 (23%)  Frame = +1 |
| scaffold_16 | 433 | 179 | 179 | hypothetical protein | DNA annealing helicase and endonuclease ZRANB3 isoform X4 [*Manihot esculenta*] | 84% | 6.3 | 28% | gi\|1216263218\|XP_021615688.1 | 255 | BGC0001108\|c1\|12407-38212\|+\|no_locus_tag\|mixed_type_I_polyketide_synthase/nonribosomal_peptide_synthetase\|AAS47564.1 Length=8601 | Pederin biosynthetic gene cluster | symbiont bacterium of Paederus fuscipes  Bacteria; Unknown. | 32 | 0.036 | Identities = 19/73 (26%), Positives = 35/73 (48%), Gaps = 0/73 (0%)  Frame = -1 |
| scaffold_16 | 652 | 446 | 180 | hypothetical protein | hypothetical protein B6D55_02300 [*Candidatus Omnitrophica bacterium 4484_70.2*] | 77% | 5e-07 | 45% | gi\|1176172345\|OQX87759.1 | 207 | BGC0001186\|c1\|60103-73629\|+\|no_locus_tag\|malonyl_CoA-acyl_carrier_protein_transacylase\|AKQ22697.1 Length=4508 | Misakinolide biosynthetic gene cluster | Candidatus Entotheonella sp. (ex. Theonella swinhoei)  Bacteria; Proteobacteria; Deltaproteobacteria; Candidatus  Entotheonella. | 24.3 | 9.9 | Identities = 11/48 (23%), Positives = 21/48 (44%), Gaps = 0/48 (0%)  Frame = +1 |
| scaffold_16 | 778 | 1008 | 181 | hypothetical protein | CopG family transcriptional regulator [*Microbacterium hydrocarbonoxydans*] | 52% | 0.78 | 45% | gi\|987859638\|WP_060928529.1 | 231 | BGC0001228\|c1\|7404-8180\|-\|B110_RS27175\|MerR_family_transcriptional_regulator\|WP_051419350.1 Length=258 | Retimycin biosynthetic gene cluster | Salinispora arenicola CNT005  Bacteria; Actinobacteria; Micromonosporales; Micromonosporaceae;  Salinispora. | 25.8 | 4 | Identities = 11/23 (48%), Positives = 15/23 (65%), Gaps = 0/23 (0%)  Frame = +3 |
| scaffold_16 | 1308 | 1009 | 182 | hypothetical protein | hypothetical protein AKJ56_01770 [*candidate divison MSBL1 archaeon SCGC-AAA382N08*] | 100% | 9e-16 | 36% | gi\|985678128\|KXB08157.1 | 300 | BGC0001357\|c1\|22374-24119\|+\|no_locus_tag\|carbamoyltransferase\|AMB48445.1 Length=581 | Carbamidocyclophane biosynthetic gene cluster | Nostoc sp. CAVN2  Bacteria; Cyanobacteria; Nostocales; Nostocaceae; Nostoc. | 27.7 | 1.5 | Identities = 18/59 (31%), Positives = 29/59 (49%), Gaps = 4/59 (7%)  Frame = +1 |
| scaffold_16 | 1413 | 2258 | 183 | hypothetical protein | cell envelope integrity protein TolA [*Halofilum ochraceum*] | 64% | 1e-07 | 25% | gi\|1056035201\|WP_067563276.1 | 846 | BGC0001358\|c1\|20959-29243\|-\|VFPBJ_02527\|polyketide_synthase\|OAQ83760.1 Length=2507 | Leucinostatins biosynthetic gene cluster | Purpureocillium lilacinum  Eukaryota; Fungi; Dikarya; Ascomycota; Pezizomycotina;  Sordariomycetes; Hypocreomycetidae; Hypocreales;  Ophiocordycipitaceae; Purpureocillium. | 30.8 | 1.5 | Identities = 16/42 (38%), Positives = 24/42 (57%), Gaps = 1/42 (2%)  Frame = +3 |
| scaffold_16 | 2258 | 2608 | 184 | hypothetical protein | hypothetical protein AKJ57_03695 [*candidate divison MSBL1 archaeon SCGC-AAA259A05*] | 68% | 6e-04 | 33% | gi\|985658385\|KXA90796.1 | 351 | BGC0000972\|c1\|2567-3289\|-\|no_locus_tag\|putative_thioesterase\|CAJ76283.1 Length=240 | Colibactin biosynthetic gene cluster | Escherichia coli  Bacteria; Proteobacteria; Gammaproteobacteria; Enterobacteriales;  Enterobacteriaceae; Escherichia. | 28.9 | 0.78 | Identities = 20/79 (25%), Positives = 31/79 (39%), Gaps = 1/79 (1%)  Frame = -1 |
| scaffold_16 | 2616 | 3044 | 185 | hypothetical protein | DNA-directed DNA polymerase [*Handroanthus impetiginosus*] | 83% | 0.82 | 27% | gi\|1276237728\|PIN00194.1 | 429 | BGC0000816\|c1\|13142-20179\|+\|no_locus_tag\|NRPS\|AGA37267.1 Length=2345 | Malbrancheamide biosynthetic gene cluster | Malbranchea aurantiaca  Eukaryota; Fungi; Dikarya; Ascomycota; Pezizomycotina;  Eurotiomycetes; Eurotiomycetidae; Onygenales; mitosporic Onygenales;  Malbranchea. | 34.7 | 0.021 | Identities = 19/66 (29%), Positives = 31/66 (47%), Gaps = 9/66 (14%)  Frame = +1 |
| scaffold_16 | 3119 | 3394 | 186 | hypothetical protein | hypothetical protein [*Erwinia amylovora*] | 67% | 2.8 | 32% | gi\|1271573075\|WP_099350882.1 | 276 | BGC0001358\|c1\|31871-35196\|+\|VFPBJ_02529\|abc1_domain-containingprotein\|OAQ83762.1 Length=593 | Leucinostatins biosynthetic gene cluster | Purpureocillium lilacinum  Eukaryota; Fungi; Dikarya; Ascomycota; Pezizomycotina;  Sordariomycetes; Hypocreomycetidae; Hypocreales;  Ophiocordycipitaceae; Purpureocillium. | 27.7 | 1.2 | Identities = 14/47 (30%), Positives = 23/47 (49%), Gaps = 0/47 (0%)  Frame = -1 |
| scaffold_16 | 3407 | 3658 | 187 | hypothetical protein | hypothetical protein [*Mycobacterium malmesburyense*] | 93% | 1.7 | 34% | gi\|1222287273\|WP_090340902.1 | 252 | BGC0001034\|c1\|33926-45976\|+\|no_locus_tag\|polyketide_synthase\|CCE88376.1 Length=4016 | Pellasoren biosynthetic gene cluster | Sorangium cellulosum  Bacteria; Proteobacteria; Deltaproteobacteria; Myxococcales;  Sorangiineae; Polyangiaceae; Sorangium. | 30.4 | 0.14 | Identities = 14/34 (41%), Positives = 19/34 (56%), Gaps = 0/34 (0%)  Frame = +1 |
| scaffold_16 | 3766 | 4413 | 188 | hypothetical protein | hypothetical protein AKJ38_03870 [*candidate divison MSBL1 archaeon SCGC-AAA259I14*] | 98% | 3e-17 | 34% | gi\|985664509\|KXA96127.1 | 648 | BGC0000236\|c1\|8908-11067\|+\|no_locus_tag\|putative_ATP-dependent_DNA_helicase\|AAO65333.1 Length=719 | Kinamycin biosynthetic gene cluster | Streptomyces murayamaensis  Bacteria; Actinobacteria; Actinobacteridae; Actinomycetales;  Streptomycineae; Streptomycetaceae; Streptomyces. | 29.6 | 2 | Identities = 18/45 (40%), Positives = 25/45 (56%), Gaps = 2/45 (4%)  Frame = +1 |
| scaffold_16 | 4820 | 4464 | 189 | hypothetical protein | PREDICTED: acetyl-coenzyme A carboxylase carboxyl transferase subunit alpha, chloroplastic-like isoform X2 [*Elaeis guineensis*] | 65% | 0.12 | 40% | gi\|1130648101\|XP_019711051.1 | 357 | BGC0000021\|c1\|30165-32288\|-\|no_locus_tag\|SARP_transcriptional_regulator\|AEP40927.1 Length=707 | Apoptolidin biosynthetic gene cluster | Nocardiopsis sp. FU40  Bacteria; Actinobacteria; Actinobacteridae; Actinomycetales;  Streptosporangineae; Nocardiopsaceae; Nocardiopsis. | 28.1 | 2 | Identities = 12/35 (34%), Positives = 20/35 (57%), Gaps = 0/35 (0%)  Frame = +1 |
| scaffold_16 | 5772 | 4825 | 190 | hypothetical protein | putative transcriptional activator protein [*Eutypa lata UCREL1*] | 33% | 0.13 | 29% | gi\|629669875\|XP_007796889.1 | 948 | BGC0000097\|c1\|52371-62468\|-\|no_locus_tag\|polyketide_synthase_type_I\|ACO94496.1 Length=3365 | ML-449 biosynthetic gene cluster | Streptomyces sp. MP39-85  Bacteria; Actinobacteria; Actinobacteridae; Actinomycetales;  Streptomycineae; Streptomycetaceae; Streptomyces. | 36.2 | 0.035 | Identities = 38/106 (36%), Positives = 46/106 (43%), Gaps = 13/106 (12%)  Frame = -1 |
| scaffold_16 | 6124 | 5855 | 191 | hypothetical protein | hypothetical protein [*Afipia clevelandensis*] | 89% | 2.6 | 33% | gi\|488799166\|WP_002711572.1 | 270 | BGC0000598\|c1\|5349-7361\|+\|no_locus_tag\|C-methyltransferase\|AFS60638.1 Length=670 | Polytheonamides biosynthetic gene cluster | Candidatus Entotheonella sp. TSY1  Bacteria; Proteobacteria; Deltaproteobacteria; Candidatus  Entotheonella. | 25.4 | 7.9 | Identities = 10/29 (34%), Positives = 19/29 (66%), Gaps = 0/29 (0%)  Frame = +3 |
| scaffold_16 | 6123 | 6311 | 192 | hypothetical protein | ammonium transporter [*Prevotellaceae bacterium MN60*] | 62% | 0.40 | 48% | gi\|1228057626\|WP_094150968.1 | 189 | BGC0000304\|c1\|29500-31442\|+\|no_locus_tag\|APS9\|ACZ66255.1 Length=574 | Apicidin biosynthetic gene cluster | Fusarium incarnatum  Eukaryota; Fungi; Dikarya; Ascomycota; Pezizomycotina;  Sordariomycetes; Hypocreomycetidae; Hypocreales; Nectriaceae;  Fusarium; Fusarium incarnatum-equiseti species complex. | 26.6 | 1.2 | Identities = 16/51 (31%), Positives = 24/51 (47%), Gaps = 3/51 (6%)  Frame = -1 |
| scaffold_16 | 6482 | 6724 | 193 | hypothetical protein | H(+)-transporting ATPase [*Anaerococcus sp. HMSC068A02*] | 78% | 0.034 | 37% | gi\|1092710249\|WP_070640579.1 | 243 | BGC0001194\|c1\|64495-65400\|-\|no_locus_tag\|putative_sugar_phosphate_isomerase/epimerase\|BAR73022.1 Length=301 | Nocardiopsin biosynthetic gene cluster | Nocardiopsis sp. CMB-M0232  Bacteria; Actinobacteria; Streptosporangiales; Nocardiopsaceae;  Nocardiopsis. | 25.4 | 6.3 | Identities = 12/36 (33%), Positives = 20/36 (56%), Gaps = 0/36 (0%)  Frame = +3 |
| scaffold_16 | 6804 | 7931 | 194 | hypothetical protein | hypothetical protein [*Burkholderia sp. MSMB1459WGS*] | 89% | 0.036 | 23% | gi\|1177661835\|WP_082751494.1 | 1128 | BGC0000166\|c1\|20010-31199\|+\|no_locus_tag\|tylactone_synthase_modules_4_&_5\|AAB66506.1 Length=3729 | Tylactone biosynthetic gene cluster | Streptomyces fradiae  Bacteria; Actinobacteria; Actinobacteridae; Actinomycetales;  Streptomycineae; Streptomycetaceae; Streptomyces. | 30.4 | 3.4 | Identities = 22/77 (29%), Positives = 32/77 (42%), Gaps = 16/77 (21%)  Frame = -2 |
| scaffold_16 | 7946 | 8143 | 195 | hypothetical protein | ADP-ribosylation factor-like protein 6-interacting protein 4 [*Boleophthalmus pectinirostris*] | 90% | 2.5 | 34% | gi\|1188097993\|XP_020795622.1 | 198 | BGC0001119\|c1\|39214-45816\|+\|no_locus_tag\|divL3_protein\|CCP20050.1 Length=2200 | Divergolide biosynthetic gene cluster | Streptomyces sp. HKI0576  Bacteria; Actinobacteria; Actinobacteridae; Actinomycetales;  Streptomycineae; Streptomycetaceae; Streptomyces. | 26.9 | 0.87 | Identities = 19/60 (32%), Positives = 27/60 (45%), Gaps = 2/60 (3%)  Frame = -3 |
| scaffold_16 | 8137 | 8805 | 196 | hypothetical protein | hypothetical protein [*Nocardia altamirensis*] | 89% | 2e-06 | 28% | gi\|1061069774\|WP_069159667.1 | 669 | BGC0000365\|c1\|41881-43056\|+\|no_locus_tag\|extra_cytoplasmic_sigma_factor\|AAT01810.1 Length=391 | Glycopeptidolipid biosynthetic gene cluster | Mycobacterium smegmatis str. MC2 155  Bacteria; Actinobacteria; Actinobacteridae; Actinomycetales;  Corynebacterineae; Mycobacteriaceae; Mycobacterium. | 27.7 | 7.6 | Identities = 23/67 (34%), Positives = 33/67 (49%), Gaps = 3/67 (4%)  Frame = +1 |
| scaffold_16 | 8805 | 10631 | 197 | hypothetical protein | hypothetical protein A2Y09_01925 [*Planctomycetes bacterium GWA2_39_15*] | 40% | 4e-11 | 32% | gi\|1088281584\|OHB36350.1 | 1827 | BGC0000061\|c1\|28983-60860\|-\|no_locus_tag\|FscC\|AAQ82564.1 Length=10625 | FR-008 biosynthetic gene cluster | Streptomyces sp. FR-008  Bacteria; Actinobacteria; Actinobacteridae; Actinomycetales;  Streptomycineae; Streptomycetaceae; Streptomyces. | 33.5 | 0.63 | Identities = 20/63 (32%), Positives = 28/63 (44%), Gaps = 9/63 (14%)  Frame = +1 |
| scaffold_16 | 10665 | 11534 | 198 | hypothetical protein | fibronectin [*Geobacter soli*] | 77% | 1e-04 | 27% | gi\|745695590\|KIE42013.1 | 870 | No hits found |  |  |  |  |  |
| scaffold_16 | 11551 | 11832 | 199 | hypothetical protein | sulfite exporter TauE/SafE family protein [*Methanosarcina spelaei*] | 93% | 2.8 | 26% | gi\|1240481481\|WP_095645183.1 | 282 | BGC0000441\|c1\|84452-85345\|-\|no_locus_tag\|transposase\|CAG15044.1 Length=297 | Teicoplanin biosynthetic gene cluster | Actinoplanes teichomyceticus  Bacteria; Actinobacteria; Actinobacteridae; Actinomycetales;  Micromonosporineae; Micromonosporaceae; Actinoplanes. | 27.7 | 1.2 | Identities = 17/58 (29%), Positives = 29/58 (50%), Gaps = 1/58 (2%)  Frame = -1 |
| scaffold_16 | 11832 | 13133 | 200 | peptidase S8 and S53, subtilisin, kexin, sedolisin | Subtilase family protein [*Micromonospora cremea*] | 37% | 1e-11 | 33% | gi\|1118770207\|SIM81338.1 | 1302 | BGC0001101\|c1\|7431-11144\|+\|no_locus_tag\|subtilisin-like_secreted_protease\|AAN85481.1 Length=1237 | Leinamycin biosynthetic gene cluster | Streptomyces atroolivaceus  Bacteria; Actinobacteria; Actinobacteridae; Actinomycetales;  Streptomycineae; Streptomycetaceae; Streptomyces. | 62.4 | 4.00E-10 | Identities = 58/186 (31%), Positives = 89/186 (48%), Gaps = 25/186 (13%)  Frame = +1 |
| scaffold_16 | 13335 | 17771 | 201 | predicted protein | hypothetical protein [*Halorubrum sp. SD683*] | 16% | 8e-20 | 32% | gi\|1196569719\|WP_086216819.1 | 4437 | BGC0000171\|c1\|6799-8133\|-\|no_locus_tag\|signal_transduction_histidine_kinase\|CCC21117.1 Length=444 | 9-methylstreptimidone biosynthetic gene cluster | Streptomyces himastatinicus ATCC 53653  Bacteria; Actinobacteria; Actinobacteridae; Actinomycetales;  Streptomycineae; Streptomycetaceae; Streptomyces. | 34.7 | 0.65 | Identities = 22/77 (29%), Positives = 33/77 (43%), Gaps = 6/77 (8%)  Frame = +1 |
| scaffold_16 | 18334 | 17825 | 202 | Geranylgeranyl diphosphate synthase (EC 2.5.1.29) | hypothetical protein AKJ41_03425 [*candidate divison MSBL1 archaeon SCGC-AAA259O05*] | 100% | 8e-80 | 75% | gi\|985669946\|KXB00917.1 | 510 | BGC0000648\|c1\|1058-2143\|+\|no_locus_tag\|geranylgeranyl_pyrophosphate_synthetase\|CAA79955.1 Length=361 | Carotenoid biosynthetic gene cluster | Myxococcus xanthus  Bacteria; Proteobacteria; Deltaproteobacteria; Myxococcales;  Cystobacterineae; Myxococcaceae; Myxococcus. | 76.3 | 9.00E-17 | Identities = 58/172 (34%), Positives = 92/172 (53%), Gaps = 9/172 (5%)  Frame = +1 |
| scaffold_2 | 51 | 224 | 203 | hypothetical protein | putative uncharacterized protein [*Ruminococcus sp. CAG:488*] | 63% | 0.60 | 39% | gi\|524256245\|CDA19440.1 | 174 | BGC0001069\|c1\|47872-61797\|+\|no_locus_tag\|hybrid_nonribosomal_peptide_synthetase/polyketide_synthase\|AAY89051.1 Length=4641 | Chivosazole biosynthetic gene cluster | Sorangium cellulosum  Bacteria; Proteobacteria; Deltaproteobacteria; Myxococcales;  Sorangiineae; Polyangiaceae; Sorangium. | 25.4 | 2.4 | Identities = 22/46 (48%), Positives = 23/46 (50%), Gaps = 4/46 (9%)  Frame = -1 |
| scaffold_2 | 258 | 458 | 204 | hypothetical protein | enoyl-[acyl-carrier-protein] reductase FabI [*Vibrio panuliri*] | 100% | 0.87 | 30% | gi\|1130951397\|WP_075716190.1 | 201 | BGC0001015\|c1\|13178-23644\|+\|no_locus_tag\|peptide_synthetase\|CAD29794.1 Length=3488 | Microcystin biosynthetic gene cluster | Planktothrix agardhii NIVA-CYA 126/8  Bacteria; Cyanobacteria; Oscillatoriophycideae; Oscillatoriales;  Planktothrix. | 28.9 | 0.22 | Identities = 20/62 (32%), Positives = 32/62 (52%), Gaps = 1/62 (2%)  Frame = -1 |
| scaffold_2 | 448 | 798 | 205 | hypothetical protein | hypothetical protein [*Candidatus Paracaedibacter acanthamoebae*] | 74% | 1.6 | 31% | gi\|740677486\|WP_038462775.1 | 351 | BGC0001415\|c1\|885-16928\|-\|SMDB11_2289\|non-ribosomal_peptide_synthetase\|CDG12864.1 Length=5347 | Althiomycin biosynthetic gene cluster | Serratia marcescens subsp. marcescens Db11  Bacteria; Proteobacteria; Gammaproteobacteria; Enterobacteriales;  Enterobacteriaceae; Serratia. | 28.9 | 1.2 | Identities = 16/34 (47%), Positives = 22/34 (65%), Gaps = 3/34 (9%)  Frame = +1 |
| scaffold_2 | 860 | 1033 | 206 | hypothetical protein | No significant similarity found. |  |  |  |  | 174 | BGC0001288\|c1\|130030-141753\|+\|no_locus_tag\|type_I_polyketide_synthase\|BAQ25513.1 Length=3907 | Maklamicin biosynthetic gene cluster | Micromonospora sp. GMKU326  Bacteria; Actinobacteria; Micromonosporales; Micromonosporaceae;  Micromonospora. | 24.6 | 5.9 | Identities = 8/27 (30%), Positives = 14/27 (52%), Gaps = 0/27 (0%)  Frame = +1 |
| scaffold_2 | 1026 | 2036 | 207 | hypothetical protein | hypothetical protein AKJ43_03215 [*candidate divison MSBL1 archaeon SCGC-AAA261D19*] | 38% | 4e-14 | 39% | gi\|985670760\|KXB01637.1 | 1011 | BGC0001100\|c1\|5129-6838\|+\|no_locus_tag\|putative_ABC_transporter_protein\|BAC76468.1 Length=569 | Lankacidin biosynthetic gene cluster | Streptomyces rochei  Bacteria; Actinobacteria; Actinobacteridae; Actinomycetales;  Streptomycineae; Streptomycetaceae; Streptomyces. | 30.8 | 1.9 | Identities = 15/31 (48%), Positives = 21/31 (68%), Gaps = 1/31 (3%)  Frame = +2 |
| scaffold_2 | 2596 | 2784 | 208 | hypothetical protein | transposase [*Gorillibacterium sp. SN4*] | 74% | 3.6 | 39% | gi\|960408518\|WP_058300473.1 | 189 | BGC0001356\|c1\|3878-5701\|+\|PDENDC454_19528\|ABC_transporter\|EHQ60564.1 Length=607 | Paeninodin biosynthetic gene cluster | Paenibacillus dendritiformis C454  Bacteria; Firmicutes; Bacilli; Bacillales; Paenibacillaceae;  Paenibacillus. | 27.3 | 0.72 | Identities = 19/64 (30%), Positives = 36/64 (56%), Gaps = 11/64 (17%)  Frame = -2 |
| scaffold_2 | 3992 | 4690 | 209 | hypothetical protein | DNA mismatch repair protein MutS [*Nitrospira sp. UW-LDO-02*] | 47% | 0.078 | 31% | gi\|1231960528\|OYT24569.1 | 699 | BGC0001165\|c1\|11137-18072\|+\|no_locus_tag\|CurA\|AAT70096.1 Length=2311 | Curacin biosynthetic gene cluster | Lyngbya majuscula  Bacteria; Cyanobacteria; Oscillatoriophycideae; Oscillatoriales;  Lyngbya. | 32.7 | 0.24 | Identities = 26/98 (27%), Positives = 46/98 (47%), Gaps = 15/98 (15%)  Frame = -1 |
| scaffold_2 | 4684 | 5163 | 210 | hypothetical protein | SAM-dependent methyltransferase [*Aquimarina latercula*] | 53% | 1e-04 | 34% | gi\|653143906\|WP_027393135.1 | 480 | BGC0001071\|c1\|54474-55802\|+\|no_locus_tag\|acyl_transferase\|ADA69247.1 Length=442 | Nosperin biosynthetic gene cluster | Nostoc sp. 'Peltigera membranacea cyanobiont'  Bacteria; Cyanobacteria; Nostocales; Nostocaceae; Nostoc. | 29.6 | 1.1 | Identities = 25/96 (26%), Positives = 41/96 (43%), Gaps = 13/96 (14%)  Frame = -1 |
| scaffold_2 | 5160 | 5477 | 211 | hypothetical protein | TPA: adenylate kinase [*Candidatus Gastranaerophilales bacterium HUM_18*] | 57% | 0.74 | 35% | gi\|1248382624\|DAB12560.1 | 318 | BGC0000471\|c1\|3182-5512\|+\|Osc7112_3651\|bacteriocin_biosynthesis_cyclodehydratase_domain_protein\|AFZ08005.1 Length=776 | Viridisamide A biosynthetic gene cluster | Oscillatoria nigro-viridis PCC 7112  Bacteria; Cyanobacteria; Oscillatoriophycideae; Oscillatoriales;  Oscillatoria. | 29.6 | 0.36 | Identities = 21/76 (28%), Positives = 33/76 (43%), Gaps = 14/76 (18%)  Frame = +1 |
| scaffold_2 | 5834 | 6010 | 212 | hypothetical protein | glycosyltransferase family 1 protein [*Methanothermococcus thermolithotrophicus*] | 77% | 7.9 | 40% | gi\|1180357545\|WP_083876345.1 | 177 | BGC0000952\|c3\|3398-4645\|-\|no_locus_tag\|HMG-CoA_synthase-like_protein\|CBW45695.1 Length=415 | Pristinamycin biosynthetic gene cluster | Streptomyces pristinaespiralis  Bacteria; Actinobacteria; Actinobacteridae; Actinomycetales;  Streptomycineae; Streptomycetaceae; Streptomyces. | 28.5 | 0.21 | Identities = 16/49 (33%), Positives = 25/49 (51%), Gaps = 12/49 (24%)  Frame = -1 |
| scaffold_2 | 6132 | 6584 | 213 | hypothetical protein | MULTISPECIES: M48 family peptidase [*Synechococcus*] | 65% | 1.4 | 30% | gi\|501263164\|WP_012306182.1 | 453 | BGC0000097\|c1\|7108-26259\|+\|no_locus_tag\|polyketide_synthase_type_I\|ACO94483.1 Length=6383 | ML-449 biosynthetic gene cluster | Streptomyces sp. MP39-85  Bacteria; Actinobacteria; Actinobacteridae; Actinomycetales;  Streptomycineae; Streptomycetaceae; Streptomyces. | 29.6 | 1.2 | Identities = 13/39 (33%), Positives = 23/39 (59%), Gaps = 0/39 (0%)  Frame = +1 |
| scaffold_2 | 6581 | 6730 | 214 | hypothetical protein | sensor histidine kinase [*Nocardia elegans*] | 87% | 2.1 | 40% | gi\|1016354082\|WP_063023664.1 | 150 | BGC0000084\|c1\|72767-83737\|-\|no_locus_tag\|laidlomycin_polyketide_synthase_(module_11_and_module_12)\|AFL48532.1 Length=3656 | Laidlomycin biosynthetic gene cluster | Streptomyces sp. CS684  Bacteria; Actinobacteria; Actinobacteridae; Actinomycetales;  Streptomycineae; Streptomycetaceae; Streptomyces. | 26.6 | 0.68 | Identities = 12/37 (32%), Positives = 19/37 (51%), Gaps = 0/37 (0%)  Frame = +3 |
| scaffold_2 | 7259 | 7543 | 215 | hypothetical protein | acetyl-CoA carboxylase carboxyltransferase subunit alpha [*Bacillus pumilus*] | 81% | 0.65 | 32% | gi\|639690407\|WP_024720150.1 | 285 | BGC0000186\|c1\|9683-23632\|-\|BTH_II1665\|polyketide_synthase,_putative\|ABC33986.1 Length=4649 | Thailandamide biosynthetic gene cluster | Burkholderia thailandensis E264  Bacteria; Proteobacteria; Betaproteobacteria; Burkholderiales;  Burkholderiaceae; Burkholderia; pseudomallei group. | 27.7 | 1.6 | Identities = 14/47 (30%), Positives = 20/47 (43%), Gaps = 0/47 (0%)  Frame = -2 |
| scaffold_2 | 7694 | 8554 | 216 | Chlorophyll a synthase ChlG (EC 2.5.1.62) | hypothetical protein AKJ39_02905 [*candidate divison MSBL1 archaeon SCGC-AAA259J03*] | 82% | 2e-69 | 47% | gi\|985666538\|KXA97890.1 | 861 | BGC0000806\|c1\|6015-6896\|+\|no_locus_tag\|4-hydroxybenzoate_polyprenyltransferase\|AHL24453.1 Length=293 | Phosphonoglycans biosynthetic gene cluster | Glycomyces sp. NRRL B-16210  Bacteria; Actinobacteria; Actinobacteridae; Actinomycetales;  Glycomycineae; Glycomycetaceae; Glycomyces. | 44.3 | 5.00E-05 | Identities = 50/199 (25%), Positives = 89/199 (45%), Gaps = 20/199 (10%)  Frame = +1 |
| scaffold_2 | 8915 | 8637 | 217 | hypothetical protein | hypothetical protein AKJ58_00375 [*candidate divison MSBL1 archaeon SCGC-AAA385D11*] | 66% | 8e-19 | 48% | gi\|985678275\|KXB08288.1 | 279 | BGC0000330\|c1\|22369-31653\|-\|H16_B1687\|Non-ribosomal_peptide_synthetase\|CAJ96472.1 Length=3094 | Cupriachelin biosynthetic gene cluster | Ralstonia eutropha H16  Bacteria; Proteobacteria; Betaproteobacteria; Burkholderiales;  Burkholderiaceae; Cupriavidus. | 26.2 | 4.7 | Identities = 13/31 (42%), Positives = 16/31 (52%), Gaps = 3/31 (10%)  Frame = +1 |
| scaffold_2 | 9816 | 8962 | 218 | NAD-dependent glyceraldehyde-3-phosphate dehydrogenase (EC 1.2.1.12) | glyceraldehyde-3-phosphate dehydrogenase [*candidate divison MSBL1 archaeon SCGC-AAA261F17*] | 99% | 4e-166 | 83% | gi\|985671588\|KXB02384.1 | 855 | BGC0000678\|c1\|1-1005\|+\|SAV_2990\|glyceraldehyde-3-phosphate_dehydrogenase\|BAC70701.1 Length=334 | Pentalenolactone biosynthetic gene cluster | Streptomyces avermitilis MA-4680 = NBRC 14893  Bacteria; Actinobacteria; Actinobacteridae; Actinomycetales;  Streptomycineae; Streptomycetaceae; Streptomyces. | 226 | 2.00E-72 | Identities = 137/280 (49%), Positives = 175/280 (63%), Gaps = 3/280 (1%)  Frame = +1 |
| scaffold_2 | 9970 | 9818 | 219 | NAD-dependent glyceraldehyde-3-phosphate dehydrogenase (EC 1.2.1.12) | glyceraldehyde-3-phosphate dehydrogenase [*candidate divison MSBL1 archaeon SCGC-AAA261F17*] | 92% | 2e-18 | 89% | gi\|985671588\|KXB02384.1 | 153 | BGC0000678\|c1\|1-1005\|+\|SAV_2990\|glyceraldehyde-3-phosphate_dehydrogenase\|BAC70701.1 Length=334 | Pentalenolactone biosynthetic gene cluster | Streptomyces avermitilis MA-4680 = NBRC 14893  Bacteria; Actinobacteria; Actinobacteridae; Actinomycetales;  Streptomycineae; Streptomycetaceae; Streptomyces. | 57.4 | 7.00E-12 | Identities = 26/46 (57%), Positives = 32/46 (70%), Gaps = 0/46 (0%)  Frame = +1 |
| scaffold_2 | 10091 | 10246 | 220 | hypothetical protein | hypothetical protein [*Marinimicrobium agarilyticum*] | 82% | 3.2 | 40% | gi\|653078825\|WP_027329414.1 | 156 | BGC0000703\|c1\|77717-78412\|-\|no_locus_tag\|hypothetical_protein\|BAE95479.1 Length=231 | Kanamycin biosynthetic gene cluster | Streptomyces kanamyceticus  Bacteria; Actinobacteria; Actinobacteridae; Actinomycetales;  Streptomycineae; Streptomycetaceae; Streptomyces. | 24.3 | 5.4 | Identities = 14/50 (28%), Positives = 22/50 (44%), Gaps = 1/50 (2%)  Frame = +3 |
| scaffold_2 | 10532 | 10705 | 221 | hypothetical protein | hypothetical protein [*Actinobacillus minor*] | 75% | 4.1 | 41% | gi\|492357103\|WP_005819686.1 | 174 | BGC0001393\|c1\|5584-12708\|+\|SLUG_08100\|non-ribosomal_peptide_synthetase\|CCB53264.1 Length=2374 | Lugdunin biosynthetic gene cluster | Staphylococcus lugdunensis N920143  Bacteria; Firmicutes; Bacilli; Bacillales; Staphylococcaceae;  Staphylococcus. | 25.8 | 1.8 | Identities = 15/53 (28%), Positives = 26/53 (49%), Gaps = 12/53 (23%)  Frame = +1 |
| scaffold_2 | 10768 | 11121 | 222 | hypothetical protein | peptide chain release factor 1 [*Sphaerobacter thermophilus*] | 67% | 0.007 | 34% | gi\|502635853\|WP_012872379.1 | 354 | BGC0000935\|c1\|26523-27314\|+\|no_locus_tag\|EsmB5\|AFB35636.1 Length=263 | Esmeraldin biosynthetic gene cluster | Streptomyces antibioticus  Bacteria; Actinobacteria; Actinobacteridae; Actinomycetales;  Streptomycineae; Streptomycetaceae; Streptomyces. | 27.7 | 2.1 | Identities = 19/52 (37%), Positives = 25/52 (48%), Gaps = 9/52 (17%)  Frame = -1 |
| scaffold_2 | 11271 | 11137 | 223 | hypothetical protein | transcription-repair coupling factor [*Deinococcus sp. Leaf326*] | 88% | 4.3 | 45% | gi\|947639067\|WP_056302012.1 | 135 | BGC0001026\|c1\|4157-16022\|+\|no_locus_tag\|hybrid_PKS-NRPS_protein\|ACS68554.1 Length=3935 | NG-391 biosynthetic gene cluster | Metarhizium anisopliae  Eukaryota; Fungi; Dikarya; Ascomycota; Pezizomycotina;  Sordariomycetes; Hypocreomycetidae; Hypocreales; Clavicipitaceae;  mitosporic Clavicipitaceae; Metarhizium. | 25.8 | 0.98 | Identities = 14/33 (42%), Positives = 19/33 (58%), Gaps = 0/33 (0%)  Frame = +1 |
| scaffold_2 | 11905 | 12297 | 224 | hypothetical protein | translocation/assembly module TamB [*Flavobacterium filum*] | 80% | 1.1 | 26% | gi\|652300232\|WP_026709965.1 | 393 | BGC0000118\|c1\|20808-22544\|+\|no_locus_tag\|methyltransferase\|BAF92597.1 Length=578 | Pactamycin biosynthetic gene cluster | Streptomyces pactum  Bacteria; Actinobacteria; Actinobacteridae; Actinomycetales;  Streptomycineae; Streptomycetaceae; Streptomyces. | 28.1 | 2.4 | Identities = 18/67 (27%), Positives = 32/67 (48%), Gaps = 5/67 (7%)  Frame = +1 |
| scaffold_2 | 13543 | 13803 | 225 | hypothetical protein | aspartate--tRNA ligase [*Geomicrobium sp. JCM 19039*] | 67% | 4.8 | 41% | gi\|755072728\|WP_042424346.1 | 261 | BGC0001042\|c1\|19885-30714\|+\|no_locus_tag\|non-ribosomal_peptide_synthetase\|ACY06285.1 Length=3609 | Sanglifehrin A biosynthetic gene cluster | Streptomyces flaveolus  Bacteria; Actinobacteria; Actinobacteridae; Actinomycetales;  Streptomycineae; Streptomycetaceae; Streptomyces. | 27.7 | 1.3 | Identities = 15/47 (32%), Positives = 20/47 (43%), Gaps = 4/47 (9%)  Frame = +1 |
| scaffold_2 | 14082 | 14201 | 226 | hypothetical protein | No significant similarity found. |  |  |  |  | 120 | BGC0000407\|c1\|1526-2503\|+\|no_locus_tag\|putative_epimerase\|AEW31017.1 Length=325 | Plipastatin biosynthetic gene cluster | Bacillus subtilis subsp. subtilis  Bacteria; Firmicutes; Bacilli; Bacillales; Bacillaceae; Bacillus. | 25 | 1.9 | Identities = 10/21 (48%), Positives = 15/21 (71%), Gaps = 0/21 (0%)  Frame = +1 |
| scaffold_3 | 966 | 820 | 227 | hypothetical protein | No significant similarity found. |  |  |  |  | 138 | BGC0001103\|c1\|3161-15076\|+\|no_locus_tag\|MycA\|AAF08795.1 Length=3971 | Mycosubtilin biosynthetic gene cluster | Bacillus subtilis subsp. spizizenii ATCC 6633  Bacteria; Firmicutes; Bacilli; Bacillales; Bacillaceae; Bacillus. | 24.3 | 3.7 | Identities = 11/44 (25%), Positives = 20/44 (45%), Gaps = 0/44 (0%)  Frame = +1 |
| scaffold_3 | 1225 | 1377 | 228 | hypothetical protein | DUF3581 domain-containing protein [*Methylobacter marinus*] | 92% | 2.9 | 29% | gi\|750309206\|WP_040591177.1 | 285 | GC0000027\|c1\|57499-59399\|+\|2377\|solute_carrier_family_40_member_1\|AGN71619.1 Length=405 | Azaphilone biosynthetic gene cluster | Monascus pilosus  Eukaryota; Fungi; Dikarya; Ascomycota; Pezizomycotina;  Eurotiomycetes; Eurotiomycetidae; Eurotiales; Aspergillaceae;  Monascus. | 27.3 | 1.6 | Identities = 17/53 (32%), Positives = 29/53 (55%), Gaps = 2/53 (4%)  Frame = +1 |
| scaffold_3 | 1784 | 2095 | 229 | hypothetical protein | cobalt-precorrin 5A hydrolase [*Hyphomicrobium facile*] | 93% | 0.56 | 46% | gi\|1097765764\|SFV26573.1 | 144 | BGC0000735\|c1\|6018-6998\|-\|no_locus_tag\|BcbI\|AAF67271.1 Length=326 | Capsular polysaccharide biosynthetic gene cluster | Pasteurella multocida  Bacteria; Proteobacteria; Gammaproteobacteria; Pasteurellales;  Pasteurellaceae; Pasteurella. | 24.6 | 3.7 | Identities = 11/33 (33%), Positives = 17/33 (52%), Gaps = 0/33 (0%)  Frame = +3 |
| scaffold_3 | 2131 | 2526 | 230 | hypothetical protein | MFS transporter [*Micrococcales bacterium 73-15*] | 87% | 4.9 | 33% | gi\|1113534312\|OJX94649.1 | 213 | BGC0001354\|c1\|6806-8557\|+\|no_locus_tag\|3-hydroxybutyryl-CoA_dehydrogenase\|CUI25739.1 Length=583 | U-68204 biosynthetic gene cluster | Streptomyces thiolactonus  Bacteria; Actinobacteria; Streptomycetales; Streptomycetaceae;  Streptomyces. | 25.8 | 3.6 | Identities = 22/68 (32%), Positives = 30/68 (44%), Gaps = 10/68 (15%)  Frame = +1 |
| scaffold_3 | 3169 | 3348 | 231 | hypothetical protein | ABC transporter permease [*Desulfitobacterium sp. PCE1*] | 86% | 0.076 | 32% | gi\|518688679\|WP_019850372.1 | 417 | BGC0001323\|c1\|62414-69860\|+\|no_locus_tag\|terpene_synthase_18\|AGK82823.1 Length=770 | Monoterpenes-diterpenes biosynthetic gene cluster | Solanum pennellii  Eukaryota; Viridiplantae; Streptophyta; Embryophyta; Tracheophyta;  Spermatophyta; Magnoliophyta; eudicotyledons; Gunneridae;  Pentapetalae; asterids; lamiids; Solanales; Solanaceae; Solanoideae;  Solaneae; Solanum; Lycopersicon. | 28.9 | 1.4 | Identities = 13/46 (28%), Positives = 22/46 (48%), Gaps = 1/46 (2%)  Frame = +1 |
| scaffold_3 | 3824 | 3937 | 232 | hypothetical protein | peptidase S8/S53 subtilisin kexin sedolisin [*Halothermothrix orenii*] | 82% | 8e-14 | 35% | gi\|501769773\|WP_012635463.1 | 660 | BGC0000473\|c1\|868-2841\|+\|no_locus_tag\|subtilisin-like_protein\|CAO82081.1 Length=657 | Microcyclamide biosynthetic gene cluster | Microcystis aeruginosa NIES-298  Bacteria; Cyanobacteria; Oscillatoriophycideae; Chroococcales;  Microcystis. | 33.9 | 0.08 | Identities = 27/78 (35%), Positives = 39/78 (50%), Gaps = 16/78 (21%)  Frame = +1 |
| scaffold_3 | 3934 | 4059 | 233 | hypothetical protein | exopolysaccharide biosynthesis protein ExoD [*Rhizobium sp. CF097*] | 79% | 0.044 | 36% | gi\|739259268\|WP_037122249.1 | 282 | BGC0001096\|c1\|62806-70794\|+\|no_locus_tag\|type_I_polyketide_synthase\|ADH01489.1 Length=2662 | FR901464 biosynthetic gene cluster | Pseudomonas sp. 2663  Bacteria; Proteobacteria; Gammaproteobacteria; Pseudomonadales;  Pseudomonadaceae; Pseudomonas. | 26.6 | 3.3 | Identities = 14/55 (25%), Positives = 23/55 (42%), Gaps = 10/55 (18%)  Frame = +3 |
| scaffold_3 | 5837 | 5974 | 234 | hypothetical protein | MFS transporter [*Curtobacterium ammoniigenes*] | 89% | 0.93 | 33% | gi\|1180005354\|WP_083527708.1 | 285 | BGC0001096\|c1\|53637-62690\|+\|no_locus_tag\|polyketide_synthase\|ADH01488.1 Length=3017 | FR901464 biosynthetic gene cluster | Pseudomonas sp. 2663  Bacteria; Proteobacteria; Gammaproteobacteria; Pseudomonadales;  Pseudomonadaceae; Pseudomonas. | 28.9 | 0.53 | Identities = 12/39 (31%), Positives = 20/39 (51%), Gaps = 0/39 (0%)  Frame = +1 |
| scaffold_3 | 6692 | 6976 | 235 | hypothetical protein | hypothetical protein [*Cnuibacter physcomitrellae*] | 67% | 1.4 | 24% | gi\|1183819120\|WP_085017771.1 | 1023 | BGC0000349\|c1\|3828-20117\|+\|SACE_3035\|putative_non-ribosomal_peptide_synthetase\|CAM02313.1 Length=5429 | Erythrochelin biosynthetic gene cluster | Saccharopolyspora erythraea NRRL 2338  Bacteria; Actinobacteria; Actinobacteridae; Actinomycetales;  Pseudonocardineae; Pseudonocardiaceae; Saccharopolyspora. | 29.3 | 5.7 | Identities = 32/81 (40%), Positives = 37/81 (46%), Gaps = 7/81 (9%)  Frame = -1 |
| scaffold_3 | 7264 | 7407 | 236 | hypothetical protein | S9 family peptidase [*Nocardia pseudobrasiliensis*] | 68% | 0.79 | 49% | gi\|1056574138\|WP_068006013.1 | 192 | BGC0000435\|c1\|10568-28357\|+\|PSPTO_2830\|non-ribosomal_peptide_synthetase_SyfB\|AAO56329.1 Length=5929 | Syringafactin biosynthetic gene cluster | Pseudomonas syringae pv. tomato str. DC3000  Bacteria; Proteobacteria; Gammaproteobacteria; Pseudomonadales;  Pseudomonadaceae; Pseudomonas. | 28.9 | 0.21 | Identities = 16/37 (43%), Positives = 25/37 (68%), Gaps = 5/37 (14%)  Frame = -2 |
| scaffold_3 | 7639 | 7427 | 237 | hypothetical protein | hypothetical protein [*Methanosphaera sp. WGK6*] | 82% | 7e-07 | 20% | gi\|1068541903\|WP_069592640.1 | 1929 | BGC0000326\|c1\|47536-48684\|+\|no_locus_tag\|prephenate_dehydrogenase\|AAK81837.1 Length=382 | Complestatin biosynthetic gene cluster | Streptomyces lavendulae  Bacteria; Actinobacteria; Actinobacteridae; Actinomycetales;  Streptomycineae; Streptomycetaceae; Streptomyces. | 30 | 6.8 | Identities = 23/79 (29%), Positives = 37/79 (47%), Gaps = 11/79 (14%)  Frame = +1 |
| scaffold_3 | 8067 | 7651 | 238 | hypothetical protein | Uncharacterized protein LW93_12271 [*Fusarium fujikuroi*] | 55% | 2e-06 | 33% | gi\|829112408\|KLO88856.1 | 702 | BGC0000369\|c1\|27532-38469\|+\|no_locus_tag\|non-ribosomal_peptide_synthase\|AHZ20774.1 Length=3645 | Hassallidins biosynthetic gene cluster | Anabaena sp. Syke748  Bacteria; Cyanobacteria; Nostocales; Nostocaceae; Anabaena. | 33.9 | 0.13 | Identities = 16/46 (35%), Positives = 25/46 (54%), Gaps = 0/46 (0%)  Frame = -1 |
| scaffold_3 | 8950 | 8291 | 239 | serine alkaline protease (subtilisin E)( EC:3.4.21.62 ) | No significant similarity found. |  |  |  |  | 132 | BGC0001056\|c1\|44508-49103\|+\|no_locus_tag\|putative_polyketide_synthase\|CCM44338.1 Length=1531 | Zeamine biosynthetic gene cluster | Serratia plymuthica RVH1  Bacteria; Proteobacteria; Gammaproteobacteria; Enterobacteriales;  Enterobacteriaceae; Serratia. | 25 | 1.8 | dentities = 12/27 (44%), Positives = 17/27 (63%), Gaps = 0/27 (0%)  Frame = +2 |
| scaffold_3 | 9865 | 9584 | 240 | hypothetical protein | hypothetical protein B6U84_05395 [*Candidatus Bathyarchaeota archaeon ex4484_40*] | 64% | 1e-07 | 28% | gi\|1231981811\|OYT43840.1 | 873 | BGC0001413\|c1\|7-3810\|-\|no_locus_tag\|CysT\|AKP45383.1 Length=1267 | Cystobactamide biosynthetic gene cluster | Cystobacter sp. Cbv34  Bacteria; Proteobacteria; Deltaproteobacteria; Myxococcales;  Cystobacterineae; Cystobacteraceae; Cystobacter. | 35 | 0.067 | Identities = 36/117 (31%), Positives = 54/117 (46%), Gaps = 21/117 (18%)  Frame = +1 |
| scaffold_3 | 10152 | 9868 | 241 | hypothetical protein | No significant similarity found. |  |  |  |  | 714 | BGC0000986\|c1\|29429-33760\|+\|no_locus_tag\|nonribosomal_peptide_synthetase\|CAQ34921.1 Length=1443 | Dkxanthene biosynthetic gene cluster | Stigmatella aurantiaca DW4/3-1  Bacteria; Proteobacteria; Deltaproteobacteria; Myxococcales;  Cystobacterineae; Cystobacteraceae; Stigmatella. | 29.3 | 3.6 | Identities = 23/68 (34%), Positives = 32/68 (47%), Gaps = 12/68 (18%)  Frame = +1 |
| scaffold_3 | 11176 | 10154 | 242 | hypothetical protein | ATP-dependent helicase [*Streptomyces kasugaensis*] | 73% | 7.5 | 44% | gi\|1235642535\|WP_094793128.1 | 150 | BGC0000974\|c1\|32700-41591\|+\|no_locus_tag\|non-ribosomal_peptide_synthetase/polyketide_synthase\|CBD77746.1 Length=2963 | Crocacin biosynthetic gene cluster | Chondromyces crocatus  Bacteria; Proteobacteria; Deltaproteobacteria; Myxococcales;  Sorangiineae; Polyangiaceae; Chondromyces. | 27.3 | 0.39 | Identities = 14/36 (39%), Positives = 17/36 (47%), Gaps = 0/36 (0%)  Frame = -1 |
| scaffold_3 | 11562 | 11371 | 243 | hypothetical protein | hypothetical protein [*Thermoanaerobacterium sp. PSU-2*] | 60% | 7e-10 | 50% | gi\|1184544938\|WP_085113370.1 | 309 | BGC0000084\|c1\|3136-5487\|-\|no_locus_tag\|putative_histidine_kinase_sensor\|AFL48547.1 Length=783 | Laidlomycin biosynthetic gene cluster | Streptomyces sp. CS684  Bacteria; Actinobacteria; Actinobacteridae; Actinomycetales;  Streptomycineae; Streptomycetaceae; Streptomyces. | 26.6 | 3.9 | Identities = 21/71 (30%), Positives = 36/71 (51%), Gaps = 6/71 (8%)  Frame = +1 |
| scaffold_3 | 13499 | 11571 | 244 | hypothetical protein | DNA modification methylase [*bacterium*] | 100% | 6.4 | 32% | gi\|1247349913\|PCI11632.1 | 162 | BGC0001392\|c1\|21459-21923\|+\|Cpin_5333\|transcriptional_regulator,_MarR_family\|ACU62764.1 Length=154 | Pinensins biosynthetic gene cluster | Chitinophaga pinensis DSM 2588  Bacteria; Bacteroidetes; Sphingobacteriia; Sphingobacteriales;  Chitinophagaceae; Chitinophaga. | 25.8 | 1.4 | Identities = 15/39 (38%), Positives = 21/39 (54%), Gaps = 4/39 (10%)  Frame = +3 |
| scaffold_3 | 14313 | 13612 | 245 | hypothetical protein | hypothetical protein [*Thermoanaerobacterium sp. PSU-2*] | 89% | 3e-09 | 45% | gi\|1184544938\|WP_085113370.1 | 198 | BGC0001032\|c1\|19887-27098\|+\|no_locus_tag\|polyketide_synthase\|AFX60334.1 Length=2403 | Oocydin A biosynthetic gene cluster | Serratia plymuthica  Bacteria; Proteobacteria; Gammaproteobacteria; Enterobacteriales;  Enterobacteriaceae; Serratia. | 29.3 | 0.16 | Identities = 19/49 (39%), Positives = 28/49 (57%), Gaps = 4/49 (8%)  Frame = +1 |
| scaffold_3 | 14401 | 14532 | 246 | hypothetical protein | hypothetical protein COB51_12200 [*Moraxellaceae bacterium*] | 75% | 9.8 | 42% | gi\|1247382713\|PCI42961.1 | 147 | BGC0000287\|c1\|16513-17400\|-\|PA2310\|hypothetical_protein\|AAG05698.1 Length=295 | 2-amino-4-methoxy-trans-3-butenoic acid biosynthetic gene cluster | Pseudomonas aeruginosa PAO1  Bacteria; Proteobacteria; Gammaproteobacteria; Pseudomonadales;  Pseudomonadaceae; Pseudomonas. | 26.2 | 0.97 | Identities = 15/36 (42%), Positives = 18/36 (50%), Gaps = 2/36 (6%)  Frame = +3 |
| scaffold_3 | 15439 | 14567 | 247 | hypothetical protein | MULTISPECIES: hypothetical protein [*Methanobacterium*] | 86% | 1e-04 | 53% | gi\|1178861314\|WP_083240835.1 | 153 | BGC0001234\|c2\|6621-7538\|-\|AFLA_121510\|hypothetical_protein\|XP_002384858.1 Length=214 | 4,4'-piperazine-2,5-diyldimethyl-bis-phenol biosynthetic gene  cluster | Aspergillus flavus NRRL3357  Eukaryota; Fungi; Dikarya; Ascomycota; Pezizomycotina;  Eurotiomycetes; Eurotiomycetidae; Eurotiales; Aspergillaceae;  Aspergillus. | 25.8 | 1.2 | Identities = 13/38 (34%), Positives = 21/38 (55%), Gaps = 2/38 (5%)  Frame = +1 |
| scaffold_3 | 16367 | 15654 | 248 | hypothetical protein | hypothetical protein CRE_27017 [*Caenorhabditis remanei*] | 82% | 0.86 | 34% | gi\|308504697\|XP_003114532.1 | 312 | BGC0000119\|c1\|63030-63710\|+\|no_locus_tag\|putative_phosphatase\|ACJ24880.1 Length=226 | Pactamycin biosynthetic gene cluster | Streptomyces pactum  Bacteria; Actinobacteria; Actinobacteridae; Actinomycetales;  Streptomycineae; Streptomycetaceae; Streptomyces. | 28.1 | 1.3 | Identities = 15/48 (31%), Positives = 25/48 (52%), Gaps = 2/48 (4%)  Frame = +1 |
| scaffold_3 | 16390 | 16539 | 249 | hypothetical protein | YraN family protein [*Olsenella sp. KH3B4*] | 80% | 0.018 | 28% | gi\|1222960219\|WP_091002376.1 | 396 | BGC0001079\|c1\|18638-20224\|-\|no_locus_tag\|NapH1\|ABS50458.1 Length=528 | Napyradiomycin biosynthetic gene cluster | Streptomyces aculeolatus  Bacteria; Actinobacteria; Actinobacteridae; Actinomycetales;  Streptomycineae; Streptomycetaceae; Streptomyces. | 29.6 | 0.78 | Identities = 16/60 (27%), Positives = 29/60 (48%), Gaps = 7/60 (12%)  Frame = -3 |
| scaffold_3 | 17196 | 16888 | 250 | hypothetical protein | zinc ribbon domain-containing protein [*Selenomonas sp. ND2010*] | 89% | 0.001 | 36% | gi\|697203884\|WP_033169358.1 | 180 | BGC0000708\|c1\|36556-37932\|+\|no_locus_tag\|putative_Fe-S_oxidoreductase\|CAG38716.1 Length=458 | Lividomycin biosynthetic gene cluster | Streptomyces lividus  Bacteria; Actinobacteria; Actinobacteridae; Actinomycetales;  Streptomycineae; Streptomycetaceae; Streptomyces. | 27.3 | 0.58 | Identities = 8/16 (50%), Positives = 12/16 (75%), Gaps = 0/16 (0%)  Frame = +1 |
| scaffold_3 | 17375 | 17214 | 251 | hypothetical protein | No significant similarity found. |  |  |  |  | 114 | BGC0000224\|c1\|31226-32515\|-\|no_locus_tag\|unknown\|AAQ08941.1 Length=429 | Fredericamycin biosynthetic gene cluster | Streptomyces griseus  Bacteria; Actinobacteria; Actinobacteridae; Actinomycetales;  Streptomycineae; Streptomycetaceae; Streptomyces. | 26.2 | 0.59 | Identities = 10/22 (45%), Positives = 14/22 (64%), Gaps = 0/22 (0%)  Frame = +3 |
| scaffold_3 | 18093 | 17896 | 252 | hypothetical protein | MULTISPECIES: DUF2273 domain-containing protein [*Curtobacterium*] | 68% | 6.2 | 46% | gi\|1092405791\|WP_070418041.1 | 126 | BGC0000970\|c1\|22030-26265\|+\|no_locus_tag\|polyketide_synthase\|CAQ43077.1 Length=1411 | Chondrochloren biosynthetic gene cluster | Chondromyces crocatus  Bacteria; Proteobacteria; Deltaproteobacteria; Myxococcales;  Sorangiineae; Polyangiaceae; Chondromyces. | 23.9 | 5.4 | Identities = 11/21 (52%), Positives = 12/21 (57%), Gaps = 0/21 (0%)  Frame = -1 |
| scaffold_5 | 2 | 220 | 253 | DNA-cytosine methyltransferase (EC 2.1.1.37) | DNA (cytosine-5-)-methyltransferase [*uncultured Mediterranean phage*] | 94% | 5e-16 | 60% | gi\|1043236728\|ANS05433.1 | 219 | BGC0000281\|c1\|1-678\|-\|no_locus_tag\|PhlH\|AAY86545.1 Length=225 | 2,4-Diacetylphloroglucinol biosynthetic gene cluster | Pseudomonas fluorescens  Bacteria; Proteobacteria; Gammaproteobacteria; Pseudomonadales;  Pseudomonadaceae; Pseudomonas. | 26.6 | 1.7 | Identities = 12/28 (43%), Positives = 15/28 (54%), Gaps = 0/28 (0%)  Frame = +1 |
| scaffold_5 | 217 | 657 | 254 | Very-short-patch mismatch repair endonuclease (G-T specific) | very short patch repair endonuclease [*Novosphingobium lentum*] | 96% | 4e-26 | 38% | gi\|1180916503\|WP_084355942.1 | 441 | BGC0001016\|c1\|5238-15686\|-\|no_locus_tag\|polyketide_synthase_peptide_sythetase_fusion_protein\|AAO62582.1 Length=3482 | Microcystin biosynthetic gene cluster | Anabaena sp. 90  Bacteria; Cyanobacteria; Nostocales; Nostocaceae; Anabaena. | 29.3 | 1.3 | Identities = 15/37 (41%), Positives = 20/37 (54%), Gaps = 6/37 (16%)  Frame = +1 |
| scaffold_5 | 6187 | 6002 | 255 | hypothetical protein | hypothetical protein VHEMI08971 [*Torrubiella hemipterigena*] | 86% | 0.017 | 43% | gi\|729182218\|CEJ93379.1 | 186 | BGC0000135\|c1\|52185-71039\|+\|no_locus_tag\|polyketide_synthase\|BAK64649.1 Length=6284 | Reveromycin biosynthetic gene cluster | Streptomyces sp. SN-593  Bacteria; Actinobacteria; Actinobacteridae; Actinomycetales;  Streptomycineae; Streptomycetaceae; Streptomyces. | 26.2 | 1.7 | Identities = 9/20 (45%), Positives = 15/20 (75%), Gaps = 0/20 (0%)  Frame = +1 |
| scaffold_5 | 7392 | 7255 | 256 | hypothetical protein | YajQ family cyclic di-GMP-binding protein [*Sphingobacteriaceae bacterium*] | 95% | 2.1 | 40% | gi\|1245776909\|WP_096090649.1 | 138 | BGC0000974\|c1\|32700-41591\|+\|no_locus_tag\|non-ribosomal_peptide_synthetase/polyketide_synthase\|CBD77746.1 Length=2963 | Crocacin biosynthetic gene cluster | Chondromyces crocatus  Bacteria; Proteobacteria; Deltaproteobacteria; Myxococcales;  Sorangiineae; Polyangiaceae; Chondromyces. | 25 | 2.4 | Identities = 11/36 (31%), Positives = 21/36 (58%), Gaps = 0/36 (0%)  Frame = +1 |
| scaffold_5 | 8505 | 8386 | 257 | hypothetical protein | hypothetical protein PPL_01353 [*Polysphondylium pallidum PN500*] | 71% | 4.7 | 46% | gi\|1160553075\|XP_020437677.1 | 120 | BGC0000144\|c1\|69148-74082\|+\|no_locus_tag\|polyketide_synthase\|AEZ53951.1 Length=1644 | Salinomycin biosynthetic gene cluster | Streptomyces albus  Bacteria; Actinobacteria; Actinobacteridae; Actinomycetales;  Streptomycineae; Streptomycetaceae; Streptomyces. | 24.6 | 2.3 | Identities = 11/20 (55%), Positives = 13/20 (65%), Gaps = 0/20 (0%)  Frame = +1 |
| scaffold_6 | 563 | 742 | 258 | hypothetical protein | OmpA/MotB domain-containing protein [*gamma proteobacterium BDW918*] | 79% | 1.8 | 30% | gi\|385277666\|EIF41644.1 | 390 | BGC0000109\|c1\|1013-3781\|-\|no_locus_tag\|transcriptional_regulatory_protein\|BAF85834.1 Length=922 | Nemadectin biosynthetic gene cluster | Streptomyces cyaneogriseus subsp. noncyanogenus  Bacteria; Actinobacteria; Actinobacteridae; Actinomycetales;  Streptomycineae; Streptomycetaceae; Streptomyces. | 29.6 | 0.64 | Identities = 27/99 (27%), Positives = 39/99 (39%), Gaps = 5/99 (5%)  Frame = +1 |
| scaffold_6 | 1066 | 1635 | 259 | hypothetical protein | siderophore-interacting protein [*Kibdelosporangium aridum*] | 89% | 8.8 | 40% | gi\|1181018103\|WP_084431148.1 | 120 | BGC0000182\|c1\|600-1709\|+\|no_locus_tag\|MupA\|AAK28503.1 Length=369 | Mupirocin biosynthetic gene cluster | Pseudomonas fluorescens  Bacteria; Proteobacteria; Gammaproteobacteria; Pseudomonadales;  Pseudomonadaceae; Pseudomonas. | 31.6 | 0.007 | Identities = 13/38 (34%), Positives = 20/38 (53%), Gaps = 0/38 (0%)  Frame = -3 |
| scaffold_6 | 2420 | 2722 | 260 | hypothetical protein | glycosyl hydrolases family 35 protein [*Toxoplasma gondii GAB2-2007-GAL-DOM2*] | 64% | 1.3 | 30% | gi\|672257451\|KFG32666.1 | 453 | BGC0000387\|c1\|1-472\|+\|no_locus_tag\|HAD-superfamily_hydrolase_subfamily_IA_variant_3\|ABG00041.1 Length=156 | Mangotoxin biosynthetic gene cluster | Pseudomonas syringae pv. syringae  Bacteria; Proteobacteria; Gammaproteobacteria; Pseudomonadales;  Pseudomonadaceae; Pseudomonas; Pseudomonas syringae. | 28.9 | 1.3 | Identities = 15/46 (33%), Positives = 24/46 (52%), Gaps = 1/46 (2%)  Frame = +1 |
| scaffold_6 | 2933 | 2760 | 261 | hypothetical protein | Vi polysaccharide biosynthesis UDP-N-acetylglucosamine C-6 dehydrogenase TviB [*Kiritimatiella glycovorans*] | 92% | 6.7 | 40% | gi\|919584995\|WP_052882757.1 | 165 | BGC0000288\|c1\|62785-65541\|-\|no_locus_tag\|putative_phosphoenolpyruvate_synthase\|BAJ19075.1 Length=918 | A-503083 biosynthetic gene cluster | Streptomyces sp. SANK 62799  Bacteria; Actinobacteria; Actinobacteridae; Actinomycetales;  Streptomycineae; Streptomycetaceae; Streptomyces. | 28.5 | 0.16 | Identities = 13/28 (46%), Positives = 17/28 (61%), Gaps = 0/28 (0%)  Frame = +1 |
| scaffold_6 | 3086 | 3274 | 262 | hypothetical protein | aminotransferase [*Methylothermaceae bacteria B42*] | 66% | 1.6 | 39% | gi\|1000063597\|KXJ40911.1 | 201 | BGC0001381\|c1\|168926-170152\|-\|no_locus_tag\|Arene(phthalate)_dioxygenase\|AJO72761.1 Length=408 | Brasilinolide biosynthetic gene cluster | Nocardia terpenica  Bacteria; Actinobacteria; Corynebacteriales; Nocardiaceae; Nocardia. | 26.6 | 1.5 | Identities = 15/43 (35%), Positives = 26/43 (60%), Gaps = 1/43 (2%)  Frame = +1 |
| scaffold_6 | 5275 | 4877 | 263 | hypothetical protein | hypothetical protein FF38_07955 [*Lucilia cuprina*] | 77% | 8.5 | 35% | gi\|906464953\|KNC26685.1 | 234 | BGC0001055\|c1\|39966-41066\|+\|eco2026\|hypothetical_protein\|CAJ87592.1 Length=366 | Yersiniabactin biosynthetic gene cluster | Escherichia coli  Bacteria; Proteobacteria; Gammaproteobacteria; Enterobacteriales;  Enterobacteriaceae; Escherichia. | 25.8 | 3.7 | Identities = 12/30 (40%), Positives = 16/30 (53%), Gaps = 0/30 (0%)  Frame = +3 |
| scaffold_6 | 6082 | 5498 | 264 | hypothetical protein | Putative multidrug export ATP-binding/permease protein SAV1866 [*Collinsella aerofaciens*] | 78% | 1.1 | 44% | gi\|932898031\|CUN69282.1 | 126 | BGC0000460\|c1\|14425-15240\|-\|VV2_0837\|Vulnibactin_utilization_protein_VuuB\|AAO07760.1 Length=271 | Vulnibactin biosynthetic gene cluster | Vibrio vulnificus CMCP6  Bacteria; Proteobacteria; Gammaproteobacteria; Vibrionales;  Vibrionaceae; Vibrio. | 26.2 | 0.8 | Identities = 12/35 (34%), Positives = 19/35 (54%), Gaps = 0/35 (0%)  Frame = -1 |
| scaffold_6 | 6627 | 6082 | 265 | polysaccharide deacetylase | DNA phosphorothioation-dependent restriction protein DptH [*Aliivibrio fischeri*] | 93% | 3.3 | 33% | gi\|1067267396\|OED51110.1 | 180 | BGC0000806\|c1\|59053-59571\|-\|no_locus_tag\|anti-sigma_factor\|AHL24492.1 Length=172 | Phosphonoglycans biosynthetic gene cluster | Glycomyces sp. NRRL B-16210  Bacteria; Actinobacteria; Actinobacteridae; Actinomycetales;  Glycomycineae; Glycomycetaceae; Glycomyces. | 25.4 | 2.7 | Identities = 10/29 (34%), Positives = 14/29 (48%), Gaps = 0/29 (0%)  Frame = -1 |
| scaffold_6 | 7002 | 7121 | 266 | hypothetical protein | copper amine oxidase N-terminal domain protein [*Peptoniphilus harei ACS-146-V-Sch2b*] | 98% | 0.40 | 24% | gi\|312845763\|EFR33152.1 | 570 | BGC0000133\|c1\|77436-79640\|-\|no_locus_tag\|exodeoxyribonuclease\|AFI57030.1 Length=734 | Quartromicin biosynthetic gene cluster | Amycolatopsis orientalis  Bacteria; Actinobacteria; Actinobacteridae; Actinomycetales;  Pseudonocardineae; Pseudonocardiaceae; Amycolatopsis. | 29.6 | 1.8 | Identities = 19/51 (37%), Positives = 28/51 (55%), Gaps = 4/51 (8%)  Frame = +1 |
| scaffold_6 | 7169 | 8230 | 267 | peptidase S8 and S53, subtilisin, kexin, sedolisin | bleomycin hydrolase [*Drosophila obscura*] | 60% | 1.5 | 33% | gi\|1236498301\|XP_022227169.1 | 303 | BGC0000154\|c1\|24057-25227\|-\|TSTA_117800\|1-aminocyclopropane-1-carboxylate_oxidase,_putative\|EED18007.1 Length=325 | Stipitatic acid biosynthetic gene cluster | Talaromyces stipitatus ATCC 10500  Eukaryota; Fungi; Dikarya; Ascomycota; Pezizomycotina;  Eurotiomycetes; Eurotiomycetidae; Eurotiales; Trichocomaceae;  Talaromyces. | 29.3 | 0.5 | Identities = 21/62 (34%), Positives = 32/62 (52%), Gaps = 6/62 (10%)  Frame = +1 |
| scaffold_6 | 8563 | 8775 | 268 | hypothetical protein | CopG family transcriptional regulator [*Natronococcus occultus*] | 84% | 0.30 | 44% | gi\|505136118\|WP_015323220.1 | 174 | GC0001373\|c1\|49874-51434\|-\|no_locus_tag\|LysR_family_regulatory_protein\|BAV32157.1 Length=499 | Sordarin biosynthetic gene cluster | Sordaria araneosa  Eukaryota; Fungi; Dikarya; Ascomycota; Pezizomycotina;  Sordariomycetes; Sordariomycetidae; Sordariales; Sordariaceae;  Sordaria. | 24.6 | 5.7 | Identities = 9/25 (36%), Positives = 15/25 (60%), Gaps = 0/25 (0%)  Frame = +3 |
| scaffold_6 | 9893 | 10195 | 269 | hypothetical protein | hypothetical protein Egran_04870 [*Elaphomyces granulatus*] | 88% | 1.4 | 33% | gi\|1227549077\|OXV07365.1 | 189 | BGC0001069\|c1\|5072-22603\|+\|no_locus_tag\|polyketide_synthase\|AAY89049.1 Length=5843 | Chivosazole biosynthetic gene cluster | Sorangium cellulosum  Bacteria; Proteobacteria; Deltaproteobacteria; Myxococcales;  Sorangiineae; Polyangiaceae; Sorangium. | 25.4 | 3 | Identities = 13/51 (25%), Positives = 26/51 (51%), Gaps = 0/51 (0%)  Frame = -2 |
| scaffold_6 | 11668 | 11279 | 270 | hypothetical protein | metallophosphoesterase [*Aquifex aeolicus*] | 85% | 2e-05 | 31% | gi\|499182867\|WP_010880407.1 | 399 | BGC0000404\|c1\|23318-34654\|-\|no_locus_tag\|alpha-aminoadypil-cysteinyl-valine_synthetase\|ABA70582.1 Length=3778 | Penicillin biosynthetic gene cluster | Penicillium chrysogenum  Eukaryota; Fungi; Dikarya; Ascomycota; Pezizomycotina;  Eurotiomycetes; Eurotiomycetidae; Eurotiales; Aspergillaceae;  Penicillium; Penicillium chrysogenum complex. | 29.6 | 0.83 | Identities = 11/33 (33%), Positives = 17/33 (52%), Gaps = 0/33 (0%)  Frame = -2 |
| scaffold_6 | 11994 | 11875 | 271 | hypothetical protein | O-methyltransferase [*Bacteroides nordii*] | 41% | 0.89 | 38% | gi\|494747424\|WP_007482832.1 | 585 | BGC0001205\|c1\|21257-28797\|+\|PEXP_030540\|Acyl_transferase/acyl_hydrolase/lysophospholipase\|KGO40478.1 Length=2363 | Communesin biosynthetic gene cluster | Penicillium expansum  Eukaryota; Fungi; Dikarya; Ascomycota; Pezizomycotina;  Eurotiomycetes; Eurotiomycetidae; Eurotiales; Aspergillaceae;  Penicillium. | 37 | 0.07 | Identities = 23/70 (33%), Positives = 33/70 (47%), Gaps = 1/70 (1%)  Frame = +1 |
| scaffold_6 | 12443 | 11991 | 272 | hypothetical protein | family 2 glycosyl transferase [*Halalkalicoccus jeotgali*] | 98% | 2e-15 | 34% | gi\|495691397\|WP_008415976.1 | 546 | BGC0000644\|c1\|5545-6276\|-\|no_locus_tag\|glucosyl_transferase\|ABD24403.1 Length=243 | Carotenoid biosynthetic gene cluster | Dietzia sp. CQ4  Bacteria; Actinobacteria; Actinobacteridae; Actinomycetales;  Corynebacterineae; Dietziaceae; Dietzia. | 48.5 | 4.00E-07 | Identities = 47/152 (31%), Positives = 72/152 (47%), Gaps = 17/152 (11%)  Frame = +1 |
| scaffold_6 | 12612 | 12448 | 273 | hypothetical protein | hypothetical protein [*Bifidobacterium sp. TRE1*] | 94% | 0.38 | 43% | gi\|1285194360\|WP_100510292.1 | 120 | BGC0001381\|c1\|198311-199093\|+\|no_locus_tag\|hypothetical_protein\|AJO72785.1 Length=260 | Brasilinolide biosynthetic gene cluster | Nocardia terpenica  Bacteria; Actinobacteria; Corynebacteriales; Nocardiaceae; Nocardia. | 24.6 | 2.1 | Identities = 13/28 (46%), Positives = 16/28 (57%), Gaps = 0/28 (0%)  Frame = +1 |
| scaffold_6 | 13588 | 13388 | 274 | hypothetical protein | serine protease AprX [*Bradyrhizobium erythrophlei*] | 79% | 6e-26 | 34% | gi\|1119071599\|SIO25423.1 | 1062 | BGC0000595\|c1\|9346-13008\|-\|SCO2446\|peptidase\|NP_626689.1 Length=1220 | SCO-2138 biosynthetic gene cluster | Streptomyces coelicolor A3(2)  Bacteria; Actinobacteria; Actinobacteridae; Actinomycetales;  Streptomycineae; Streptomycetaceae; Streptomyces; Streptomyces  albidoflavus group. | 47.4 | 1.00E-05 | Identities = 54/201 (27%), Positives = 86/201 (43%), Gaps = 21/201 (10%)  Frame = +1 |
| scaffold_6 | 16335 | 16568 | 275 | hypothetical protein | thiol reductant ABC exporter subunit CydD [*Paenibacillus apiarius*] | 88% | 0.29 | 30% | gi\|1199704204\|WP_087434714.1 | 213 | BGC0001019\|c1\|16327-20982\|+\|no_locus_tag\|polyketide_synthase\|AHB82053.1 Length=1551 | Microsclerodermins biosynthetic gene cluster | Sorangium cellulosum  Bacteria; Proteobacteria; Deltaproteobacteria; Myxococcales;  Sorangiineae; Polyangiaceae; Sorangium. | 25 | 5.7 | Identities = 15/34 (44%), Positives = 16/34 (47%), Gaps = 0/34 (0%)  Frame = +1 |
| scaffold_6 | 16950 | 17075 | 276 | hypothetical protein | cell surface glycoprotein [*Natronomonas pharaonis*] | 86% | 0.008 | 33% | gi\|499643284\|WP_011324018.1 | 303 | BGC0000206\|c1\|5148-5933\|-\|no_locus_tag\|ChaD_protein\|CAH10157.1 Length=261 | Chartreusin biosynthetic gene cluster | Streptomyces chartreusis  Bacteria; Actinobacteria; Actinobacteridae; Actinomycetales;  Streptomycineae; Streptomycetaceae; Streptomyces. | 29.3 | 0.46 | Identities = 9/25 (36%), Positives = 19/25 (76%), Gaps = 0/25 (0%)  Frame = +1 |
| scaffold_7 | 470 | 868 | 277 | hypothetical protein | hypothetical protein BA874_06665 [*Desulfuromonadales bacterium C00003068*] | 90% | 3e-13 | 36% | gi\|1072877864\|OEU76116.1 | 399 | BGC0000952\|c2\|16107-16745\|+\|no_locus_tag\|putative_dTDP-4-deoxyglucose_3,5-epimerase\|CBW45689.1 Length=212 | Pristinamycin biosynthetic gene cluster | Streptomyces pristinaespiralis  Bacteria; Actinobacteria; Actinobacteridae; Actinomycetales;  Streptomycineae; Streptomycetaceae; Streptomyces. | 27.3 | 3.5 | Identities = 29/99 (29%), Positives = 45/99 (45%), Gaps = 14/99 (14%)  Frame = +1 |
| scaffold_7 | 1010 | 1150 | 278 | hypothetical protein | cytoplasmic protein [*Deltaproteobacteria bacterium RBG_13_53_10*] | 91% | 0.11 | 38% | gi\|1084724869\|OGP69060.1 | 141 | BGC0000905\|c1\|10065-11054\|+\|no_locus_tag\|delta-aminolevulinic_acid_dehydrotase/porphobilinogen_synthase\|ABT18045.1 Length=329 | Heme D1 biosynthetic gene cluster | Heliobacillus mobilis  Bacteria; Firmicutes; Clostridia; Clostridiales; Heliobacteriaceae;  Heliobacillus. | 26.6 | 0.7 | Identities = 12/37 (32%), Positives = 18/37 (49%), Gaps = 0/37 (0%)  Frame = +1 |
| scaffold_7 | 1802 | 2440 | 279 | Transcription initiation factor B | transcription initiation factor IIB [*candidate divison MSBL1 archaeon SCGC-AAA259D14*] | 70% | 2e-57 | 64% | gi\|985656859\|KXA89394.1 | 639 | BGC0000236\|c1\|13840-16272\|+\|no_locus_tag\|unknown\|AAO65338.1 Length=810 | Kinamycin biosynthetic gene cluster | Streptomyces murayamaensis  Bacteria; Actinobacteria; Actinobacteridae; Actinomycetales;  Streptomycineae; Streptomycetaceae; Streptomyces. | 32.3 | 0.25 | Identities = 25/74 (34%), Positives = 39/74 (53%), Gaps = 5/74 (7%)  Frame = +1 |
| scaffold_7 | 2498 | 2632 | 280 | hypothetical protein | membrane protein [*uncultured organism*] | 79% | 0.001 | 51% | gi\|452077110\|AGF93079.1 | 135 | BGC0001182\|c1\|27466-31627\|-\|CHGG_01242\|hypothetical_protein\|XP_001220463.1 Length=1090 | Chaetoglobosins biosynthetic gene cluster | Chaetomium globosum CBS 148.51  Eukaryota; Fungi; Dikarya; Ascomycota; Pezizomycotina;  Sordariomycetes; Sordariomycetidae; Sordariales; Chaetomiaceae;  Chaetomium. | 25.8 | 1 | Identities = 11/28 (39%), Positives = 17/28 (61%), Gaps = 0/28 (0%)  Frame = +3 |
| scaffold_7 | 2821 | 2985 | 281 | hypothetical protein | hypothetical protein AKJ66_01420 [*candidate divison MSBL1 archaeon SCGC-AAA259E22*] | 100% | 2e-15 | 57% | gi\|985661700\|KXA93684.1 | 165 | BGC0000446\|c1\|17602-21972\|+\|no_locus_tag\|non-ribosomal_peptide_synthetase,_terminal_component\|CDG76959.1 Length=1456 | Tilivalline biosynthetic gene cluster | Klebsiella oxytoca  Bacteria; Proteobacteria; Gammaproteobacteria; Enterobacteriales;  Enterobacteriaceae; Klebsiella. | 26.9 | 0.62 | Identities = 11/23 (48%), Positives = 15/23 (65%), Gaps = 0/23 (0%)  Frame = -1 |
| scaffold_7 | 3446 | 2982 | 282 | hypothetical protein | ABC transporter ATP-binding protein [*Bacillus atrophaeus*] | 44% | 0.048 | 35% | gi\|1229386581\|WP_094231484.1 | 465 | BGC0000055\|c1\|32514-42029\|+\|SACE_0724\|EryAIII_Erythromycin_polyketide_synthase_modules_5_and_6\|CAM00065.1 Length=3171 | Erythromycin biosynthetic gene cluster | Saccharopolyspora erythraea NRRL 2338  Bacteria; Actinobacteria; Actinobacteridae; Actinomycetales;  Pseudonocardineae; Pseudonocardiaceae; Saccharopolyspora. | 28.1 | 4 | Identities = 24/71 (34%), Positives = 35/71 (49%), Gaps = 12/71 (17%)  Frame = +1 |
| scaffold_7 | 3577 | 3443 | 283 | hypothetical protein | DNA topoisomerase IV subunit A [*Thalassobius maritimus*] | 90% | 2.3 | 43% | gi\|1119906886\|WP_072791899.1 | 135 | BGC0001347\|c2\|2639-15838\|+\|no_locus_tag\|non-ribosomal_peptide_synthetase\|ANQ43348.1 Length=4399 | Poaeamide B biosynthetic gene cluster | Pseudomonas synxantha  Bacteria; Proteobacteria; Gammaproteobacteria; Pseudomonadales;  Pseudomonadaceae; Pseudomonas. | 26.2 | 0.7 | Identities = 14/32 (44%), Positives = 17/32 (53%), Gaps = 0/32 (0%)  Frame = -1 |
| scaffold_7 | 3679 | 4077 | 284 | hypothetical protein | hypothetical protein [*Alkalibacterium subtropicum*] | 74% | 0.001 | 36% | gi\|1223492678\|WP_091529977.1 | 399 | BGC0001034\|c1\|51618-58979\|+\|no_locus_tag\|polyketide_synthase\|CCE88378.1 Length=2453 | Pellasoren biosynthetic gene cluster | Sorangium cellulosum  Bacteria; Proteobacteria; Deltaproteobacteria; Myxococcales;  Sorangiineae; Polyangiaceae; Sorangium. | 26.9 | 5.6 | Identities = 19/50 (38%), Positives = 28/50 (56%), Gaps = 4/50 (8%)  Frame = -1 |
| scaffold_7 | 4371 | 4084 | 285 | hypothetical protein | PREDICTED: coiled-coil domain-containing protein 186-like isoform X2 [*Branchiostoma belcheri*] | 88% | 0.55 | 33% | gi\|1126187113\|XP_019626303.1 | 288 | BGC0001178\|c1\|1135-2268\|-\|no_locus_tag\|Integral_membrane_sensor_kinase\|AGS77301.1 Length=377 | UK-68,597 biosynthetic gene cluster | Actinoplanes sp. ATCC 53533  Bacteria; Actinobacteria; Actinobacteridae; Actinomycetales;  Micromonosporineae; Micromonosporaceae; Actinoplanes. | 26.9 | 2.7 | Identities = 12/32 (38%), Positives = 21/32 (66%), Gaps = 2/32 (6%)  Frame = +1 |
| scaffold_8 | 317 | 3 | 286 | hypothetical protein | sulfatase [*Halorubrum distributum JCM 9100*] | 85% | 0.001 | 30% | gi\|445693463\|ELZ45611.1 | 315 | BGC0001342\|c1\|17602-36369\|+\|no_locus_tag\|TugA\|ADH04657.1 Length=6255 | Thuggacin biosynthetic gene cluster | Chondromyces crocatus  Bacteria; Proteobacteria; Deltaproteobacteria; Myxococcales;  Sorangiineae; Polyangiaceae; Chondromyces. | 26.9 | 3.6 | Identities = 11/27 (41%), Positives = 15/27 (56%), Gaps = 0/27 (0%)  Frame = -1 |
| scaffold_8 | 424 | 981 | 287 | Mannosyltransferase | glycogen synthase GlgA [*Thermococcus barophilus*] | 99% | 3e-45 | 42% | gi\|948744483\|WP_056934594.1 | 558 | BGC0000808\|c2\|4303-6768\|-\|Bmul_4608\|glycosyl_transferase_group_1\|ABX18286.1 Length=821 | Cepacian biosynthetic gene cluster | Burkholderia multivorans ATCC 17616  Bacteria; Proteobacteria; Betaproteobacteria; Burkholderiales;  Burkholderiaceae; Burkholderia; Burkholderia cepacia complex. | 72.8 | 4.00E-15 | Identities = 50/185 (27%), Positives = 90/185 (49%), Gaps = 20/185 (11%)  Frame = +1 |
| scaffold_8 | 1493 | 1116 | 288 | hypothetical protein | hypothetical protein B6U80_00770 [*Candidatus Pacearchaeota archaeon ex4484_26*] | 62% | 0.14 | 28% | gi\|1231979524\|OYT41719.1 | 378 | BGC0001030\|c1\|3246-5384\|-\|no_locus_tag\|ochratoxin_A_non-ribosomal_peptide_synthetase\|AAS98174.1 Length=712 | Ochratoxin A biosynthetic gene cluster | Penicillium nordicum  Eukaryota; Fungi; Dikarya; Ascomycota; Pezizomycotina;  Eurotiomycetes; Eurotiomycetidae; Eurotiales; Aspergillaceae;  Penicillium. | 32 | 0.11 | Identities = 18/52 (35%), Positives = 24/52 (46%), Gaps = 3/52 (6%)  Frame = -1 |
| scaffold_8 | 2634 | 2518 | 289 | hypothetical protein | No significant similarity found. |  |  |  |  | 117 | BGC0000447\|c1\|66992-68950\|+\|no_locus_tag\|macrolide_export_ATP-binding/permease_protein_MacB\|CCJ67642.1 Length=652 | Tolaasin biosynthetic gene cluster | Pseudomonas costantinii  Bacteria; Proteobacteria; Gammaproteobacteria; Pseudomonadales;  Pseudomonadaceae; Pseudomonas. | 25.4 | 1.2 | Identities = 9/27 (33%), Positives = 16/27 (59%), Gaps = 0/27 (0%)  Frame = -2 |

**Table S2:** **Annotation of all PEGs on 10-2G.** Three annotation tools were used: RAST (Aziz et al., 2008), psi-BLAST (Altschul et al., 1997) and BLASTX against MIBiG curated sequences (Medema et al., 2015) are depicted. Shaded in grey are PEGs with psi-BLAST hits of E-value > 0.005.

| **Scaffold** | **Start** | **Stop** | **PEG** | **RAST annotation** | **Best hit from psi-BLAST** | **Query coverage** | **E-value** | **Identity %** | **Accession number** | **PEG length (bp)** | **MIBIG hit** | **BGC reference in MIBiG** | **Organism** | **Score** | **E-Value** | **Other** |
| --- | --- | --- | --- | --- | --- | --- | --- | --- | --- | --- | --- | --- | --- | --- | --- | --- |
| scaffold_3 | 23 | 205 | 2 | hypothetical protein | hypothetical protein FGRA07_11723 [*Fusarium graminearum*] | 91% | 6.4 | 31% | gi\|1246313629\|PCD21253.1 | 183 | BGC0000372\|c1\|20339-22264\|-\|AFUA_3G12960\|cytochrome_P450_monooxigenase_GliC-like,_putative\|EAL92287.1 Length=550 | Hexadehydro-astechrome (HAS) biosynthetic gene cluster | Aspergillus fumigatus Af293  Eukaryota; Fungi; Dikarya; Ascomycota; Pezizomycotina;  Eurotiomycetes; Eurotiomycetidae; Eurotiales; Aspergillaceae;  Aspergillus. | 26.2 | 1.5 | Identities = 11/25 (44%), Positives = 14/25 (56%), Gaps = 0/25 (0%)  Frame = +3 |
| scaffold_3 | 202 | 702 | 3 | hypothetical protein | class I SAM-dependent methyltransferase [*Methanosarcina mazei*] | 78% | 4e-12 | 34% | gi\|850504616\|WP_048046626.1 | 501 | BGC0000826\|c1\|8672-9571\|-\|SCLAV_p1114\|Staurosporine_biosynthesis_methyltransferase_StaMA\|EFG04600.1 Length=299 | Staurosporine biosynthetic gene cluster | Streptomyces clavuligerus ATCC 27064  Bacteria; Actinobacteria; Actinobacteridae; Actinomycetales;  Streptomycineae; Streptomycetaceae; Streptomyces. | 43.5 | 2.00E-05 | Identities = 22/55 (40%), Positives = 28/55 (51%), Gaps = 3/55 (5%)  Frame = +1 |
| scaffold_3 | 686 | 880 | 4 | hypothetical protein | DUF1415 domain-containing protein [*Marinospirillum minutulum*] | 68% | 0.27 | 41% | gi\|916561110\|WP_051168201.1 | 195 | BGC0001195\|c1\|16348-35577\|+\|no_locus_tag\|type_I_modular_polyketide_synthase\|AJW65407.1 Length=6409 | Nocardiopsin biosynthetic gene cluster | Nocardiopsis sp. CMB-M0232  Bacteria; Actinobacteria; Streptosporangiales; Nocardiopsaceae;  Nocardiopsis. | 26.9 | 1.1 | Identities = 10/23 (43%), Positives = 14/23 (61%), Gaps = 0/23 (0%)  Frame = -3 |
| scaffold_3 | 914 | 1927 | 5 | UDP-glucose 4-epimerase (EC 5.1.3.2) | hypothetical protein AUG75_21995 [*Cyanobacteria bacterium 13_1_20CM_4_61_6*] | 97% | 3e-38 | 31% | gi\|1125591494\|OLE96316.1 | 1014 | BGC0000112\|c1\|17107-18102\|-\|no_locus_tag\|NDP-hexose-4,6-dehydratase\|AAM77990.1 Length=331 | Neocarzinostatin biosynthetic gene cluster | Streptomyces carzinostaticus subsp. neocarzinostaticus  Bacteria; Actinobacteria; Actinobacteridae; Actinomycetales;  Streptomycineae; Streptomycetaceae; Streptomyces. | 64.7 | 1.00E-11 | Identities = 62/230 (27%), Positives = 104/230 (45%), Gaps = 17/230 (7%)  Frame = +1 |
| scaffold_3 | 2297 | 2989 | 6 | Glycosyltransferase | glycosyl transferase [*Parcubacteria group bacterium CG11_big_fil_rev_8_21_14_0_20_39_14*] | 77% | 5e-35 | 38% | gi\|1277086632\|PIQ92058.1 | 693 | BGC0000795\|c1\|26137-27270\|+\|no_locus_tag\|putative_glycosyltransferase\|AAS55731.1 Length=377 | S-layer glycan biosynthetic gene cluster | Aneurinibacillus thermoaerophilus  Bacteria; Firmicutes; Bacilli; Bacillales; Paenibacillaceae;  Aneurinibacillus group; Aneurinibacillus. | 56.6 | 3.00E-09 | Identities = 50/156 (32%), Positives = 78/156 (50%), Gaps = 17/156 (11%)  Frame = +1 |
| scaffold_3 | 2961 | 3542 | 7 | hypothetical protein | ABC transporter permease [*Hymenobacter sp. CRA2*] | 47% | 0.73 | 27% | gi\|1150819492\|WP_078012825.1 | 582 | BGC0000443\|c1\|1300-2094\|+\|no_locus_tag\|AraC_family_transcriptional_regulator\|AED89998.1 Length=264 | Thanamycin biosynthetic gene cluster | Bacteria; Proteobacteria; Gammaproteobacteria; Pseudomonadales;  Pseudomonadaceae; Pseudomonas. | 28.9 | 2.6 | Identities = 12/34 (35%), Positives = 20/34 (59%), Gaps = 0/34 (0%)  Frame = +1 |
| scaffold_3 | 3543 | 4841 | 8 | hypothetical protein | hypothetical protein COW64_06170 [*bacterium (Candidatus Blackallbacteria) CG18_big_fil_WC_8_21_14_2_50_49_26*] | 44% | 4e-11 | 31% | gi\|1277017872\|PIQ27915.1 | 1299 | BGC0000768\|c1\|29537-30166\|-\|no_locus_tag\|conserved_hypothetical_protein\|AAP57703.1 Length=209 | Gellan polysaccharide biosynthetic gene cluster | Sphingomonas elodea ATCC 31461  Bacteria; Proteobacteria; Alphaproteobacteria; Sphingomonadales;  Sphingomonadaceae; Sphingomonas. | 32.3 | 0.44 | Identities = 21/67 (31%), Positives = 30/67 (45%), Gaps = 4/67 (6%)  Frame = -2 |
| scaffold_3 | 4852 | 5544 | 9 | hypothetical protein | Chitinase A precursor [*Phycisphaerae bacterium ST-NAGAB-D1*] | 75% | 3e-17 | 34% | gi\|1150420780\|AQT69446.1 | 693 | BGC0000087\|c1\|101038-104343\|+\|no_locus_tag\|glycoside_hydrolase_family_2,_sugar_binding\|CAQ64717.1 Length=1101 | Lasalocid biosynthetic gene cluster | Bacteria; Actinobacteria; Actinobacteridae; Actinomycetales;  Streptomycineae; Streptomycetaceae; Streptomyces. | 38.9 | 0.003 | Identities = 42/138 (30%), Positives = 61/138 (44%), Gaps = 18/138 (13%)  Frame = +1 |
| scaffold_3 | 5635 | 6057 | 10 | hypothetical protein | No significant similarity found. |  |  |  |  | 423 | BGC0001352\|c1\|38433-40178\|-\|no_locus_tag\|3-hydroxyacyl-CoA_dehydrogenase\|CUI25707.1 Length=581 | Thiotetronate Tü 3010 biosynthetic gene cluster | Streptomyces olivaceus  Bacteria; Actinobacteria; Streptomycetales; Streptomycetaceae;  Streptomyces. | 32.7 | 0.078 | Identities = 17/34 (50%), Positives = 19/34 (56%), Gaps = 0/34 (0%)  Frame = +1 |
| scaffold_3 | 6060 | 6185 | 11 | hypothetical protein | DNA-formamidopyrimidine glycosylase [*candidate division WWE3 bacterium CG10_big_fil_rev_8_21_14_0_10_48_23*] | 82% | 0.018 | 44% | gi\|1279207166\|PJE50460.1 | 126 | BGC0000828\|c1\|1883-4879\|+\|no_locus_tag\|Vio_B\|BAA84783.1 | Violacein biosynthetic gene cluster | Chromobacterium violaceum  Bacteria; Proteobacteria; Betaproteobacteria; Neisseriales;  Chromobacteriaceae; Chromobacterium. | 30 | 0.032 | Identities = 10/29 (34%), Positives = 19/29 (66%), Gaps = 0/29 (0%)  Frame = +2 |
| scaffold_3 | 7157 | 6252 | 12 | hypothetical protein | hypothetical protein [*Vaginella massiliensis*] | 57% | 4e-04 | 25% | gi\|1057226955\|WP_068599341.1 | 906 | BGC0000833\|c1\|8328-9917\|+\|no_locus_tag\|putative_ligase\|AAG29784.2 Length=529 | Coumermycin A1 biosynthetic gene cluster | Streptomyces rishiriensis  Bacteria; Actinobacteria; Actinobacteridae; Actinomycetales;  Streptomycineae; Streptomycetaceae; Streptomyces. | 32.7 | 0.32 | Identities = 25/91 (27%), Positives = 36/91 (40%), Gaps = 19/91 (21%)  Frame = +1 |
| scaffold_3 | 7290 | 7147 | 13 | hypothetical protein | AbrB/MazE/SpoVT family DNA-binding domain-containing protein [*Halorubrum lipolyticum*] | 76% | 0.002 | 58% | gi\|495280090\|WP_008004844.1 | 144 | BGC0000011\|c1\|51698-52555\|-\|no_locus_tag\|AflI\|ACH72897.1 Length=285 | Aflatoxin/sterigmatocystin biosynthetic gene cluster | Aspergillus ochraceoroseus  Eukaryota; Fungi; Dikarya; Ascomycota; Pezizomycotina;  Eurotiomycetes; Eurotiomycetidae; Eurotiales; Aspergillaceae;  Aspergillus. | 30.4 | 0.031 | Identities = 17/43 (40%), Positives = 24/43 (56%), Gaps = 4/43 (9%)  Frame = -1 |
| scaffold_3 | 7490 | 8065 | 14 | Probable hemagglutinin/hemolysin-related protein | hypothetical protein COW46_01675 [*Candidatus Gracilibacteria bacterium CG17_big_fil_post_rev_8_21_14_2_50_48_13*] | 85% | 8e-18 | 34% | gi\|1278001473\|PIV90681.1 | 576 | BGC0000218\|c1\|3258-4517\|+\|no_locus_tag\|daunorubicin-doxorubicin_polyketide_synthase\|AAA65206.1 Length=419 | Doxorubicin biosynthetic gene cluster | Streptomyces peucetius  Bacteria; Actinobacteria; Actinobacteridae; Actinomycetales;  Streptomycineae; Streptomycetaceae; Streptomyces. | 27.7 | 6.1 | Identities = 12/36 (33%), Positives = 18/36 (50%), Gaps = 0/36 (0%)  Frame = +2 |
| scaffold_3 | 8135 | 8314 | 15 | hypothetical protein | hypothetical protein [*Shewanella sp. SACH*] | 83% | 2.5 | 36% | gi\|1110713626\|WP_071939500.1 | 180 | BGC0000414\|c1\|18539-19573\|+\|no_locus_tag\|3-oxoacyl-(acyl-carrier-protein_(acp))_synthase_III_domain-containing_protein\|BAO84859.1 Length=344 | Quinocarcin biosynthetic gene cluster | Streptomyces melanovinaceus  Bacteria; Actinobacteria; Actinobacteridae; Actinomycetales;  Streptomycineae; Streptomycetaceae; Streptomyces. | 24.3 | 7 | Identities = 11/32 (34%), Positives = 19/32 (59%), Gaps = 2/32 (6%)  Frame = -2 |
| scaffold_3 | 8350 | 8754 | 16 | hypothetical protein | major surface protein 3 [*Anaplasma centrale*] | 82% | 0.002 | 33% | gi\|46451427\|AAS97958.1 | 405 | BGC0000533\|c1\|205-1059\|+\|no_locus_tag\|MutR\|AAD56141.1 Length=284 | Mutacin III biosynthetic gene cluster | Streptococcus mutans  Bacteria; Firmicutes; Bacilli; Lactobacillales; Streptococcaceae;  Streptococcus. | 27.3 | 3.6 | Identities = 14/39 (36%), Positives = 23/39 (59%), Gaps = 1/39 (3%)  Frame = +1 |
| scaffold_3 | 8744 | 8965 | 17 | hypothetical protein | hypothetical protein QT08_C0015G0036 [*archaeon GW2011_AR17*] | 61% | 0.19 | 37% | gi\|735018128\|KHO52065.1 | 222 | BGC0000974\|c1\|47269-51879\|+\|no_locus_tag\|non-ribosomal_peptide_synthetase\|CBD77749.1 Length=1536 | Crocacin biosynthetic gene cluster | Chondromyces crocatus  Bacteria; Proteobacteria; Deltaproteobacteria; Myxococcales;  Sorangiineae; Polyangiaceae; Chondromyces. | 26.2 | 2.7 | Identities = 12/34 (35%), Positives = 14/34 (41%), Gaps = 0/34 (0%)  Frame = +3 |
| scaffold_3 | 9586 | 8936 | 18 | hypothetical protein | conserved Plasmodium protein, unknown function [*Plasmodium ovale*] | 85% | 1e-10 | 26% | gi\|1070687126\|SCP04256.1 | 651 | BGC0000022\|c1\|5855-8208\|-\|AN1029.2\|hypothetical_protein\|EAA65597.1 Length=666 | Asperfuranone biosynthetic gene cluster | Aspergillus nidulans FGSC A4  Eukaryota; Fungi; Dikarya; Ascomycota; Pezizomycotina;  Eurotiomycetes; Eurotiomycetidae; Eurotiales; Aspergillaceae;  Aspergillus. | 28.9 | 3.3 | Identities = 14/54 (26%), Positives = 29/54 (54%), Gaps = 4/54 (7%)  Frame = +1 |
| scaffold_3 | 11312 | 9570 | 19 | hypothetical protein | hypothetical protein [*Dorea longicatena*] | 22% | 0.15 | 30% | gi\|942068586\|WP_055284466.1 | 1743 | BGC0001033\|c1\|39884-48106\|+\|ERIC2_c18110\|non-ribosomal_peptide_ligase_domain_protein\|AHD05621.1 Length=2740 | Paenilamicin biosynthetic gene cluster | Paenibacillus larvae subsp. larvae DSM 25430  Bacteria; Firmicutes; Bacilli; Bacillales; Paenibacillaceae;  Paenibacillus. | 33.9 | 0.45 | Identities = 23/61 (38%), Positives = 34/61 (56%), Gaps = 5/61 (8%)  Frame = +1 |
| scaffold_7 | 29 | 160 | 20 | hypothetical protein | non-ribosomal peptide synthetase [*Mycobacterium minnesotense*] | 67% | 0.18 | 45% | gi\|1178501113\|WP_083022343.1 | 132 | BGC0001025\|c1\|30409-57357\|-\|MXAN_3935\|non-ribosomal_peptide_synthase/polyketide_synthase_Ta1\|ABF85931.1 Length=8982 | Myxovirescin biosynthetic gene cluster | Myxococcus xanthus DK 1622  Bacteria; Proteobacteria; Deltaproteobacteria; Myxococcales;  Cystobacterineae; Myxococcaceae; Myxococcus. | 23.1 | 8.7 | Identities = 6/6 (100%), Positives = 6/6 (100%), Gaps = 0/6 (0%)  Frame = -1 |
| scaffold_7 | 456 | 268 | 21 | hypothetical protein | ribbon-helix-helix protein, CopG family, partial [*Klebsiella quasipneumoniae*] | 62% | 0.002 | 46% | gi\|1252880495\|WP_096834078.1 | 189 | BGC0000883\|c1\|7288-8346\|+\|no_locus_tag\|hypothetical_protein_Radical_SAM\|AGG35696.1 Length=352 | Arginomycin biosynthetic gene cluster | Streptomyces arginensis  Bacteria; Actinobacteria; Actinobacteridae; Actinomycetales;  Streptomycineae; Streptomycetaceae; Streptomyces. | 30.4 | 0.057 | Identities = 20/50 (40%), Positives = 27/50 (54%), Gaps = 9/50 (18%)  Frame = +1 |
| scaffold_7 | 539 | 1261 | 22 | hypothetical protein | hypothetical protein [*Streptomyces graminilatus*] | 61% | 1.1 | 27% | gi\|943896723\|WP_055534994.1 | 723 | BGC0000967\|c1\|31828-51879\|+\|no_locus_tag\|CalB\|BAP05590.1 Length=6683 | Calyculin biosynthetic gene cluster | uncultured Candidatus Entotheonella sp.  Bacteria; Proteobacteria; Deltaproteobacteria; Candidatus  Entotheonella; environmental samples. | 34.7 | 0.078 | Identities = 29/107 (27%), Positives = 51/107 (48%), Gaps = 7/107 (7%)  Frame = +1 |
| scaffold_7 | 1278 | 1523 | 23 | hypothetical protein | hypothetical protein [*Catelliglobosispora koreensis*] | 82% | 0.30 | 34% | gi\|522013844\|WP_020525115.1 | 246 | BGC0000052\|c1\|36213-56468\|+\|no_locus_tag\|polyketide_synthase_type_I\|AAX98186.1 Length=6751 | ECO-02301 biosynthetic gene cluster | Streptomyces aizunensis  Bacteria; Actinobacteria; Actinobacteridae; Actinomycetales;  Streptomycineae; Streptomycetaceae; Streptomyces. | 27.3 | 1.3 | Identities = 11/33 (33%), Positives = 17/33 (52%), Gaps = 0/33 (0%)  Frame = +2 |
| scaffold_7 | 1668 | 1474 | 24 | hypothetical protein | hypothetical protein PFMALIP_02734 [*Plasmodium falciparum MaliPS096_E11*] | 90% | 0.034 | 38% | gi\|574987966\|ETW49231.1 | 195 | BGC0000336\|c1\|111553-112353\|+\|no_locus_tag\|putative_ABC_transporter_permease\|AAX31570.1 Length=266 | Daptomycin biosynthetic gene cluster | Streptomyces roseosporus NRRL 11379  Bacteria; Actinobacteria; Actinobacteridae; Actinomycetales;  Streptomycineae; Streptomycetaceae; Streptomyces. | 25.8 | 2.1 | Identities = 12/25 (48%), Positives = 15/25 (60%), Gaps = 5/25 (20%)  Frame = -2 |
| scaffold_7 | 1646 | 2380 | 25 | hypothetical protein | hypothetical protein HRbin13_00748 [*bacterium HR13*] | 15% | 0.61 | 38% | gi\|1286947381\|GBC88624.1 | 735 | BGC0000073\|c1\|66650-78079\|+\|no_locus_tag\|type_I_polyketide_synthase\|BAF02925.1 Length=3809 | Halstoctacosanolide biosynthetic gene cluster | Streptomyces halstedii  Bacteria; Actinobacteria; Actinobacteridae; Actinomycetales;  Streptomycineae; Streptomycetaceae; Streptomyces. | 30 | 1.9 | Identities = 14/32 (44%), Positives = 16/32 (50%), Gaps = 0/32 (0%)  Frame = +1 |
| scaffold_7 | 2377 | 2742 | 26 | hypothetical protein | hypothetical protein [*Bacillus cereus*] | 85% | 2.8 | 24% | gi\|447188295\|WP_001265551.1 | 366 | BGC0001045\|c1\|16340-19867\|+\|no_locus_tag\|polyketide_synthase_SpiC1\|AFR69333.1 Length=1175 | Spiruchostatin biosynthetic gene cluster | Pseudomonas sp. Q71576  Bacteria; Proteobacteria; Gammaproteobacteria; Pseudomonadales;  Pseudomonadaceae; Pseudomonas. | 27.7 | 2.7 | Identities = 22/69 (32%), Positives = 30/69 (43%), Gaps = 1/69 (1%)  Frame = +1 |
| scaffold_7 | 2755 | 3114 | 27 | hypothetical protein | hypothetical protein [*Bacillus cereus*] | 83% | 0.003 | 31% | gi\|518142806\|WP_019313014.1 | 360 | BGC0000333\|c1\|17588-18382\|+\|Sare_4561\|Phytanoyl-CoA_dioxygenase\|ABW00330.1 Length=264 | Cyclomarin biosynthetic gene cluster | Salinispora arenicola CNS-205  Bacteria; Actinobacteria; Actinobacteridae; Actinomycetales;  Micromonosporineae; Micromonosporaceae; Salinispora. | 26.2 | 7.1 | Identities = 17/47 (36%), Positives = 22/47 (47%), Gaps = 0/47 (0%)  Frame = -2 |
| scaffold_7 | 3115 | 3726 | 28 | hypothetical protein | hypothetical protein [*Halapricum salinum*] | 91% | 2e-24 | 33% | gi\|910078721\|WP_049994636.1 | 612 | BGC0000021\|c1\|13035-14018\|-\|no_locus_tag\|rebeccamycin_sugar_4'-O-methyltransferase\|AEP40916.1 Length=327 | Apoptolidin biosynthetic gene cluster | Nocardiopsis sp. FU40  Bacteria; Actinobacteria; Actinobacteridae; Actinomycetales;  Streptosporangineae; Nocardiopsaceae; Nocardiopsis. | 30.4 | 0.9 | Identities = 17/36 (47%), Positives = 17/36 (47%), Gaps = 0/36 (0%)  Frame = +1 |
| scaffold_7 | 3719 | 4762 | 29 | Glycosyltransferase | glycosyltransferase family 4 protein [*Enhydrobacter aerosaccus*] | 98% | 1e-30 | 28% | gi\|1194598116\|WP_085933361.1 | 1044 | BGC0001195\|c1\|74606-75901\|+\|no_locus_tag\|glycosyltransferase\|AJW65414.1 Length=431 | Nocardiopsin biosynthetic gene cluster | Nocardiopsis sp. CMB-M0232  Bacteria; Actinobacteria; Streptosporangiales; Nocardiopsaceae;  Nocardiopsis. | 55.8 | 2.00E-08 | Identities = 44/169 (26%), Positives = 79/169 (47%), Gaps = 14/169 (8%)  Frame = +1 |
| scaffold_7 | 4817 | 5599 | 30 | hypothetical protein | phosphodiesterase [*Natrialba sp. SSL1*] | 63% | 2.2 | 24% | gi\|1100835585\|WP_071402097.1 | 783 | BGC0000457\|c1\|2048-6040\|-\|RHE_PF00457\|vicibactin_biosynthesis_non-ribosomal_peptide_synthase_protein\|ABC94347.1 Length=1330 | Vicibactin biosynthetic gene cluster | Rhizobium etli CFN 42  Bacteria; Proteobacteria; Alphaproteobacteria; Rhizobiales;  Rhizobiaceae; Rhizobium/Agrobacterium group; Rhizobium. | 28.1 | 9.2 | Identities = 34/141 (24%), Positives = 49/141 (35%), Gaps = 31/141 (22%)  Frame = +1 |
| scaffold_7 | 5592 | 6602 | 31 | hypothetical protein | hypothetical protein AMJ42_02660 [*Deltaproteobacteria bacterium DG_8*] | 74% | 9e-08 | 27% | gi\|931367356\|KPJ58761.1 | 1011 | BGC0000880\|c1\|6657-8204\|+\|no_locus_tag\|TunH\|ADP94228.1 Length=515 | Tunicamycin biosynthetic gene cluster | Streptomyces chartreusis NRRL 3882  Bacteria; Actinobacteria; Actinobacteridae; Actinomycetales;  Streptomycineae; Streptomycetaceae; Streptomyces. | 36.2 | 0.038 | Identities = 30/94 (32%), Positives = 48/94 (51%), Gaps = 12/94 (13%)  Frame = +1 |

**Table S3:** **Predicted phylogeny as computed by MEGAN for the insert DNA of fosmid clones 14-7E and 10-2G*.** *The count of assigned PEGs to each taxon is indicated in parentheses.

| **Clone** | **Other** | **Domain** | **Phylum** | **Class** | **Order** | **Family** | **Species** |
| --- | --- | --- | --- | --- | --- | --- | --- |
| **10-2G** | Cellular organisms (3) |  |  |  |  |  |  |
|  | No hits (27) |  |  |  |  |  |  |
| **14-7E** | Cellular organisms (28) | *Bacteria (5)* | *Proteobacteria (1)* | *Deltaproteobacteria (1)* | *Desulfobacterales (1)* | *Desulfobacteraceae (1)* | *Desulfatirhabdium butyrativorans (1)* |
|  |  |  | *Firmicutes (2)* | *Bacilli (1)* | *Lactobacillales (1)* | *Streptococcaceae (1)* | *Streptococcus pneumoniae (1)* |
|  |  |  |  | *Clostridia (1)* | *Clostridiales (1)* | *Clostridiaceae (1)* | *Clostridium pasteurianum (1)* |
|  |  | *Archaea (8)* | *Euryarchaeota (6)* | *Halobacteria (1)* | *Natrialbales (1)* | *Natrialbaceae (1)* | *Halovivax asiaticus (1)* |
|  |  |  |  | *Methanococci (1)* | *Methanococcales (1)* | *Methanococcaceae (1)* | *Methanococcus maripaludis (1)* |
|  |  |  |  | *Methanomicrobia (2)* | *Methanosarcinales (2)* | *Methanosarcinaceae (2)* | *Methanosarcina acetivorans (1)* |
|  |  |  |  |  |  |  | *Methanosarcina soligelidi (1)* |
|  |  |  |  | *Thermococci (1)* | *Thermococcales (1)* | *Thermococcaceae (1)* | *Palaeococcus pacificus (1)* |
|  |  | *Eukaryota (1)* | *Platyhelminthes (1)* | *Cestoda (1)* | *Cyclophyllidea (1)* | *Taeniidae (1)* | *Echinococcus multilocularis (1)* |
|  | No hits (261) |  |  |  |  |  |  |

**Figure S4: Phylogenetic trees as predicted by MEGAN for the insert DNA of (a) 14-7E and (b) 10-2G.**

(a)

(b)

**Table S4: PEGs annotated in the inserts of 14-7E and 10-2G with hits from BLASTX against MIBiG biosynthetic gene clusters with E-value ≤ 0.005.**

| **Clone insert** | **PEG** | **Biosynthetic Gene Cluster (BGC) reference in MIBiG** | **E-Value** | **Class** | **Reference** |
| --- | --- | --- | --- | --- | --- |
| **14-7E** | 5 | Carotenoid | 5.00E-19 | Terpene | [1] |
|  | 54 | Mannopeptimycin | 2.00E-32 | Non-ribosomal peptide (NRP) | [2] |
|  | 73 | Streptide | 2.00E-05 | Ribosomally synthesized and post-translationally modified peptide (RiPP) | [3] |
|  | 96 | Herboxidiene | 5.00E-04 | Polyketide | [4] |
|  | 99 | Cepacian | 1.00E-26 | Saccharide | [5] |
|  | 122 | Lipopolysaccharide | 8.00E-08 | Saccharide | [6] |
|  | 124 | Alkyl-O-Dihydrogeranyl-Methoxyhydroquinones | 7.00E-05 | Polyketide / Terpene | [7] |
|  | 159 | Ustilagic acid | 1.00E-15 | Polyketide | [8] |
|  | 177 | AT2433 | 2.00E-04 | Alkaloid | [9] |
|  | 200 | Leinamycin | 4.00E-10 | NRP / Polyketide | [10] |
|  | 202 | Carotenoid | 9.00E-17 | Terpene | [1] |
|  | 216 | Phosphonoglycans | 5.00E-05 | Saccharide | [11] |
|  | 218 | Pentalenolactone | 2.00E-72 | Terpene | [12] |
|  | 219 | Pentalenolactone | 7.00E-12 | Terpene | [12] |
|  | 272 | Carotenoid | 4.00E-07 | Terpene | [1] |
|  | 274 | SCO-2138 | 1.00E-05 | RiPP | [13] |
|  | 287 | Cepacian | 4.00E-15 | Saccharide | [5] |
| **10-2G** | 3 | Staurosporine | 2.00E-05 | Alkaloid | [14] |
|  | 5 | Neocarzinostatin | 1.00E-11 | Polyketide | [15] |
|  | 6 | S-layer glycan | 3.00E-09 | Saccharide | [16] |
|  | 9 | Lasalocid | 0.003 | Polyketide | [17] |
|  | 29 | Nocardiopsin | 2.00E-08 | NRP / Polyketide | [18] |

**Figure S5: Representative photos of MCF-7 (A) and 1BR hTERT (B) cells after exposure to 14-7E lysates for 48 hrs (200X magnification).**

**References:**

1. Tao L, Yao H, Cheng Q. Genes from a Dietzia sp. for synthesis of C40and C50β-cyclic carotenoids. Gene. 2007;386:90–7.

2. Magarvey NA, Haltli B, He M, Greenstein M, Hucul JA. Biosynthetic Pathway for Mannopeptimycins , Lipoglycopeptide Antibiotics Active against Drug-Resistant Gram-Positive Pathogens. 2006;50:2167–77.

3. Schramma KR, Bushin LB, Seyedsayamdost MR. Structure and biosynthesis of a macrocyclic peptide containing an unprecedented lysine-to-tryptophan crosslink. Nat Chem [Internet]. Nature Publishing Group; 2015;7:431–7. Available from: http://dx.doi.org/10.1038/nchem.2237

4. Shao L, Zi J, Zeng J, Zhan J. Identification of the Herboxidiene Biosynthetic Gene Cluster in Streptomyces chromofuscus ATCC 49982. 2012;2034–8.

5. Moreira LM, Videira PA, Sousa SA, Leitão JH, Cunha M V., Sá-Correia I. Identification and physical organization of the gene cluster involved in the biosynthesis of Burkholderia cepacia complex exopolysaccharide. Biochem Biophys Res Commun. 2003;312:323–33.

6. Grozdanov L, Zähringer U, Blum-Oehler G, Brade L, Henne A, Knirel YA, et al. A single nucleotide exchange in the wzy gene is responsible for the semirough O6 lipopolysaccharide phenotype and serum sensitivity of Escherichia coli strain Nissle 1917. J Bacteriol. 2002;184:5912–25.

7. Awakawa T, Fujita N, Hayakawa M, Ohnishi Y. Characterization of the Biosynthesis Gene Cluster for Alkyl- O -Dihydrogeranyl-Methoxyhydroquinones in Actinoplanes missouriensis. 2011;8511:439–48.

8. Teichmann B, Liu L, Schink KO, Bo M. Activation of the Ustilagic Acid Biosynthesis Gene Cluster in Ustilago maydis by the C 2 H 2 Zinc Finger Transcription Factor Rua1 ᰔ. 2010;76:2633–40.

9. Gao Q, Zhang C, Blanchard S, Thorson JS. Deciphering Indolocarbazole and Enediyne Aminodideoxypentose Biosynthesis through Comparative Genomics : Insights from the AT2433 Biosynthetic Locus. 2006;733–43.

10. Cheng Y, Tang G, Shen B. Identification and Localization of the Gene Cluster Encoding Biosynthesis of the Antitumor Macrolactam Leinamycin in Streptomyces atroolivaceus S-140. 2002;184:7013–24.

11. Yu X, Price NPJ, Evans BS, Metcalf WW. Purification and Characterization of Phosphonoglycans from Glycomyces sp. Strain NRRL B-16210 and Stackebrandtia nassauensis NRRL B-16338. 2014;196:1768–79.

12. Tetzlaff CN, You Z, Cane DE, Takamatsu S, Omura S, Ikeda H. A Gene Cluster for Biosynthesis of the Sesquiterpenoid Antibiotic Pentalenolactone in Streptomyces avermitilis. 2008;45:6179–86.

13. Kersten RD, Yang Y-L, Xu Y, Cimermancic P, Nam S-J, Fenical W, et al. A mass spectrometry–guided genome mining approach for natural product peptidogenomics. Nat Chem Biol [Internet]. Nature Publishing Group; 2011;7:794–802. Available from: http://www.nature.com/doifinder/10.1038/nchembio.684

14. Medema MH, Trefzer A, Kovalchuk A, van den Berg M, Mueller U, Heijne W, et al. The Sequence of a 1.8-Mb Bacterial Linear Plasmid Reveals a Rich Evolutionary Reservoir of Secondary Metabolic Pathways. 2010;2:212–24.

15. Liu W, Nonaka K, Nie L, Zhang J, Christenson SD, Bae J, et al. The Neocarzinostatin Biosynthetic Gene Cluster from Streptomyces carzinostaticus ATCC 15944 Involving Two Iterative Type I Polyketide Synthases. 2005;12:293–302.

16. Kneidinger B, Graninger M, Adam G, Puchberger M, Kosma P, Zayni S, et al. Identification of Two GDP-6-deoxy- D - lyxo -4-hexulose Reductases Synthesizing GDP- D -rhamnose in Aneurinibacillus thermoaerophilus L420-91 T *. 2001;276:5577–83.

17. Migita A, Watanabe M, Hirose Y, Watanabe K, Tokiwano T, Kinashi H, et al. Identification of a Gene Cluster of Polyether Antibiotic Lasalocid from Streptomyces lasaliensis. 2009;73:169–76.

18. Bis DM, Ban YH, James ED, Alqahtani N, Viswanathan R, Lane AL. Characterization of the Nocardiopsin Biosynthetic Gene Cluster Reveals Similarities to and Differences from the Rapamycin and FK-506 Pathways. ChemBioChem. 2015;16:990–7.
